# Component of Cannabis, Cannabidiol, as a Possible Drug against the Cytotoxicity of Aβ(31–35) and Aβ(25–35) Peptides: An Investigation by Molecular Dynamics and Well-Tempered Metadynamics Simulations

**DOI:** 10.1021/acschemneuro.0c00692

**Published:** 2021-02-05

**Authors:** Wojciech Chrobak, Dawid Wojciech Pacut, Fredrik Blomgren, Alexander Rodin, Jan Swenson, Inna Ermilova

**Affiliations:** Department of Physics, Chalmers University of Technology, 412 96 Gothenburg, Sweden

**Keywords:** Cannabis, cannabidiol, Alzheimer’s disease, molecular dynamics, metadynamics

## Abstract

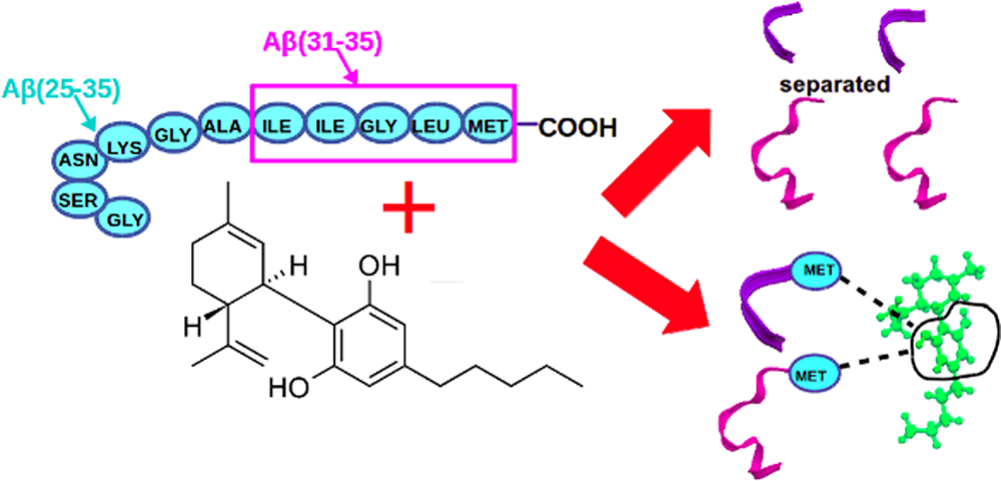

In this work cannabidiol (CBD) was investigated as a possible drug against the cytotoxicity of Aβ(31–35) and Aβ(25–35) peptides with the help of atomistic molecular dynamics (MD) and well-tempered metadynamics simulations. Four interrelated mechanisms of possible actions of CBD are proposed from our computations. This implies that one mechanism can be a cause or/and a consequence of another. CBD is able to decrease the aggregation of peptides at certain concentrations of compounds in water. This particular action is more prominent for Aβ(25–35), since originally Aβ(31–35) did not exhibit aggregation properties in aqueous solutions. Interactions of CBD with the peptides affect secondary structures of the latter ones. Clusters of CBD are seen as possible adsorbents of Aβ(31–35) and Aβ(25–35) since peptides are tending to aggregate around them. And last but not least, CBD exhibits binding to *MET*_35_. All four mechanisms of actions can possibly inhibit the Aβ-cytotoxicity as discussed in this paper. Moreover, the amount of water also played a role in peptide clustering: with a growing concentration of peptides in water without a drug, the aggregation of both Aβ(31–35) and Aβ(25–35) increased. The number of hydrogen bonds between peptides and water was significantly higher for simulations with Aβ(25–35) at the higher concentration of peptides, while for Aβ(31–35) that difference was rather insignificant. The presence of CBD did not substantially affect the number of hydrogen bonds in the simulated systems.

## Introduction

Positive pharmacological properties of cannabis have been known for more than one century.^1−3^ Historically different types of cannabis plants were successfully used for treating tetanus,^2,4^ various types of pain,^5,6^ rheumatism,^7^ cholera,^1,8^ etc. Later compounds extracted from cannabis plants such as *trans*-Δ^9^-tetrahydrocannabinol (THC-9), cannabigerol, and cannabidiol (CBD) have shown a good potential in treating such diseases as Alzheimer’s,^9^ Parkinson’s,^10,11^ autism,^12^ cholitis,^13^ cancer,^14,15^ post Ebola syndrome,^16^ and many others. However, this very long history of successful applications of cannabis plants and their compounds did not help in disclosing the exact mechanisms of their actions.^17−20^

Nowadays the most commercially trending component of cannabis is CBD, since its psychoactivity is not the same as of THC-9.^21,22^ Moreover, due to the yearly increase of cases of neurodegenerative diseases,^23−25^ CBD becomes a very attractive drug because it has already shown the great potential against them in various experimental studies.^26−30^ For instance, G. Esposito et al.^31^ showed on rat primary astroglial cultures that CBD could reduce the inflammation which was Aβ-induced. R. Libro et al.^32^ discovered that CBD was involved in the prevention of the expression of proteins potentially involved in tau phosphorylation and Aβ-peptide production. Long-term treatment of transgenic Alzheimer’s disease mice with CBD prevented the development of social recognition memory deficits according to D. Cheng et al.^33^

Thus, CBD can act in many different ways against neurodegenerative diseases: it can prevent the production of Aβ peptides, and it can probably act on cell membranes, peptide secondary structures, the ability of peptides to aggregate, etc.^30,31^ In this work the accent is on the amyloid hypothesis, which says that the development of Alzheimer’s and Parkinson’s diseases happens due to the aggregation of Aβ peptides in an extracellular space.^34−37^ Such aggregates build plaques on cell membranes which cause apoptosis (cell’s death) later.

**Figure 1:**
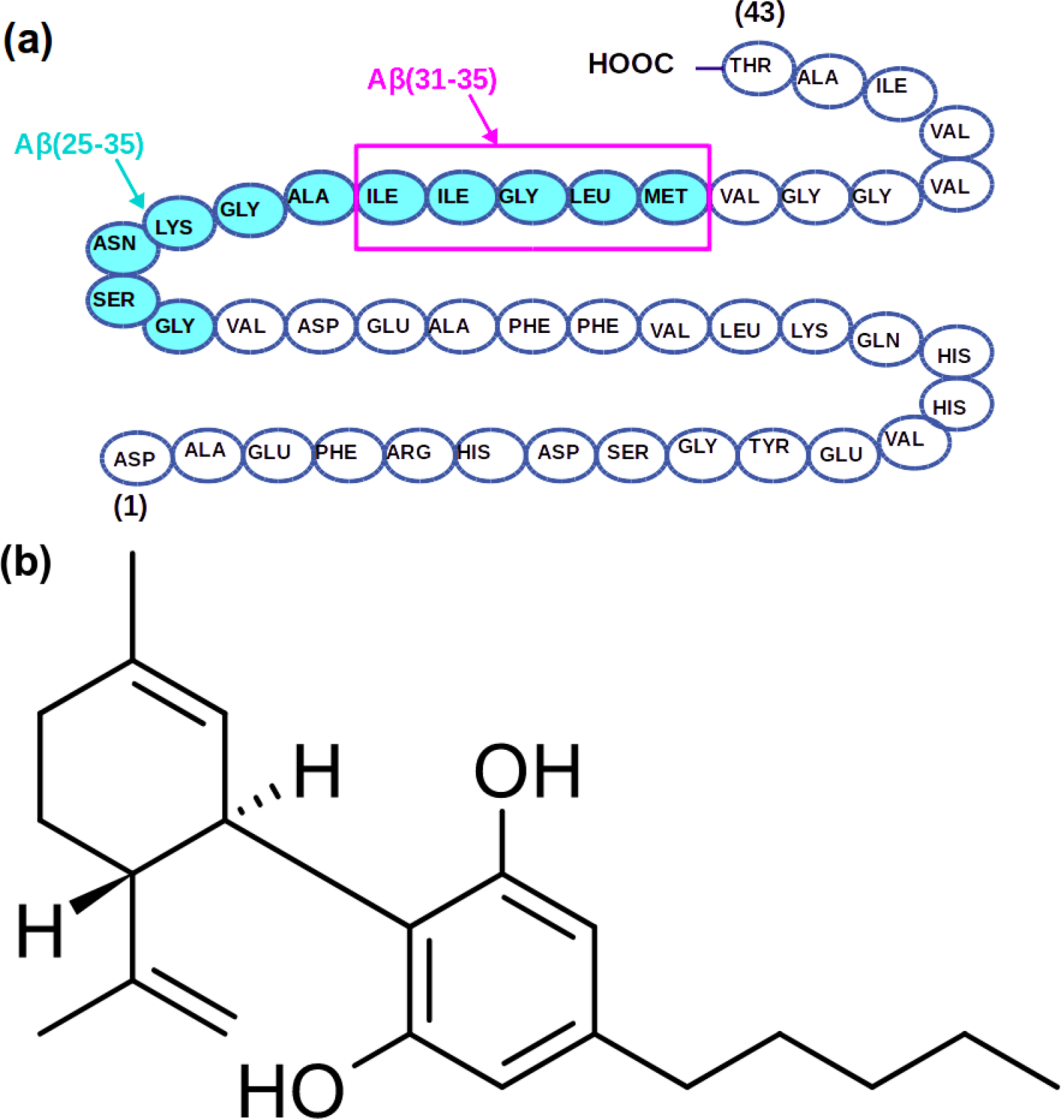
Molecules used in simulations: (a) the whole primary structure of the Aβ(1–43) peptide with denoted sequences of Aβ(25–35) (cyan circles) and Aβ(31–35) (magenta rectangle) peptides; (b) CBD molecule.

There were different lengths of peptides found in the human brain affected by Alzheimer’s and Parkinson’s diseases.^38,39^ Most of them belonged to the sequence Aβ(1–43), but they were not equally cytotoxic.^40^ The importance of different amino acid residues in the sequence and the role of their positions in peptides on cytotoxicity have been investigated by many research groups. For example, Aβ(25–35) (see Figure 1a) is considered as a more toxic part of the sequence than others.^41−43^ This part is known to aggregate within hours.^44^ It is physiologically present in elderly people,^45^ and it retains the toxicity of the full length of the peptide Aβ(1–42).^38^

Another interesting part of the sequence is Aβ(31–35) (see Figure 1a). It is known to induce cell apoptosis in isolated rat brain mitochondria^46^ and in cultured cortical neurons of newborn mice.^47^ In comparison with Aβ(25–35), Aβ(31–35) was acting differently in inducing neurotoxicity of PC12 cells.^48^ According to F. Misiti et al.,^48^ Aβ(31–35) was acting via an apoptotic cell death pathway, embracing caspase activation and DNA fragmentation. Aβ(25–35) was inducing neurotoxicity by adherent cell count without associating with any biochemical features of apoptosis.^48^ Moreover, in the same study it was noted that the C-terminus was involved in toxicity mechanisms of both peptides but in different ways.

The short lengths of these peptides together with their similarities in terms of sequences and their different ways of inducing the cytotoxicity make them attractive candidates together with CBD (Figure 1b) for theoretical studies using classical atomistic MD and well-tempered metadynamics simulations, particularly, because such studies have not been conducted for mixtures of these molecules earlier. CBD has been studied *in silico* only with Aβ(1–42), but those studies were a combination of molecular docking and quantum chemical calculations employing density functional theory.^49^ S. Das et al.^49^ compared neuroprotective properties of poyphenolic ligands and discovered that they could inhibit the aggregation of Aβ(1–42). Other computational works involving CBD were performed with other proteins, CBD receptors, and lipids.^50−53^

There are not so many works with Aβ(31–35) or Aβ(25–35) investigated by atomistic MD simulations. Only a few studies have been conducted on Aβ(25–35), and surprisingly, not even a single work has been carried out with the short Aβ(31–35). H.-H. G. Tsai et al.^54^ performed replica exchange molecular dynamics simulations where they investigated the insertion of Aβ(25–35) and its mutants in a membrane. However, both membranes and water models were implicit, which does not explain the exact mechanisms of peptide–membrane interactions. S.-W. Lee et al.^55^ investigated the behavior of Aβ(25–35) in a trifluoroethanol solution in order to understand the effect of the solvent on the conformational distribution of the peptide. They found that trifluoroethanol can promote the formation of α-helical structures.^55^ I. Ermilova et al.^56^ studied the behavior of Aβ(25–35) in lipid bilayers with and without cholesterol. They discovered that *MET*_35_ in the C-terminus plays an important role in possible hydrogen bond formations between lipid head-groups and peptides. Moreover, according to their findings, Aβ(25–35) exhibits aggregation properties on membranes loaded with cholesterol, in agreement with latter experimental studies by T. Murugova et al.^57^

The goal of this work is to investigate interactions between short Aβ peptides (Aβ(31–35) and Aβ(25–35)) and CBD and find their possible relations to cytotoxic properties of peptides, using atomistic MD and well-tempered metadynamics simulations. From atomistic MD simulations information about peptide aggregation, their secondary structures and associations with CBD molecules can be obtained. Furthermore, since in earlier experimental and computational studies *MET*_35_ was pointed out as the amino acid residue that affected the cytotoxicity most,^56,58−60^ it is of interest to see if CBD can bind to it. Such a binding could imply a possible inhibition of toxicity of peptides.

Additionally, the influence of the amount of water on clustering of peptides is considered for the investigation, since water is playing an important role in the protein aggregation.^61−63^ Such a phenomenon can be studied by increasing the amount of CBD and peptides at the same ratios with a constant number of water molecules in simulations.

In the case of well-tempered metadynamics^64−66^ simulations the idea is to investigate the aggregation between compounds from a thermodynamic point of view, depending on the amount of CBD molecules in the system. Such calculations give an opportunity to select parameters of systems (collective variables, CVs) and calculate the potential mean force (PMF) of the system, depending on them, and integrate the resulting PMF in binding free energies. As CVs it is convenient to select distances between the molecular centers of mass. Choosing different variables for various parts of the molecules would lead to too many variables and constraints and, as a result, to a very time-consuming simulation. If a value of the integral is negative (Δ*G*_bind_ < 0), then two molecules can bind; otherwise, the two molecules are coexisting separately. Well-tempered metadynamics is a time-consuming method; however, it is known for not pushing the calculation to unattractive high free energy regions.^67^ This advantage was the reason behind the choice of the method in the current project. Thus, for Aβ(31–35) and Aβ(25–35) it is feasible to calculate PMF and binding free energies depending on distances between peptides and CBD molecules, which could explain the peptide aggregation process and its possible inhibition by CBD.

## Results and Discussion

### Parametrization: Partial Atomic Charges for Cannabidiol

**Figure 2:**
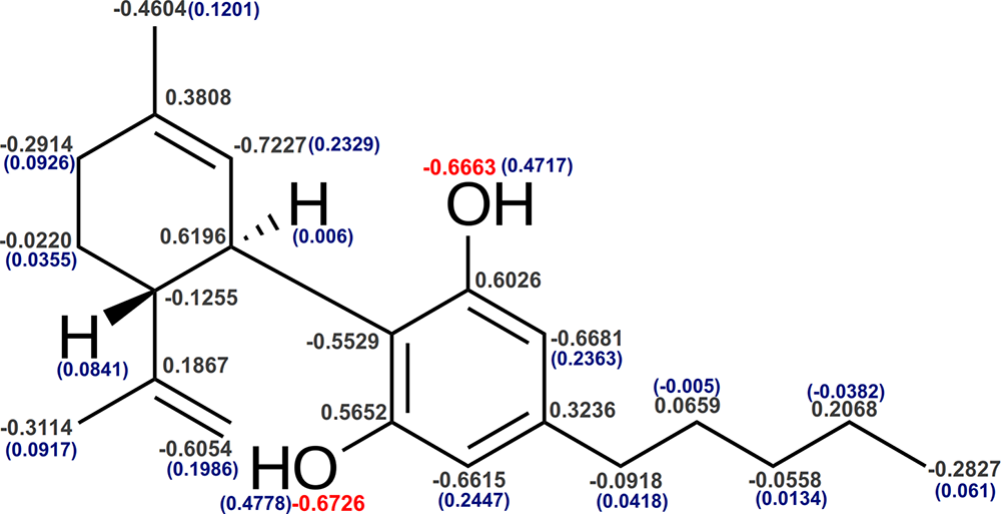
CBD molecule with derived partial atomic charges. Here black values are charges for carbon atoms, dark blue values are charges for hydrogen atoms, and red values are charges for oxygen atoms.

The computed final partial atomic charges are demonstrated in Figure 2. Those charges were used for CBD molecule in all simulations.

### Radial Distribution Functions, Contact Maps, Hydrogen Bonds

Radial distribution functions (RDFs) are characteristics of a system which provide information about interactions and correlations between different components in the system. For instance, RDFs between molecular center of mass can answer the question if there is any affinity between certain molecules.

**Figure 3:**
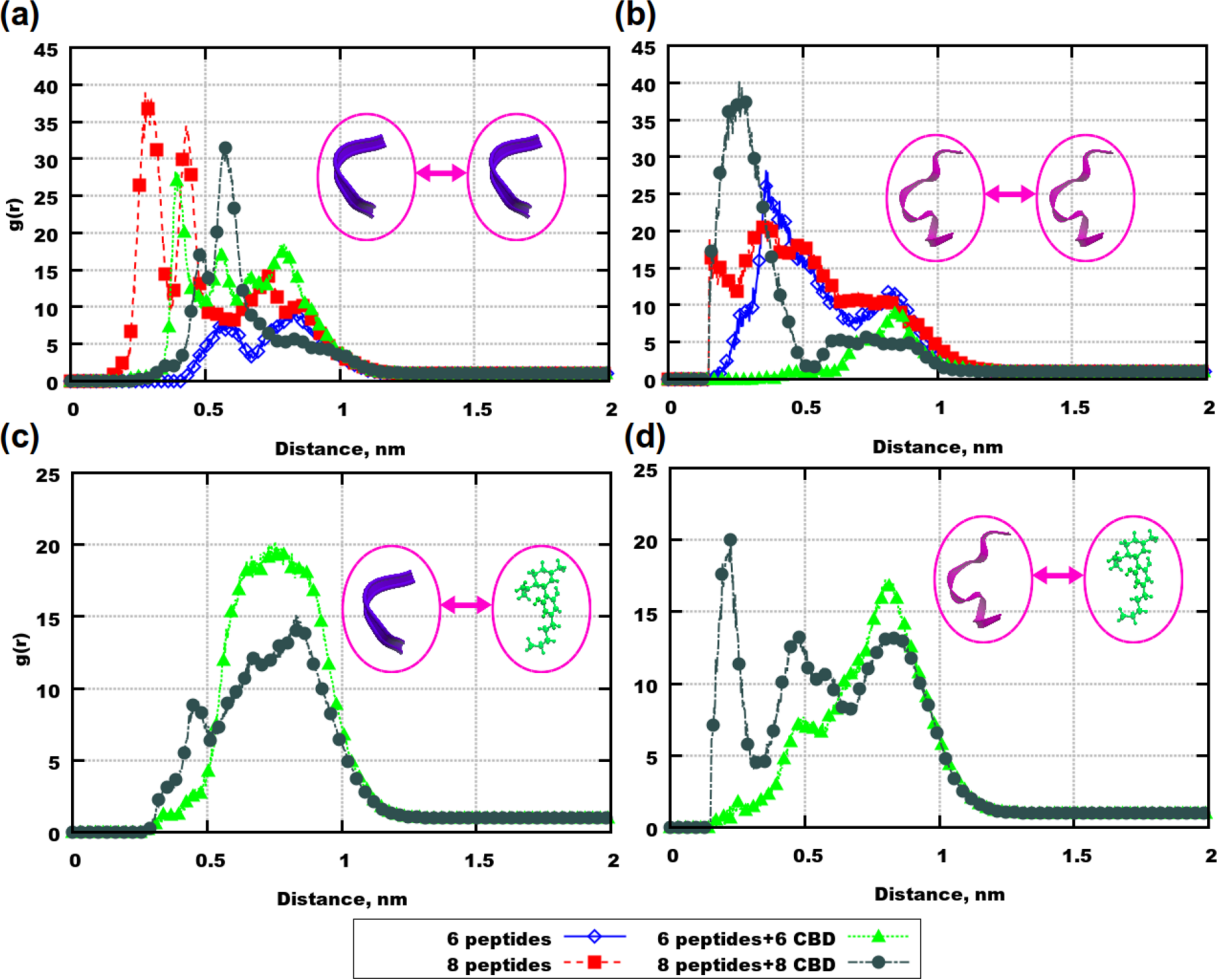
RDFs between molecular center of mass, computed over 250 ns: (a) Aβ(31–35) and Aβ(31–35); (b) Aβ(25–35) and Aβ(25–35); (c) Aβ(31–35) and CBD; (d) Aβ(25–35) and CBD. The visualization is the following: purple ribbons are Aβ(25–35), blue ribbons are Aβ(31–35), and green molecules are CBD. The sizes of molecules were rescaled for the purpose of the schematic visualization.

Figure 3a presents RDFs between the molecular centers of mass of Aβ(31–35) and CBD. In simulations with 6 molecules the presence of CBD promotes aggregation of the peptides, compared to the system containing no CBD. When 8 molecules are present, the situation is changing: the presence of CBD is inhibiting the clustering of Aβ(31–35). These two statements can even be confirmed by RDFs computed for different time intervals (Figure S1 in Supporting Information): the value of the function is higher and the first peak appears at a closer distance in the end of the simulation for the system with 6 CBD (Figure S1a,b in Supporting Information), while in the case of 8 molecules an opposite trend is observed (Figure S1c,d in Supporting Information). RDFs between Aβ(31–35) and CBD show that there can be a strong association between CBD molecules and peptides in the system with 8 molecules, since the first peak appears at a distance of less than 0.5 nm, as seen in Figure 3c. However, the highest value of the RDF is observed at a distance of 0.9 nm between the molecules. In the simulation with 6 molecules the only peak is observed at a distance of 0.75 nm. Considering that Aβ(31–35) has a length of around 1.35 nm and the length of the CBD molecule is about 1.35 nm, one can conclude that there can be strong associations between CBD and the peptides in both systems.

In the case of Aβ(25–35) the inhibition of aggregation is observed for systems with 6 molecules of each compound when CBD is present in the simulation, while with 8 molecules the promotion of clustering in the presence of CBD can be seen in Figure 3b. Figure S2 in Supporting Information confirms that with a lesser amount of molecules the inhibition of aggregation of Aβ(25–35) is highly likely to occur.

More details of the peptide aggregation can be seen on contact maps. Figures S3–S10 in Supporting Information demonstrate such contact maps for peptides taken after 3 time intervals (150 ns, 200 ns, and 250 ns) during production runs using the VMD software.^68^

For Aβ(31–35) less contacts (gray points) are observed in the system with 6 peptides than in the system with 6 peptides and 6 CBD molecules (Figures S3 and S4), while in simulations with 8 molecules of peptides and peptides with CBD the number of gray points is smaller in the system containing the peptides and the drug (Figures S5 and S6).

Contact maps for systems containing 6 Aβ(25–35) show a higher number of contacts when CBD is absent (Figures S7 and S8). A similar effect of the presence of CBD can be seen even for the systems with 8 molecules: more gray points when the drug is not in the system and less when it was added (Figures S9 and S10).

Considering RDFs between peptides and CBD, Figure 3d shows a strong affinity between the molecules in the system with 8 CBD and 8 Aβ(25–35) and a weaker one in the similar system with 6 molecules of each compound. However, Aβ(25–35) has a double length of Aβ(31–35), which means that it can explore a larger variety of conformations, and therefore, the coordinates of the centers of mass and the radius of gyration can fluctuate. For instance, from our of knowledge of lengths of stretched peptides it follows that the radius of gyration for Aβ(31–35) can be smaller than for Aβ(25–35). Considering the full length of the peptide, one can still conclude that there is an affinity between CBD and Aβ(25–35) rather than an aversion. Figure S11 in Supporting Information demonstrates the evolution of RDFs between CBD and peptides in various time intervals, and Figures S12–S19 show changes in radius of gyration for every peptide during production runs.

**Figure 4:**
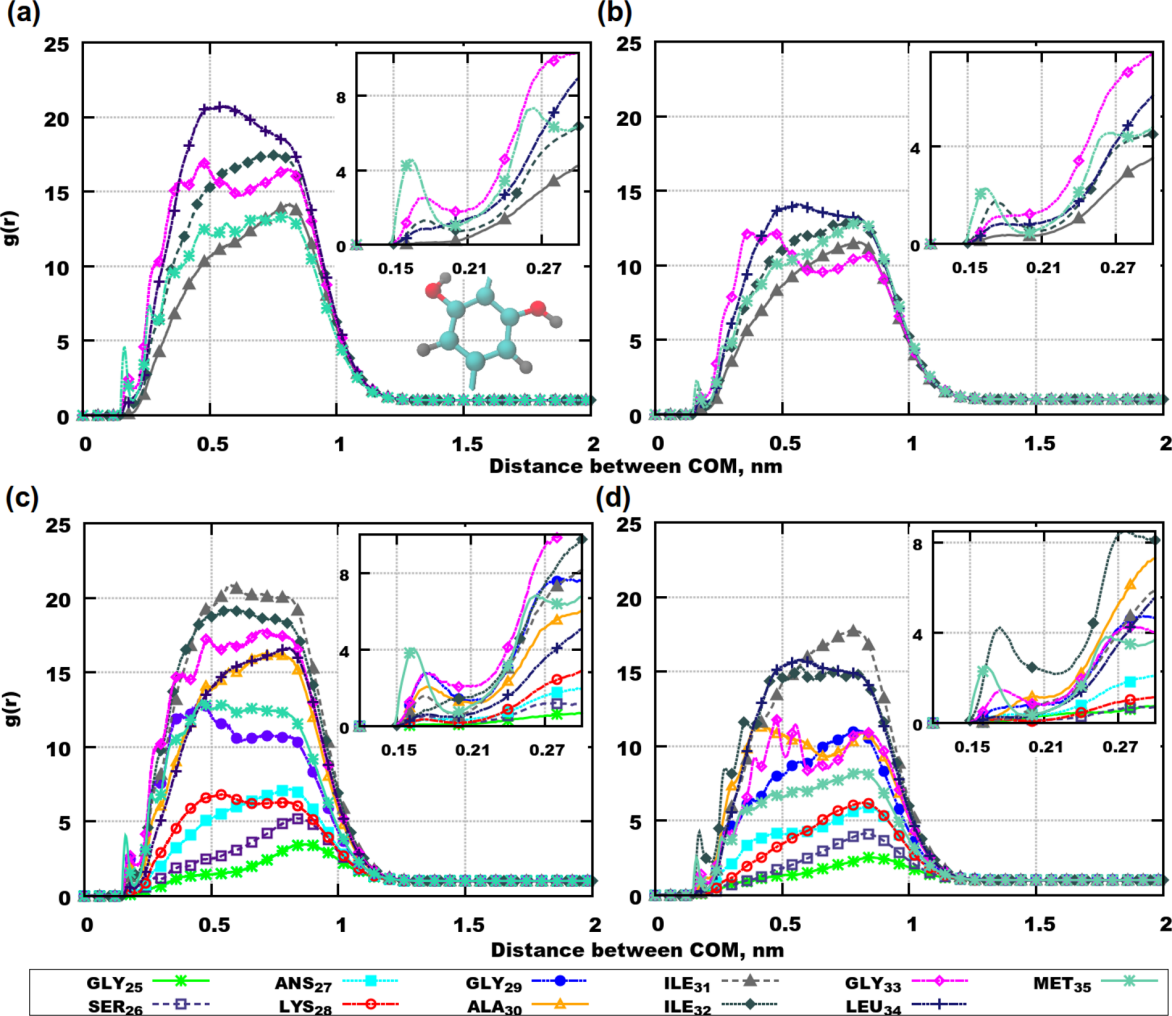
RDFs between centers of mass of amino acid residues and a selected part of CBD molecule (the dihydroxyphenyl ring), computed over 250 ns: (a) 6 Aβ(31–35) and 6 CBD; (b) 8 Aβ(31–35) and 8 CBD; (c) 6 Aβ(25–35) and 6 CBD; (d) 8 Aβ(25–35) and 8 CBD. The dihydroxyphenyl ring is shown in (a), where the gray color denotes hydrogens, carbons are in cyan color, and oxygens are red. Zoomed insets demonstrate RDFs at short distances.

Since CBD affects the aggregation of Aβ peptides, information about which parts of the molecules are associating would be useful for understanding how such interactions can possibly affect the toxicity of Aβ(25–35) and Aβ(31–35). RDFs between centers of mass of amino acid residues and selected parts of CBD can answer this question. Figure 4 demonstrates such RDFs between the dihydroxyphenyl ring of CBD and the different amino acid residues. In simulations with Aβ(31–35) *MET*_35_ is the amino acid residue that associates most with the ring in both simulations (Figure 4a,b): a small peak is observed at a distance of 0.15–0.2 nm. Other amino acid residues which can be found at a close distance to CBD are *GLY*_33_ in the system with 6 molecules and *ILE*_31_ in the system with 8 molecules.

In simulations with Aβ(25–35) *MET*_35_ associates with CBD in both systems with 6 and 8 molecules of each compound (see Figure 4c,d). In the system with 8 molecules *ILE*_32_ is the second amino acid residue that can bind to CBD. Two other amino acid residues with values of RDFs above 1 at short distances below 0.2 nm are *GLY*_33_ and *GLY*_29_ but only in the simulation with 6 molecules. In the system with 8 molecules there are no significant peaks at short distances for the other amino acid residues. This can happen due to conformational rearrangements in Aβ(25–35) in a more crowded environment and with the lesser amount of water.

The fact that CBD can bind to *MET*_35_, which is in C-terminus of both Aβ(31–35) and Aβ(25–35), can be considered as one possible way of decreasing the cytotoxicity of both peptides. For instance, F. Misiti et al.^59^ have used two kinds of Aβ(31–35) in experiments with isolated mitochondria from rat brain, where in one of the cases *MET*_35_ was oxidized to methionine sulfoxide. They noted a reduction of toxic and proapoptotic effects of Aβ(31–35) with modified *MET*_35_, compared to the original one. M. E. Clementi et al.^46^ showed that the substitution of *MET*_35_ by norleucine in Aβ(31–35) and Aβ(25–35) inhibited the apoptotic effects of those peptides in experiments with a clonal line of rat pheochromocytoma. S. Gengler et al.^44^ have tested the ability to aggregate using Aβ(25–35) in comparison with Aβ(35–25) (reverse sequence with *GLY* in C-terminus). Their experiments showed that Aβ(35–25) does not aggregate, in contrast to Aβ(25–35). I. Ermilova et al.^56^ have simulated Aβ(25–35) in a phospholipid bilayer environment and observed that *MET*_25_ binds strongest to the membrane. Thus, preventing the binding of *MET*_35_ by using a drug binding to it could be one possible solution for decreasing the cytotoxicity of Aβ(25–35) and Aβ(31–35).

Interactions between other parts of the CBD molecules and the amino acid residues did not result in high values of RDFs at short distances (see Figures S20 and S21 in Supporting Information), probably because those parts of the drug are mainly hydrophobic and only the dihydroxyphenyl ring has two hydroxyl groups that can participate in hydrogen bonding between CBD and the peptides.

Nevertheless, the knowledge about the associations between centers of mass of *MET*_35_ and the dihydroxyphenyl ring of CBD does not tell whether there is a binding between atoms in C-terminus or other atoms in *MET*_35_. In order to confirm the possible binding between atoms of *MET*_35_ and the dihydroxyphenyl ring, RDFs between selected pairs of atoms were calculated (see Figures S22–S24 in Supporting Information). Figure S22 demonstrates associations between hydrogen atom in the dihydroxyphenyl ring and nitrogen atom in *MET*_35_ (the red curve with peaks at 0.3 nm for all simulated systems) which can be classified as a weak hydrogen bonding interaction. The sulfur atom in *MET*_35_ shows the ability to build strong and weak hydrogen bonds with hydrogens binding to carbons as well as with hydroxyl hydrogens in the dihydroxyphenyl ring of CBD (Figure S23). Moreover, hydrogen atoms from the CH_3_-group of *MET*_35_ can form weak hydrogen bonds with oxygens from dihydroxyphenyl ring of the drug molecule (Figure S24).

Additionally, the simulated drug molecules can aggregate with themselves. The CBD molecules demonstrated the strongest association with themselves in systems containing no peptides, while for systems with peptides the aggregation of CBD was most pronounced when 8 molecules of the peptides were present (Figure S25 in Supporting Information).

And last but not least, the role of the amount of water for aggregation of Aβ peptides is of importance according to our simulations. Considering only simulations without CBD, it was found that both Aβ(31–35) and Aβ(25–35) cluster more easily when their concentration in water is higher as seen in Figure 3a,b by comparing the results for 6 and 8 molecules. The addition of CBD changes the situation: Aβ(31–35) aggregates most in the system with 6 molecules of CBD and the peptide, while Aβ(25–35) shows a stronger association at a higher amount of these molecules.

One reason for such a different behavior of peptides can be their discrepancy in size, compared to the size of the CBD molecule, which has a length similar to the length of Aβ(31–35) but shorter than Aβ(25–35). The shortest peptide can probably be separated by the drug molecules due to their similarities in sizes, while for the long Aβ(25–35) the situation may differ: due to its length, it has an ability to wrap around the CBD molecule.

Another cause of such a diverse aggregation is the presence of a hydrophilic region (25–28) in Aβ(25–35), which does not exist in the shorter peptide. With an increasing amount of both CBD and peptide in systems with Aβ(25–35) the amount of the hydrophilic part is increasing as well (it means that there will be more atoms able to participate in hydrogen bonding interactions), while in the simulations with Aβ(31–35) only hydrophobic regions are present. As it was earlier observed by various research groups, for longer Aβ-peptides, the stability of aggregates was dependent on the hydrophobic interactions in the domain (29–42), which is partially present in both peptides, as well as the existence of the β-turn secondary structure in the hydrophilic region (25–28).^69−73^

This hydrophilic region is involved in hydrogen bonding between peptides and water, which is engaging more water molecules than in the case of 6 peptides (the number of water molecules was the same in the simulated systems) and, probably, be a cause for the differences in aggregation of Aβ(25–35) compared to Aβ(31–35). Figures S26 and S27 of Supporting Information show how the number of hydrogen bonds between peptides and water depends on the water content and the presence of CBD. For systems with Aβ(31–35), both with and without CBD, the number of hydrogen bonds is higher in simulations with 8 molecules than in simulations with 6 (Figure S26). However, the differences between the 4 systems are not substantial. In the case of Aβ(25–35) the number of hydrogen bonds increases substantially with increasing concentration of peptides (i.e., decreasing water concentration), and as for Aβ(31–35) the number of hydrogen bonds does not depend on the presence of CBD in the systems (Figure S27).

### Secondary Structures of the Peptides

Protein secondary structure is known to have an impact on the function of the protein. Such a function can be not only vital for the cell but even cytotoxic.^74,75^ Therefore, another way to investigate the effect of CBD on Aβ peptides is to study how the presence of the drug can affect the structures of Aβ(31–35) and Aβ(25–35). This investigation was carried out with the help of the VMD software.^68^

Figure S28 in Supporting Information demonstrates secondary structures for each peptide in the simulation with 6 molecules of Aβ(31–35). Dominating structures are turn and coil and quite few isolated β-bridges, and α- and 3_10_-helixes can be observed. When 6 molecules of CBD are present in the system, the number of isolated β-bridges increases; no helixes can be detected any longer, and extended conformations appear (Figure S29). In simulations with 8 peptides containing no drugs dominating secondary structures are turn, coil, and many isolated β-bridges, and extended conformations can also be observed (Figure S30). In the presence of 8 CBD molecules the number of isolated β-bridges and extended conformations is lower, and instead turn and coil are the dominating secondary structures (Figure S31).

Returning to the RDFs, it is now possible to see correlations between the aggregation of Aβ(31–35) and its secondary structures. When CBD was absent in the system with 6 peptides (most of the structures here were turn and coil), the peptides were aggregating less than when CBD was present (extended conformations and isolated β-bridges are present in larger amounts in the latter case). Then in the case of 8 molecules of Aβ(31–35) the aggregation of peptides was more pronounced when CBD was absent (extended conformations and isolated β-bridges are present in larger amounts) and substantially reduced in the system with the drug (most of the structures here were turn and coil).

Secondary structures of 6 Aβ(25–35) without CBD in the system can be seen in Figure S32 of Supporting Information. Turn and coil are dominating structures, but a large number of extended conformations, isolated β-bridges, and 3_10_-helixes can also be observed. The less represented structure is α-helix. In the presence of 6 CBD molecules (Figure S33) the number of extended conformations is smaller, as is the number of 3_10_-helixes. There are no α-helixes observed in any of the peptides. Turn, coil, and isolated β-bridge are the dominating secondary structures. In the system with 8 Aβ(25–35) without any CBD (Figure S34) the most represented secondary structures are extended conformation, turn, and coil, which can be seen in every peptide. Isolated β-bridges and α-helixes can be observed as well but in smaller amounts. With the addition of 8 CBD molecules (Figure S35) to the system with 8 Aβ(25–35) the number of extended conformations is decreasing and a lesser amount of α-helixes is detected, but 3_10_-helixes and isolated β-bridges are getting more pronounced.

Then these results for Aβ(25–35) can be connected to the results from the RDF analysis, since the aggregation and high values of RDFs at shorter distances can be related to the presence of certain peptide secondary structures in the modeled systems. For instance, in simulations with 8 molecules, extended conformations can be detected in large amounts (during the whole simulation time) in almost every single peptide, regardless if CBD is present or absent. In all those simulations a high aggregation of Aβ(25–35) is observed. In systems with 6 molecules a larger number of extended conformations in different combinations with isolated β-bridges appear when CBD is absent, while in the presence of CBD these structures exist in smaller amounts. For those simulations peptides were aggregating strongest in the absence of the drug and much less in its presence. Thus, a high number of extended conformations and β-bridges is correlated with a stronger aggregation of Aβ(25–35).

Indeed, secondary structures in combinations with RDFs give some idea about how the aggregation of Aβ peptides occurs or, better to say, what conformations should be dominant in order to observe such a phenomenon. Extended conformations were seen in amyloid fibrils and precipitates in experimental studies.^76−79^ The presence of β-turn structures in the hydrophilic domain (25–28) and the hydrophobic domain (29–35) was pointed out as the essential “conditions” for stable aggregation of Aβ-peptides by C. J. Pike et al.^69^ and many others.^71−73^ Thus, results from those experimental studies seem to have some agreement with our findings.

**Figure 5:**
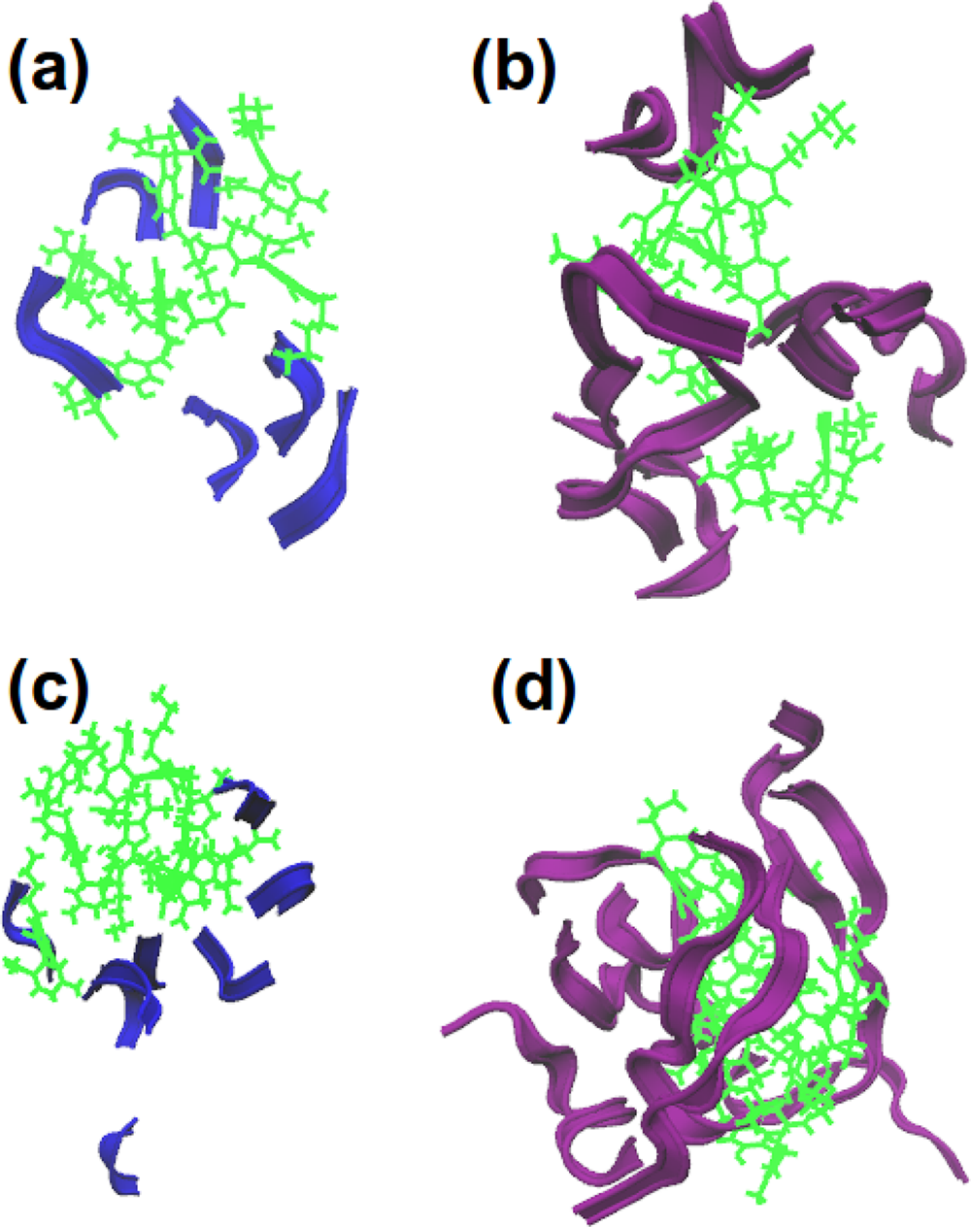
Screenshots of the final simulation frame: (a) 6 Aβ(31–35) and 6 CBD; (b) 6 Aβ(25–35) and 6 CBD; (c) 8 Aβ(31–35) and 8 CBD; (d) 8 Aβ(25–35) and 8 CBD. Here, green molecules are CBD, purple ribbons are Aβ(25–35), and blue ribbons are Aβ(31–35).

Nevertheless, a discussion about how the secondary structure may give a rise to pharmacological effects shall be completed by taking a look at the systems’ screenshots. Figure 5 demonstrates screenshots of the final frames for systems containing CBD and peptides. All images have something in common: the drug molecules are clustered, and the peptides surround these clusters. Considering the mechanisms of actions of CBD, it would be reasonable to think about two possible ways: the first one is a separation of peptides and the second one is their ”adsorption” on the surface of CBD clusters. Which of the mechanisms is the most effective against cytotoxicity cannot be concluded from simulations, since any toxic effect shall be evaluated on living neurons.

### Potential Mean Force Profiles and Free Energies from Well-Tempered Metadynamics

**Figure 6:**
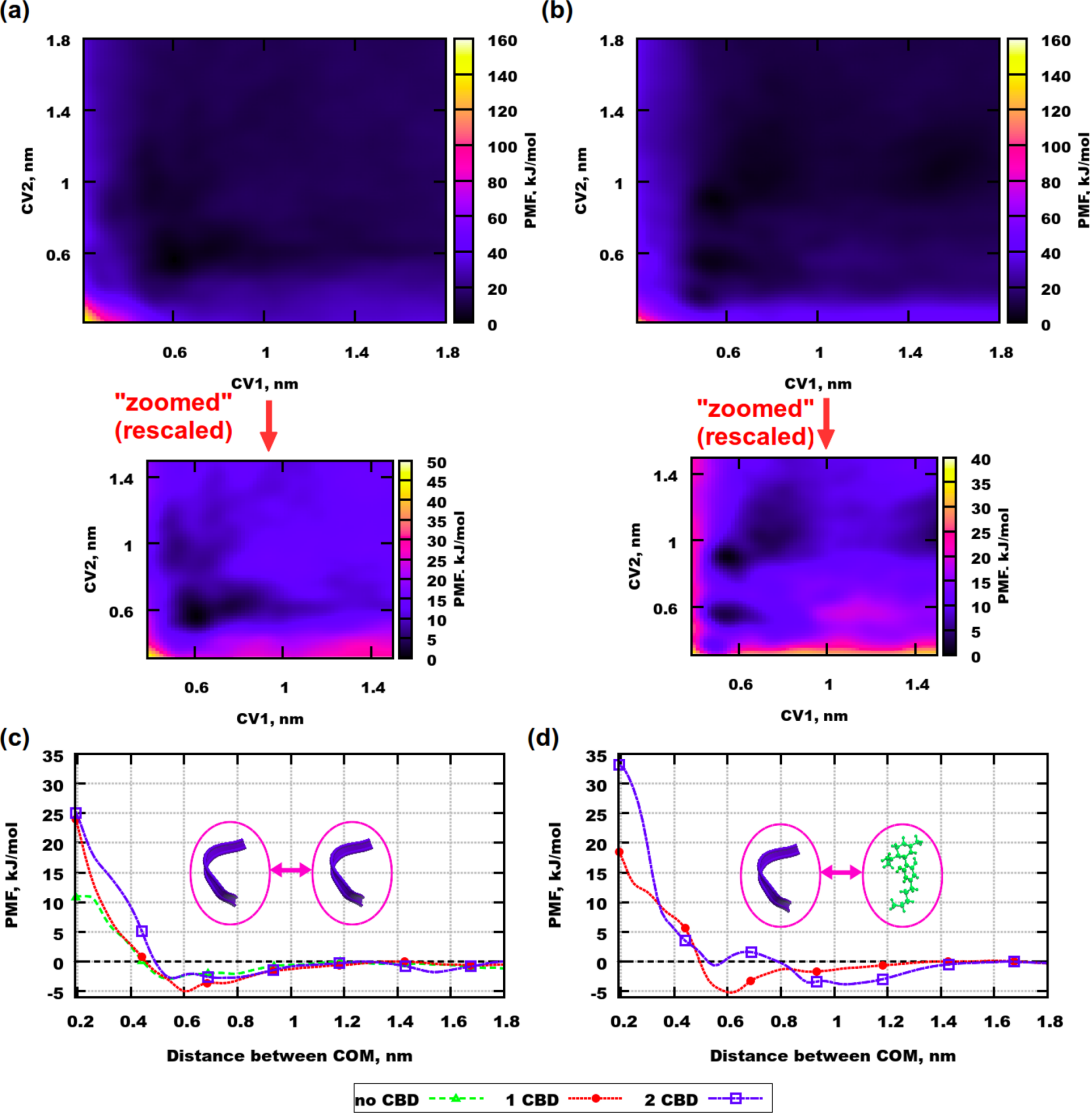
PMF profiles for well-tempered metadynamics simulations with Aβ(31–35). (a) Simulation with 2 peptides and 1 CBD molecule. (b) Simulation with 2 peptides and 2 CBD molecules. (c) Green curve is for one-dimensional simulation containing no CBD molecules. Red and blue curves are integrated profiles for peptides from simulations containing 1 and 2 CBD molecules, respectively. (d) Red and blue curves are integrated profiles for peptides and CBD molecules from simulations containing 1 and 2 CBD molecules, respectively. CV1 and CV2 are defined in Figure 9.

The resulting potential mean force (PMF) profiles for all simulations with Aβ(31–35) are shown in Figure 6. Since the length of a stretched peptide is about 1.5 nm, distances longer than 1.8 nm were not considered for the analysis. In Figure 6a it can be seen that the area of the lowest energy is situated at a distance between the peptides (CV1) of 0.5–0.8 nm and at a distance between peptide-1 and CBD (CV2) of 0.3–0.7 nm. This implies that two Aβ(31–35) can be situated close to each other regardless of the presence of the CBD molecule. At the same time dark areas of the same color but weaker intensity can be observed at the same distance for CV1 but at a longer distance for CV2, which means that two peptides can be located close to each other even if 1 CBD molecule is further apart, but with a lower probability. Figure 6b shows the profile for the simulation with 2 peptides and 2 CBD molecules. Two clear points of minima can be determined here: one point for a distance between the peptides of 0.4–0.6 nm and the same distance between one of the peptides and one CBD molecule. Another point is for the same distance between the peptides and a distance between one of the peptides and one of the CBD molecules of 0.8–0.9 nm. Such an existence of 2 well distinguished minima indicates that aggregation of two peptides is equally probable when 1 CBD molecule is situated at one of those defined distances. When the CBD molecule is at the distance of 0.8–1.2 nm, two dark areas with lower intensity can be observed when the distances between the peptides are 0.7–0.8 nm and 1.4–1.5 nm, which means that peptides can be separated in the presence of CBD molecules. Since a comparison of the free energy maps shows that several points of minima at various distances between the molecules can be observed at the presence of 2 CBD molecules, it can be concluded that the drug can inhibit aggregation.

However, regardless of these results, a final conclusion about aggregation can be made only if the results are compared with a simulation of a corresponding system containing no CBD. Figure 6c demonstrates PMF profiles for 3 systems with Aβ(31–35), including one without CBD (green line). Red and blue profiles were obtained from an integration of the free-energy maps computed for systems with 1 and 2 CBD molecules, respectively. For the simulation without any drug the curve looks rather flat after a distance of 0.45 nm, compared to the two other curves. In the system with 1 CBD molecule the peptides cluster more easily than in the simulation with 2 CBD molecules. This implies that Aβ(31–35) has a low tendency to aggregate even without CBD, but if CBD is present in the system, then a higher amount is favorable for separating the peptides.

Then the question arises about the affinity of CBD to Aβ(31–35). Figure 6d presents integrated PMF profiles for interactions between the peptide and CBD. It is clear that in the system with 1 CBD molecule Aβ(31–35) has a stronger affinity to the CBD molecule, while in the system with 2 CBD molecules the global minimum is observed at a longer distance between the peptide and CBD (at about 1.1 nm). This implies that CBD prefers to be located relatively far away from Aβ(31–35) when 2 CBD molecules are present. Thus, it is possible that the second CBD molecule could be involved in the inhibition of peptide aggregation.

**Figure 7:**
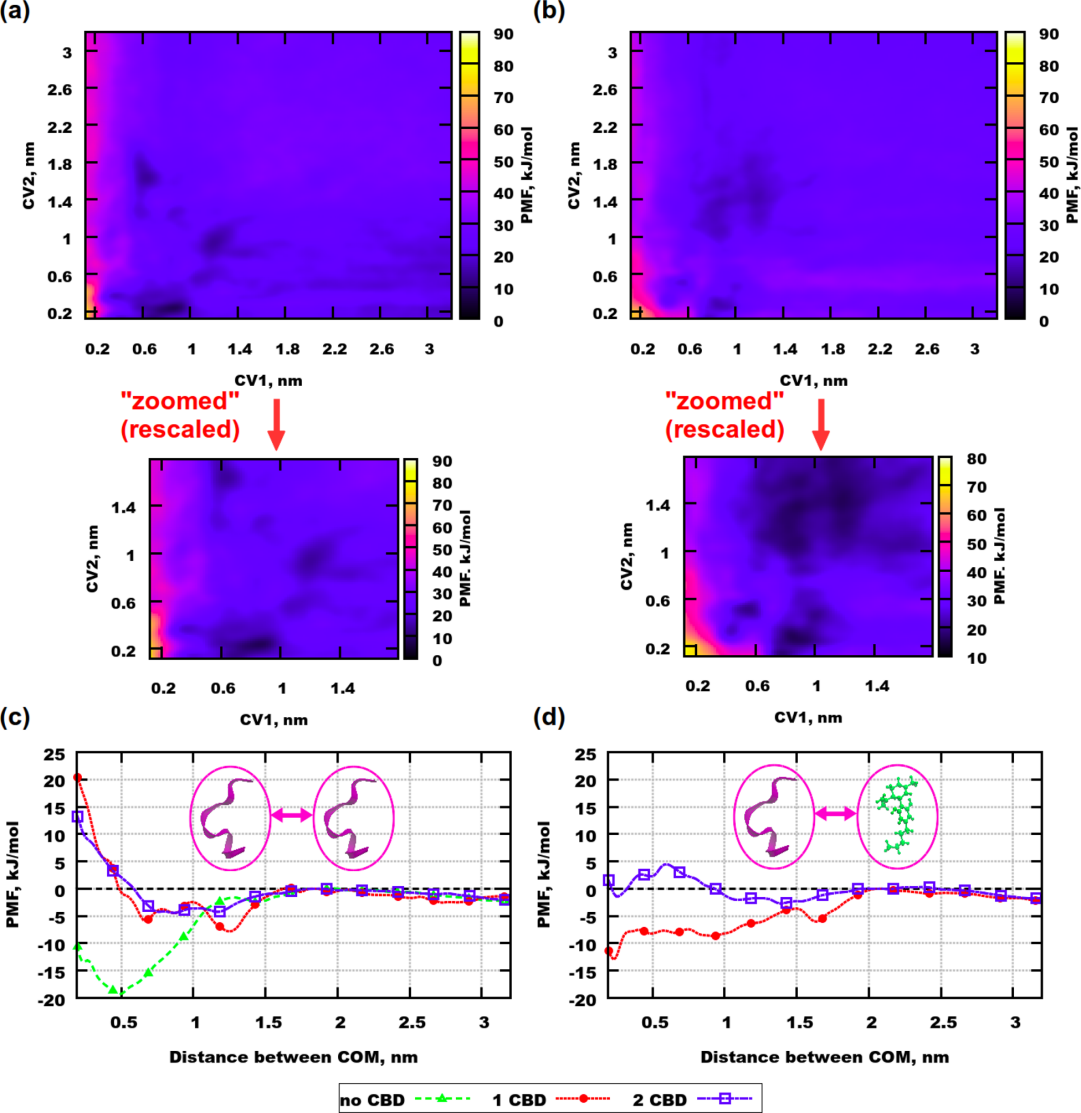
PMF profiles for well-tempered metadynamics simulations with Aβ(25–35). (a) Simulation with 2 peptides and 1 CBD molecule. (b) Simulation with 2 peptides and 2 CBD molecules. (c) Green curve is for one-dimensional simulation containing no CBD molecules. Red and blue curves are integrated profiles for peptides from simulations containing 1 and 2 CBD molecules, respectively. (d) Red and blue curves are integrated profiles for peptides and CBD molecules from simulations containing 1 and 2 CBD molecules, respectively.

For the larger peptide Aβ(25–35) the situation with aggregation is different, compared to Aβ(31–35). The stretched Aβ(25–35) is much longer than Aβ(31–35) (approximately 3 nm). Therefore, the distances considered for calculations are a bit longer. Figure 7a demonstrates the free energy map for the system containing 2 peptides and 1 CBD molecule. The global minima is located at a distance between the peptides of 0.7–0.8 nm when the CBD molecule is situated at about 0.2 nm from one of the peptides. At this distance between the peptides and a distance between CBD and one of the peptides of 1.7 nm a local minima can be observed, which means that peptides can aggregate even if the CBD molecule is relatively far away. A local minima with a similar intensity can be seen even at a distance between the peptides of 1.2 nm, when the distance between one of the peptides and the CBD is about 1 nm. This implies that the peptides can be separated when the CBD molecule is far away. However, since the global minimum is at a distance that is much smaller than half the length of the stretched peptide, one can conclude that aggregation is more dominant in this mixture, but barriers from bound to unbound states are not big. In the simulation with 2 CBD molecules, aggregation does not dominate anymore (Figure 7b). The barrier between bound and unbound states is smaller than in the one-dimensional simulation. This implies that less energy is needed for separating the peptides. There are several areas with minima in PMF. Those areas appear at different distances between the peptides as well as between CBD and one of the peptides. The free energy landscape appears more homogeneous and flat, compared to the one with a single molecule of the drug.

From the PMF it can be concluded that aggregation of the peptides does not dominate in the system. As in the previous case of Aβ(31–35) it is reasonable to consider the system without any CBD in order to understand if the drug can give any ”benefits” in terms of inhibition of peptide aggregation. Figure 7c demonstrates that without CBD Aβ(25–35) aggregation is more probable since the global free energy minimum is deeper and placed at a shorter distance of 0.5 nm, compared to the systems with 1 and 2 CBD molecules. Moreover, the curve for the system with 2 CBD is placed higher than the curve for the system with 1 CBD, which implies that the higher concentration of the drug inhibits the aggregation better than the lower one. Nevertheless, Figure 7d shows that there is a higher affinity of CBD to Aβ(25–35) in the system with only one drug molecule, compared to the system with two CBD molecules. This behavior is similar to that observed for the systems with Aβ(31–35). Then we can also speculate that the second CBD molecule plays a big role in the inhibition of the peptide aggregation process.

Observing energetically favorable distances is a good approach for understanding if molecules are binding to each other, but the final conclusion can be made only after an integration of the PMF profiles. Table 1 presents results from such calculations. Binding free energies were calculated according to eq 1:
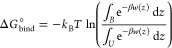
1Here *k*_B_ is the Bolztmann constant, *T* is the temperature during the simulation, β = 1/(*k*_B_*T*), *z* is the value of a CV, and *w*(*z*) is the value of the PMF. The integral, denoted by the letter *B*, stands for the bound state (when two molecules are close enough to each other so that binding can occur), and the letter *U* stands for the unbound state (when two molecules are far away from each other and no binding between them can happen).

**Table 1:** Free Energies

	binding free energy (kJ/mol)	
system	Aβ ↔ Aβ	Aβ ↔ CBD
2 Aβ(31–35)	0.332	
2 Aβ(31–35) + 1 CBD	–1.343	–1.050
2 Aβ(31–35) + 2 CBD	–0.318	–0.453
2 Aβ(25–35)	–12.911	
2 Aβ(25–35) + 1 CBD	–2.348	–6.302
2 Aβ(25–35) + 2 CBD	–0.977	0.247

In Table 1 values of binding free energies are shown. They give insight into how likely two molecules are bound to each other. In the system with only 2 Aβ(31–35) the binding free energy is higher than in the systems with CBD. The positive value of binding free energy implies that two molecules are highly unlikely to bind. In the simulations with the drug the two peptides have the lowest binding free energy when only 1 CBD is present in the system. At the same time this CBD molecule has a higher affinity to a peptide than in the system containing 2 CBD molecules. In the case of Aβ(25–35) the lowest binding free energy between the peptides was observed in simulations without any drug and the highest one was for the system with 2 CBD molecules. This implies that at a higher content of CBD Aβ(25–35) is less likely to aggregate. Peptide and CBD have the lowest binding free energy in the system with 1 drug molecule, while in the simulation with 2 CBD molecules the value of free energy is positive, which implies that the two molecules are unlikely to bind to each other.

These diverse aggregation properties of Aβ(31–35) and Aβ(25–35) in aqueous mixtures without drugs were observed in experiments by C. J. Pike et al.^70^ Moreover, according to their earlier studies,^69^ the ability of Aβ(25–35) to build aggregates is strongly related to the presence of both hydrophilic (25–28) and hydrophobic (29–35) domains, where the (25–28)-region of the sequence is “responsible” for the stability of the aggregates due to its β-turn secondary structure.^71−73^ However, in the presented free energy calculations secondary structures were not taken into account during calculations.

The information about the quality of sampling and convergence of presented well-tempered metadynamics simulations can be found in the section 2 of Supporting Information.

### Possible Mechanisms of Actions

The cytotoxicity of Aβ peptides is a complex phenomenon that can depend on many different factors such as their secondary structure, their tendency to aggregate, their ability to bind, and even the amount of water and other chemical compounds in living organisms.

Aβ(31–35) and Aβ(25–35) are already known to differ in aggregative properties and in neurotoxic mechanisms from various experiments, regardless that they are sharing the same part of the sequence (31–35).

**Figure 8:**
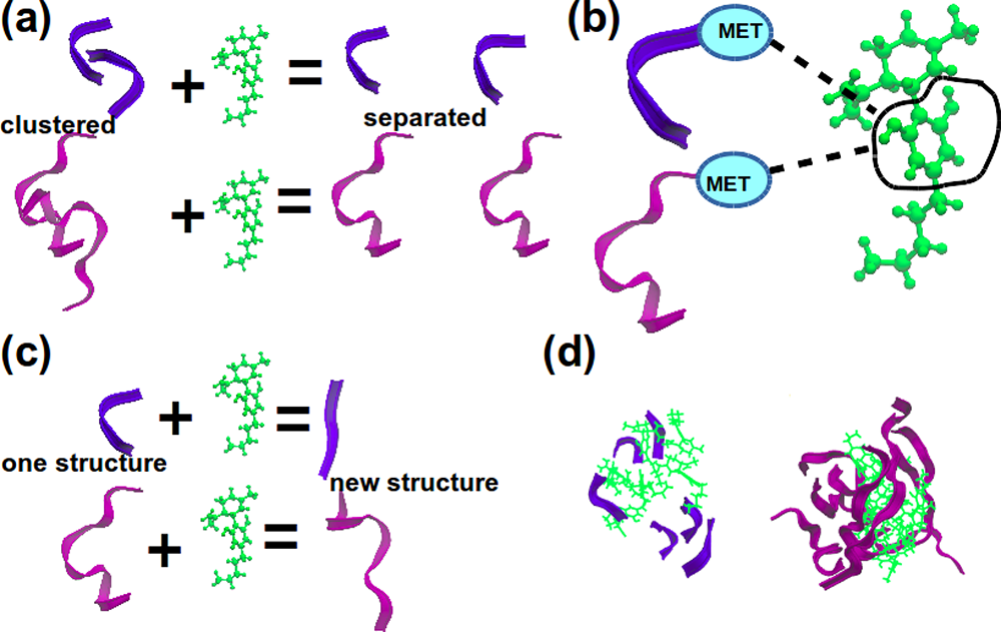
Illustration of possible mechanisms of actions of CBD on Aβ peptides: (a) inhibition of peptide aggregation; (b) binding of the dihydroxyphenyl ring to *MET*_35_ (the amino acid residue is accented by an ellipse); (c) change of the secondary structure; (d) clustering of CBD with adsorption of peptides around the clusters. Here colors are the following: “blue” ribbons are Aβ(31–35), “purple” ribbons are Aβ(25–35), and “green” molecule is CBD. Sizes of molecules were rescaled for clarity.

Results from our MD simulations showed that there are 4 possible interrelated mechanisms of actions of CBD on the investigated peptides (Figure 8). Therefore, it is important to say that one mechanism can be the cause or/and the consequence of another.

The first one is an inhibition of aggregation of the peptides (Figure 8a). Aβ(25–35) exhibited different aggregation properties comparing to Aβ(31–35) in mixtures containing no drug, which was in agreement with experimental results.^69,70^ However, in the presence of the CBD molecules, depending on their concentration in water, the peptides showed sundry behavior. This shall give rise to future investigations of various CBD:peptide:water ratios in order to find the optimal concentration of the drug. Due to the big differences in results from classical MD simulations, it would also be worth considering possible nanotoxic effects of both CBD and peptides.^80,81^

The second one is binding of the dihydroxyphenyl ring in CBD to *MET*_35_ (Figure 8b). Since this amino acid residue has been seen as a possible cause of cytotoxicity,^46,48,59,60,82^ CBD can certainly be considered as a possible drug.

The third one is a CBD induced alteration of the secondary structure of the Aβ peptides (Figure 8c). It was discovered in a number of experimental studies that certain secondary structures were dominating in the case of aggregation and therefore seem to promote or to be required for stable aggregation.^69−73^ Since a protein can “change” its function by a change of its secondary structure, one can speculate that our observation that CBD gives rise to lesser amounts of extended conformations in combinations with isolated β-bridges of the peptides may be related to reduced toxicity and aggregation.

The fourth possible mechanism of action is the adsorption of peptides on CBD clusters (Figure 8d). In MD simulations high values of intermolecular RDFs were observed at shorter distances in the presence of the CBD molecules, but the screenshots showed that aggregates are built around the drug clusters. Aβ(31–35) has a similar length as the CBD molecule, while Aβ(25–35) has a double length of the drug. At a higher concentration of both molecules the separation of the shortest peptide can be observed due to the drug cluster in between, while for Aβ(25–35) an aggregation of peptides on such a cluster can appear as the aggregation of the peptides (since their centers of mass are close to each other). Thus, if peptides would associate with each other on such clusters, perhaps they would not aggregate on membrane surfaces.

Additionally we can conclude that the amount of water has a strong effect on the tendency of the peptides to aggregate in the absence of CBD: both were aggregating more easily in systems with 8 molecules than in simulations with 6. The number of hydrogen bonds between water and peptide molecules was higher in systems with Aβ(25–35) than with Aβ(31–35). A growing concentration of peptides significantly increases the number of hydrogen bonds for the longer peptide, while in the case of the shorter one the number of hydrogen bonds was not substantially affected by concentrations of peptides and water.

Well-tempered metadynamics simulations on a microsecond time-scale provide information about the energetics of the different molecular interactions, which in turn can partially explain the observations made from the classical MD simulations. Aβ(31–35) does not aggregate in the absence of CBD. In the mixture with 1 CBD molecule it shows the strongest tendency to aggregate. Also the affinity of Aβ(31–35) toward CBD is higher than in the mixture with 2 CBD molecules. The presence of the second CBD molecule affects the energetics of the peptide–peptide and peptide–CBD interactions by suppressing the aggregation of the peptides. However, the cytotoxicity of Aβ(31–35) is not related to its aggregation. As it was shown in several experimental works,^46,70^ Aβ(31–35) did not aggregate in aqueous solutions and its toxicity was not correlated with its ability to aggregate.

The aggregation of Aβ(25–35) is gradually inhibited at higher amounts of CBD. The affinity of Aβ(25–35) toward the selected CBD molecule is lower at the highest amount of the drug in the system. Since the toxicity of Aβ(25–35) is strongly related to its aggregation, the inhibition of this process by CBD can probably suppress the cytotoxicity of Aβ(25–35).

## Conclusions

In this work, we have investigated possible mechanisms of actions of CBD against cytotoxicity of Aβ(31–35) and Aβ(25–35) applying advanced *in silico* methods such as classical MD and well-tempered metadynamics simulations. We propose four possible interrelated mechanisms of actions of CBD that could inhibit the death of neurons out of knowledge about the cytotoxic mechanisms determined by a number of experimental studies and the present results from molecular modeling.

For the possible inhibition of cytotoxicity in systems with Aβ(31–35) CBD could bind to *MET*_35_, alter the peptide secondary structure, and adsorb Aβ(31–35) on CBD clusters. In the case of Aβ(25–35) the suppression of peptide aggregation can be an additional action of CBD against the Aβ cytotoxicity, while for Aβ(31–35) peptide aggregation might not be relevant at all. All those mechanisms are interdependent as well: inhibited aggregation can be due to altered secondary structures. The adsorption on CBD clusters can occur through binding to *MET*_35_.

Moreover, the amount of water in the simulated mixtures also plays a role in the peptide aggregation process as well as in the interactions between peptides and CBD molecules. Both peptides show a higher tendency to aggregate in the absence of the drug in the systems with the lower water content. The presence of CBD can, however, promote peptide aggregation around CBD clusters in the water rich systems. Additionally, the number of hydrogen bonds detected between peptides and water was higher in the systems with Aβ(25–35) than with Aβ(31–35). The presence of the drug molecules did not affect the number of hydrogen bonds in the simulated systems.

From computational results we can say that CBD shall be considered for further *in vivo* and *in vitro* studies as a possible drug against the neurodegenerative diseases. As future *in silico* experiments, MD simulations of mixtures with various ratios of CBD and peptides, including Aβ peptides with longer chains, should be considered. Moreover, combining computational and experimental studies would help to find optimal concentrations of the drug.

## Methods

### Classical MD Simulations

Before the setting up of MD simulations, the model for the CBD molecule was derived using the same approach as for the general Amber force field (GAFF).^83^ Twenty random conformations were utilized for the calculations of partial atomic charges. According to the specification of GAFF, the partial atomic charges were computed on the optimized molecular geometries by ab initio calculations employing the Hartree–Fock method with the 6-31G(d) basis set and the restrained electrostatic potential (RESP)^84^ fitting method. Gaussian 16^85^ was used for those computations.

After the derivation of the CBD model, starting configurations for MD were set up. Compositions of simulated systems are shown in Table 2. First, systems 1–4 were created in the following way: in order to avoid clustering, the peptides were placed in empty boxes with an artificial van der Waals radius equal to 0.5 nm. Then in configurations with Aβ(25–35) chlorine counterions were added to compensate the positive charge of the peptide (one ion per one Aβ(25–35)). Aβ(31–35) had a total charge equal to 0 so no counterions were inserted in simulations with this peptide. Systems 5 and 6 with CBD were built applying the same van der Waals distance of 0.5 nm around every CBD molecule. Systems 7–10 were created from the starting configurations 1–4 by adding the CBD molecules using the van der Waals radii of 0.5 nm around each molecule. Chlorine counterions were added in every simulation box containing Aβ(25–35) for compensating the positive charge. After the insertion of larger molecules and ions the water molecules were randomly placed in every system.

**Table 2:** Molecular Compositions of Simulated Systems

system	number of Na ions	number of water molecules
6 Aβ(25–35)	6	10000
6 Aβ(31–35)	0	10000
8 Aβ(25–35)	8	10000
6 Aβ(31–35)	0	10000
6 CBD	0	10000
8 CBD	0	10000
6 Aβ(25–35) + 6 CBD	6	10000
6 Aβ(31–35) + 6 CBD	0	10000
8 Aβ(25–35) + 8 CBD	8	10000
8 Aβ(31–35) + 8 CBD	0	10000

The models for the Aβ peptides were taken from amber99sb-ildn FF^86^ at neutral pH. All systems were simulated for 250 ns in the *NPT* ensemble using the isotropic pressure coupling scheme, where the equilibration was 10 ns long. The temperature of 310 K was regulated by a Velocity Rescale thermostat^87^ with a coupling constant of 0.5 ps. The pressure of 1 atm was retained by the Berendsen barostat^88^ with a coupling constant of 10 ps and a compressibility of 0.000 045 bar^–1^. All bonds were constrained by using the LINCS^89,90^ algorithm with 12 iterations. The time step was 2 fs and the cutoff value was 0.9 nm. The integrator was the leapfrog algorithm.^91^ The MD software was GROMACS 2019.^92,93^ Final dimensions of simulation boxes after equilibration with classical MD simulations are shown in Table S1 of Supporting Information.

### Well-Tempered Metadynamics Simulations

Well-tempered metadynamics simulations were carried out for 6 systems.

**Figure 9:**
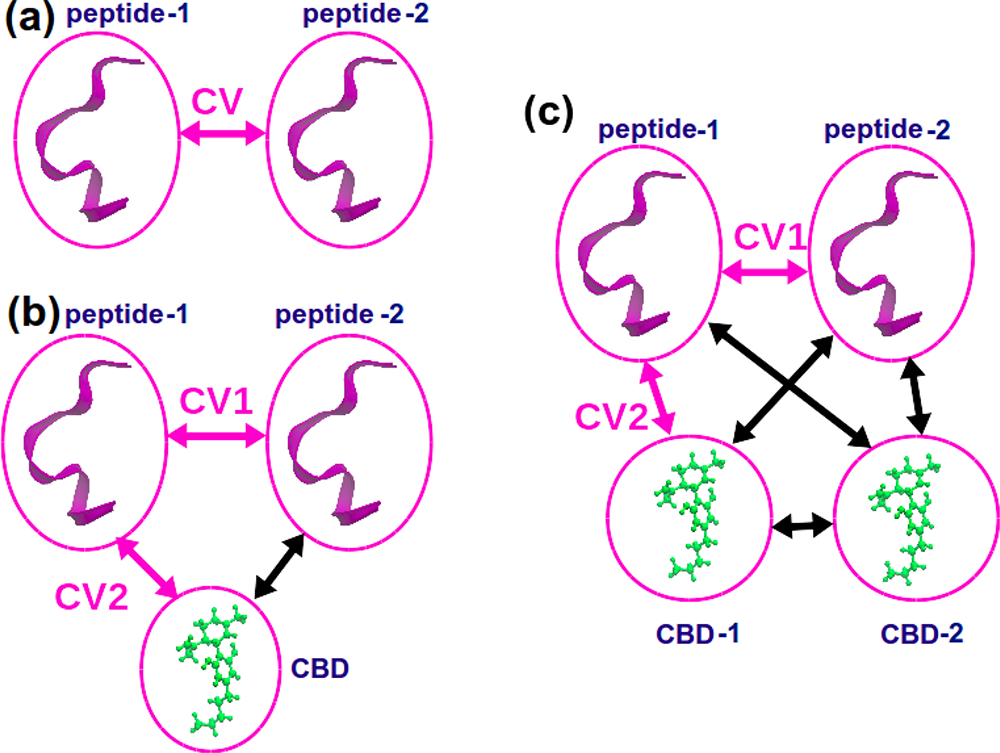
Collective variables used in well-tempered metadynaimics simulations. (a) 1D simulations for systems containing peptides in water. The distance between centers of mass of peptides is a CV. (b) 2D simulations for systems containing 2 peptides and 1 CBD molecule. CV1 is the distance between centers of mass of peptides. CV2 is the distance between centers of mass of a peptide and a CBD molecule. The black arrow shows the distance which was not taken into account. (c) 2D simulations for systems containing 2 peptides and 2 CBD molecules. CV1 is the distance between centers of mass of peptides. CV2 is the distance between centers of mass of a peptide and a CBD molecule. The black arrows show the distances which were not taken into account.

The first 2 systems contained only 2 peptides each (one with 2 Aβ(25–35) and another one with 2 Aβ(31–35)), where the collective variable (CV) was the distance between the center of mass of the peptides (Figure 9a). These simulations were 7 μs long.

The second 2 systems contained 2 peptides each and 1 CBD molecule each. Two collective variables were given by the distance between the center of mass of the peptides (CV1) and the distance between the center of mass of the CBD molecule and one of the peptides (CV2) (Figure 9b). During these simulations the distance between the center of mass of the second peptide and the CBD molecule was not taken into account. These simulations were 10 μs long.

The final 2 systems contained 2 peptides and 2 CBD molecules each. Two collective variables were given by the distance between the center of mass of the peptides (CV1) and the distance between the center of mass of one of the CBD molecules and one of the peptides (CV2) (Figure 9c). During these simulations the following distances were not considered: the distance between the center of mass of the first peptide and the second CBD molecule, the distance between the center of mass of the second peptide and the first CBD molecule, the distance between the center of mass of the second peptide and the second CBD molecule, and the distance between the center of mass of the two CBD molecules. These simulations were 12 μs long.

Starting configurations were created in the following way: two simulation boxes containing peptides and CBD molecules were selected from classical MD simulations. One box was with 6 Aβ(25–35) and 6 CBD molecules, and another box was with 6 Aβ(31–35) and 6 CBD molecules. For the well-tempered metadynamics simulations with 2 peptides in the selected 2 boxes only 2 peptides and water molecules (and counterions for Aβ(25–35)) were kept. Then the 2 resulting boxes were equilibrated for 10 ns in the *NPT* ensemble at a pressure of 1 atm and a temperature of 310 K. Final frames were used for well-tempered metadynamics simulations. All other starting configurations for simulations containing 2 peptides and 1–2 CBD molecules were created using a similar approach from the same frames, extracted from classical MD simulations. The number of water molecules was kept the same as in the classical MD simulations. Final dimensions of the simulation boxes after equilibration with classical MD simulations are demonstrated in Table S2 of Supporting Information.

After preparation of the starting configurations, parameters for well-tempered metadynamics simulations were set. The Gaussian functions of the height 1.2 kJ/mol and the width for every collective variable of 0.05 nm (the parameter σ) were deposited every 500 steps (i.e., the parameter PACE was 500). The bias factor, γ, was equal to 50.0. The simulations were carried out in the *NVT* ensemble by the Velocity Rescale^87^ thermostat at a temperature of 310 K using GROMACS 2019.4^92,93^ as an MD engine with PLUMED 2.5.4^94^ for well-tempered metadynamics. The employed force fields were the same as the ones used for the classical MD simulations. The time step was 2 fs, the integrator was the leapfrog algorithm,^91^ and the cutoff value was 0.9 nm.

## Supporting Information Available

Additional results of analysis of simulations (PDF)
