# Supplementary material for: Component of Cannabis, Cannabidiol, as a Possible Drug against the Cytotoxicity of Aβ(31–35) and Aβ(25–35) Peptides: An Investigation by Molecular Dynamics and Well-Tempered Metadynamics Simulations
Source: ACS Chem Neurosci. 2021 Feb 5;12(4):660–74. doi: 10.1021/acschemneuro.0c00692 (PMC8023578; doi:10.1021/acschemneuro.0c00692)
Supplement: Supplementary file 1 — cn0c00692_si_001.pdf [file cn0c00692_si_001.pdf]

Component of cannabis, cannabidiol, as a possible drug against the cytotoxicity of A$\beta(31-35)$ and A$\beta(25-35)$ peptides: an investigation by molecular dynamics and well-tempered metadynamics simulations

Supporting Information

Wojciech Chrobak, Dawid Wojciech Pacut,
Fredrik Blomgren, Alexander Rodin, Jan Swenson, Inna Ermilova*

Department of Physics,
Chalmers University of Technology, SE 412 96, Gothenburg, Sweden

E-mail: inna.ermilova@chalmers.se; ina.ermilova@gmail.com

Content

1. Classical MD

• 1.1. Final dimensions of simulation boxes and molar concentrations of compounds: Table S1.

• 1.2. RDFs, radius of gyration, contact maps and hydrogen bonds.
▷ RDFs between center of mass of peptides on different time intervals: Figures S1-S2.
▷ Contact maps: Figures S3-S10.
▷ RDFs between center of mass of peptides and CBD: Figure S11.
▷ Radius of gyration of peptides: Figures S12-S19.
▷ RDFs between centers of mass of amino acid residues and selected parts of CBD molecule: Figures S20-S21.
▷ RDFs between selected atoms in CBD and MET_{35}: Figures S22-S24.
▷ RDFs between center of mass of CBD molecules: Figure S25.
▷ Hydrogen bonds formed between peptides and water: Figures S26-S27.

• 1.4. Secondary structures: Figures S28-S35.

2. Well-tempered metadynamics

• 2.1. Final dimensions of simulation boxes and molar concentrations of compounds: Table S2.

• 2.2. Discussion about the quality of sampling and convergence.

• 2.3. Quality of sampling: collective variables, secondary structures of peptides etc.: Figures S36-S43.

• 2.4. Convergence studies: Figures S44-S47.

• 2.5. Printouts of HILLS-files: Figures S48-S50.

1 Classical MD

1.1 Final dimensions of simulation boxes

Table S1: Dimensions of simulation boxes (in nm) after the equilibration (classical MD) and concentrations (c) of components (in mol/m^3).

System	x	y	z	c(Aβ)	c(CBD)	c(Na)
6 Aβ(31 − 35)	6.77	6.77	6.77	32.11	none	none
8 Aβ(31 − 35)	6.79	6.79	6.79	42.44	none	none
6 Aβ(25 − 35)	6.80	6.80	6.80	31.69	none	31.69
8 Aβ(25 − 35)	6.82	6.82	6.82	41.88	none	41.88
6 CBD	6.77	6.77	6.77	none	32.11	none
8 CBD	6.78	6.78	6.78	none	42.62	none
6 Aβ(31 − 35)+ 6 CBD	6.80	6.80	6.80	31.69	31.69	none
8 Aβ(31 − 35)+ 8 CBD	6.81	6.81	6.81	42.06	42.06	none
6 Aβ(25 − 35)+ 6 CBD	6.83	6.83	6.83	31.27	31.27	31.27
8 Aβ(25 − 35)+ 8 CBD	6.84	6.84	6.84	41.51	41.51	41.51

The following equation was utilized for calculations of molar concentrations of compounds:

$$c = \frac{N}{N_A V} \tag{S1}$$

Here N is the number of molecules of a certain compound, $N_A = 6.02214076 \cdot 10^{23}\ mol^{-1}$ is the Avogadro's number, V is the volume of the simulation box.

1.2 RDFs, radius of gyration and contact maps

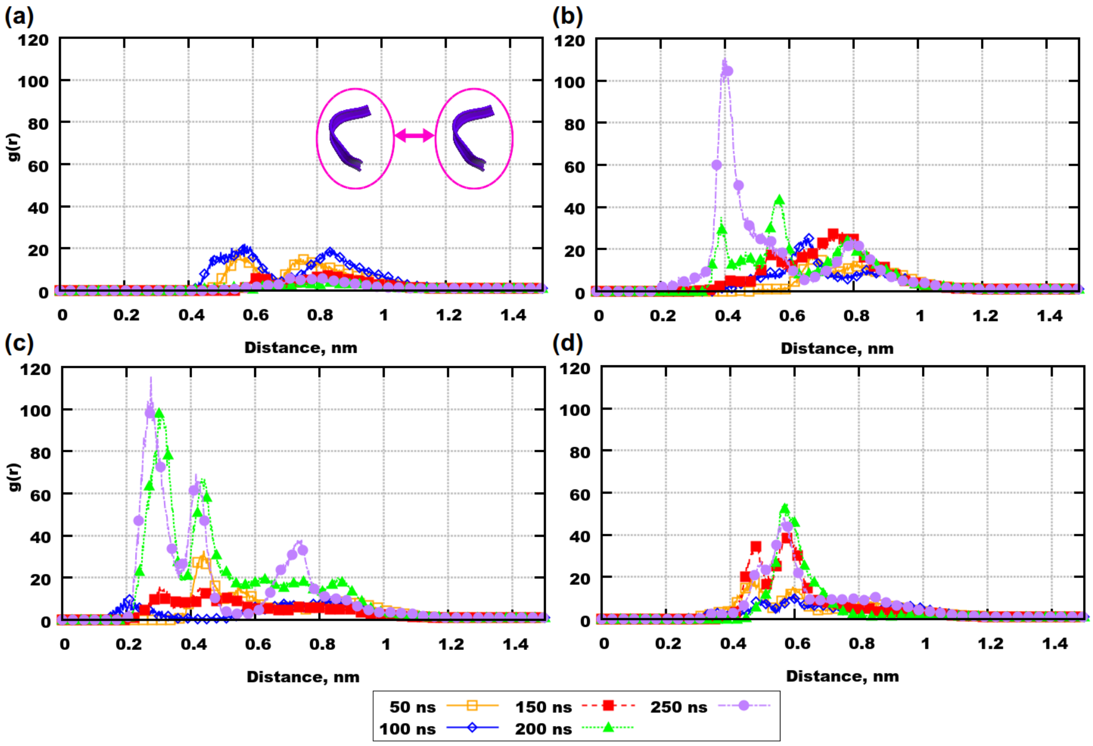

Figure S1: RDFs between centers of mass of peptides for different time intervals. (a) 6 A$\beta(31-35)$ (b) 6 A$\beta(31-35)$ and 6 CBD (c) 8 A$\beta(31-35)$ (d) 8 A$\beta(31-35)$ and 8 CBD.

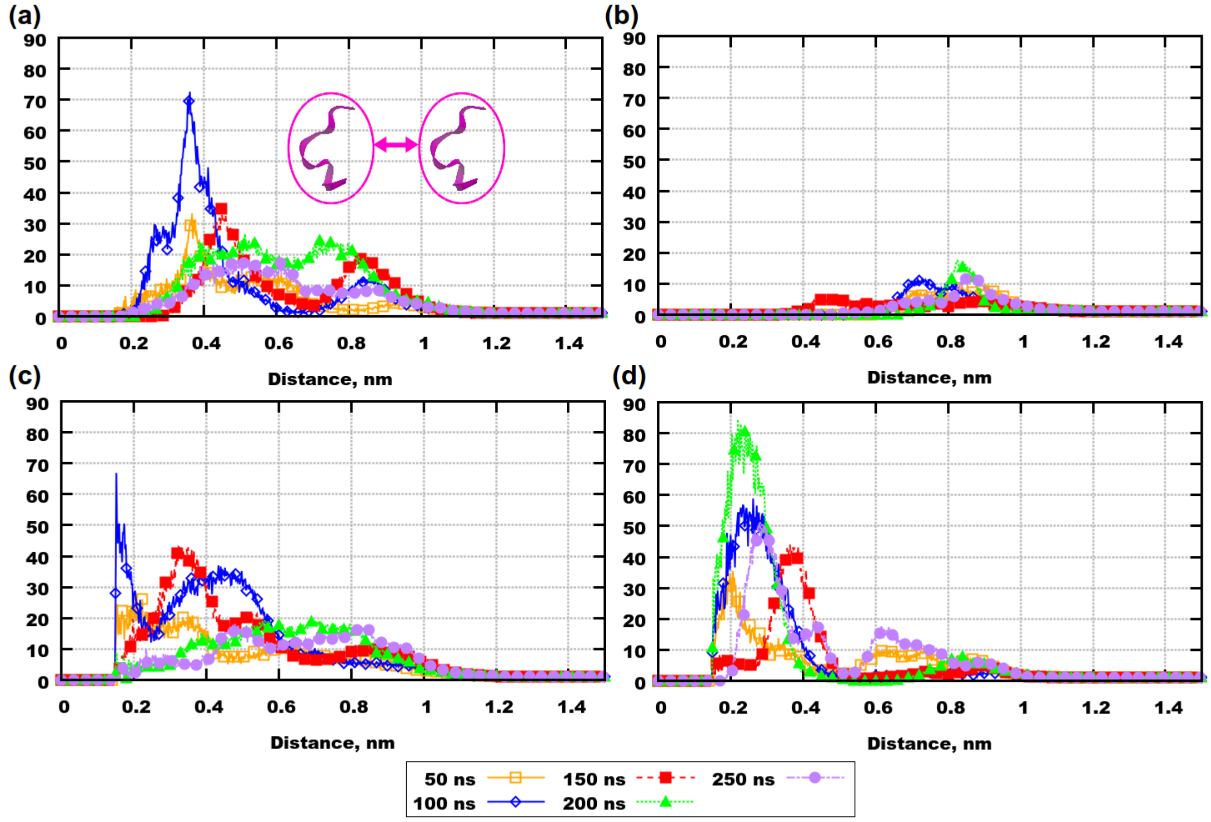

Figure S2: RDFs between centers of mass of peptides for different time intervals. (a) 6 A$\beta(25-35)$ (b) 6 A$\beta(25-35)$ and 6 CBD (c) 8 A$\beta(25-35)$ (d) 8 A$\beta(25-35)$ and 8 CBD.

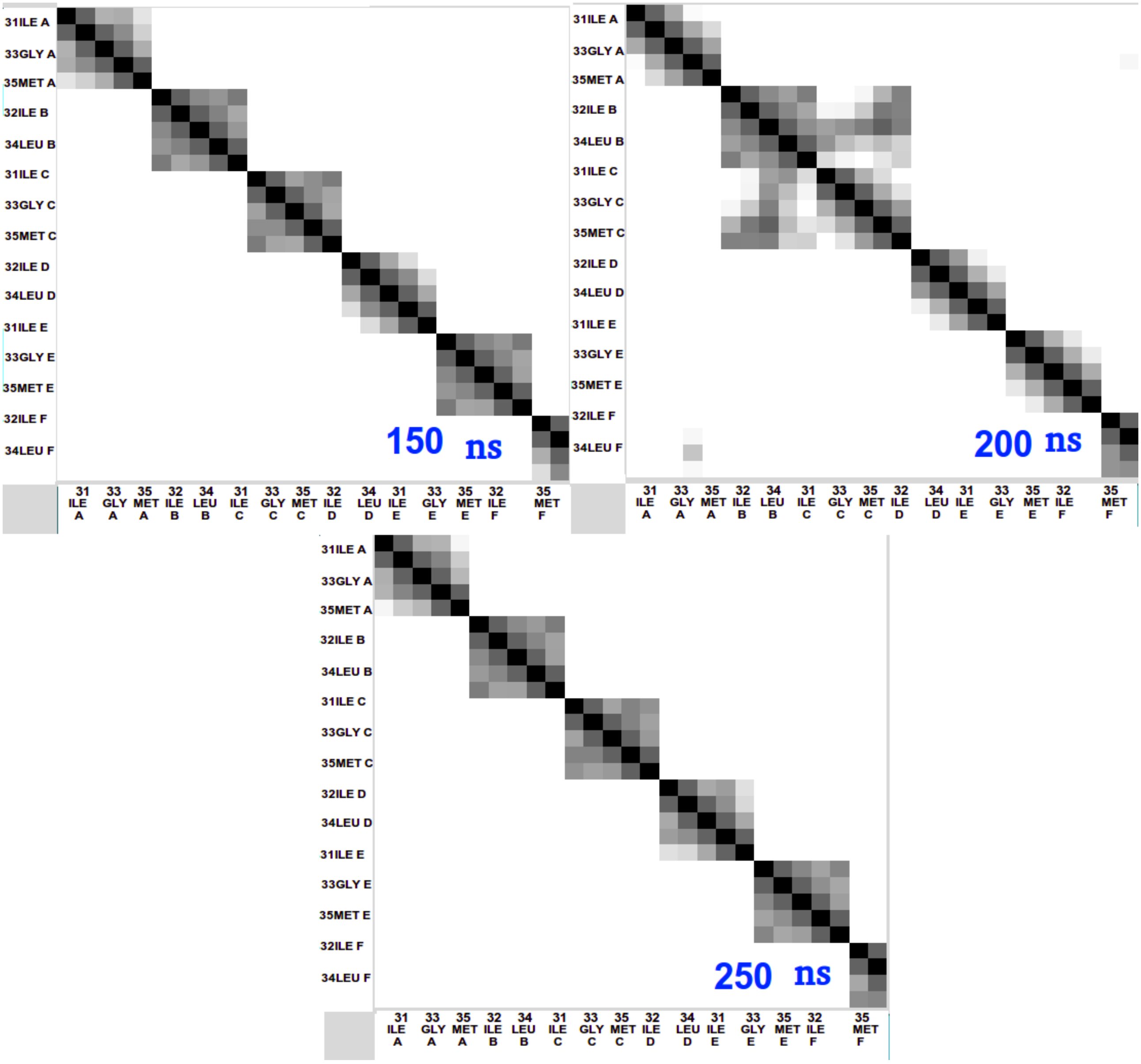

Figure S3: Contact maps for the system with 6 A$\beta(31-35)$ computed in the ends of 3 time intervals.

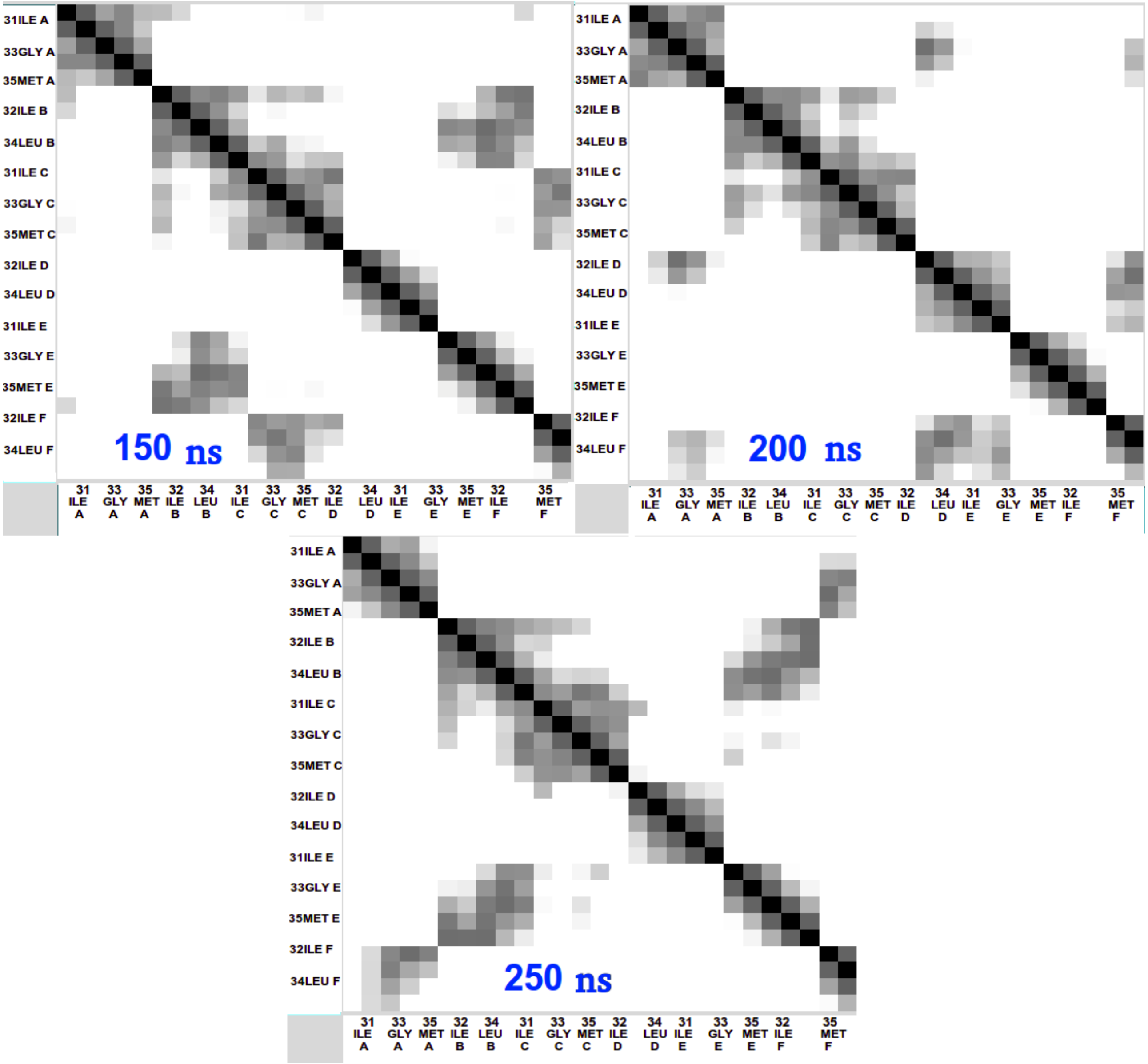

Figure S4: Contact maps for the system with 6 A$\beta(31-35)$ and 6 CBD computed in the ends of 3 time intervals.

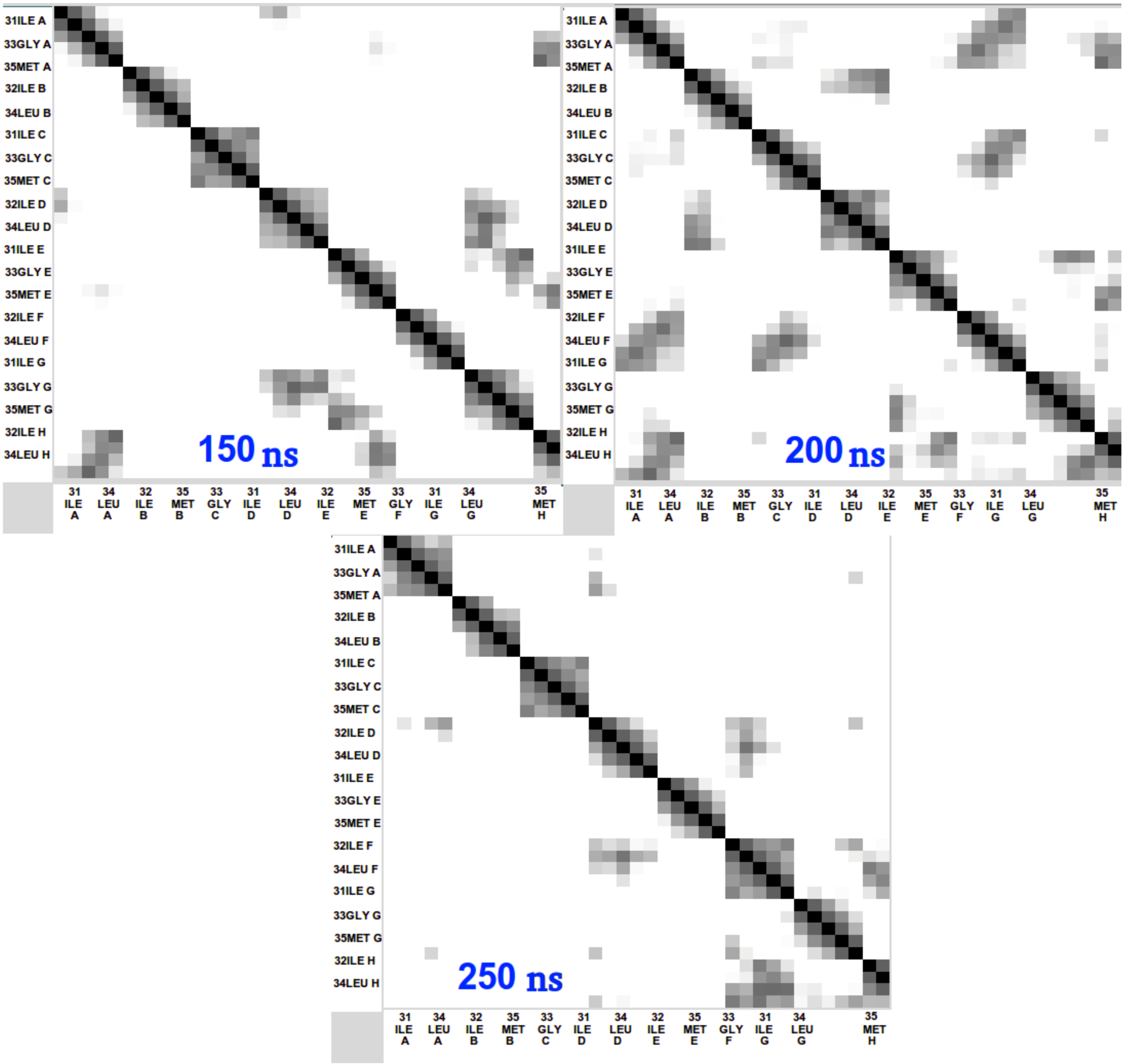

Figure S5: Contact maps for the system with 8 A$\beta(31-35)$ computed in the ends of 3 time intervals.

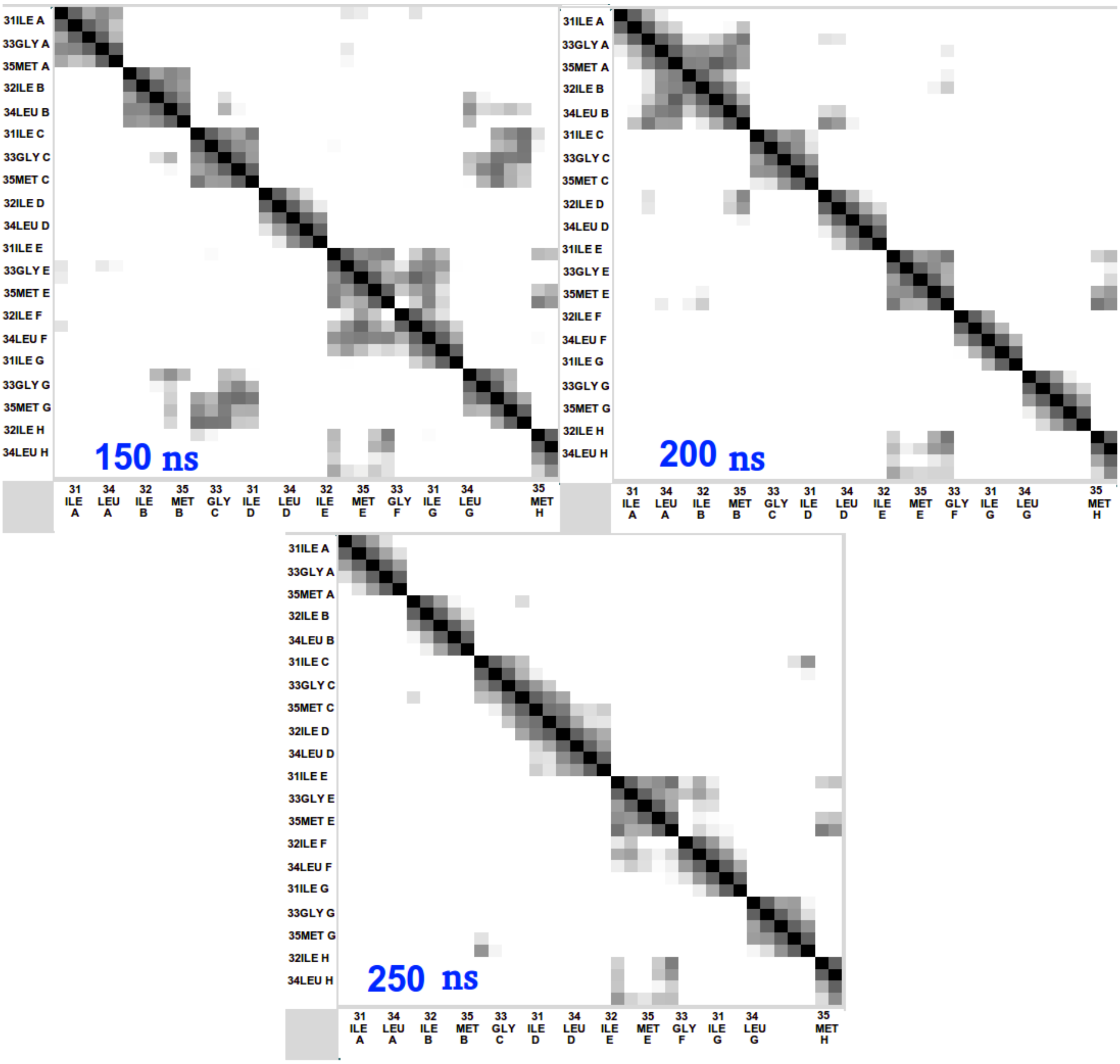

Figure S6: Contact maps for the system with 8 A$\beta(31-35)$ and 8 CBD computed in the ends of 3 time intervals.

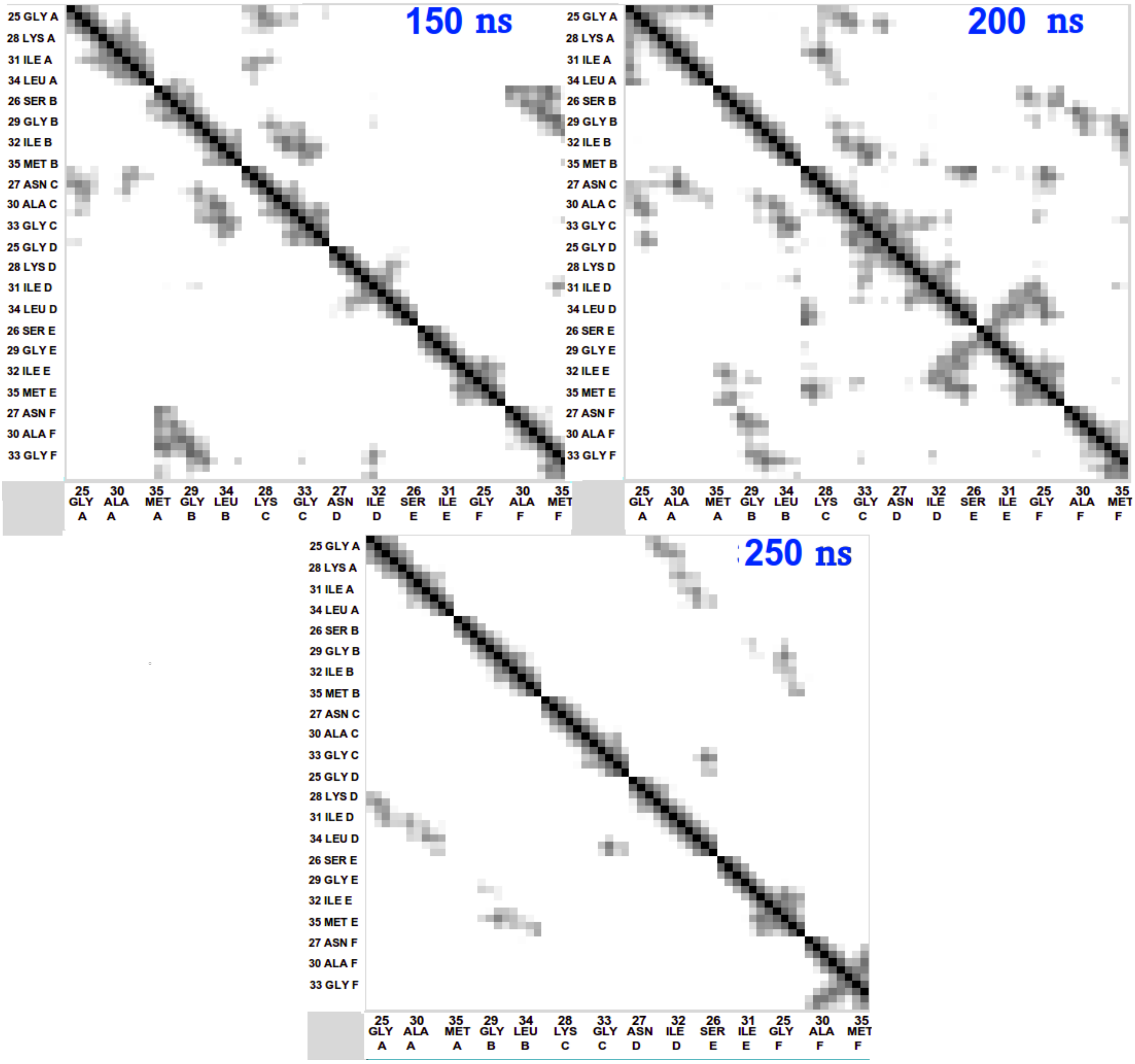

Figure S7: Contact maps for the system with 6 A$\beta(25-35)$ computed in the ends of 3 time intervals.

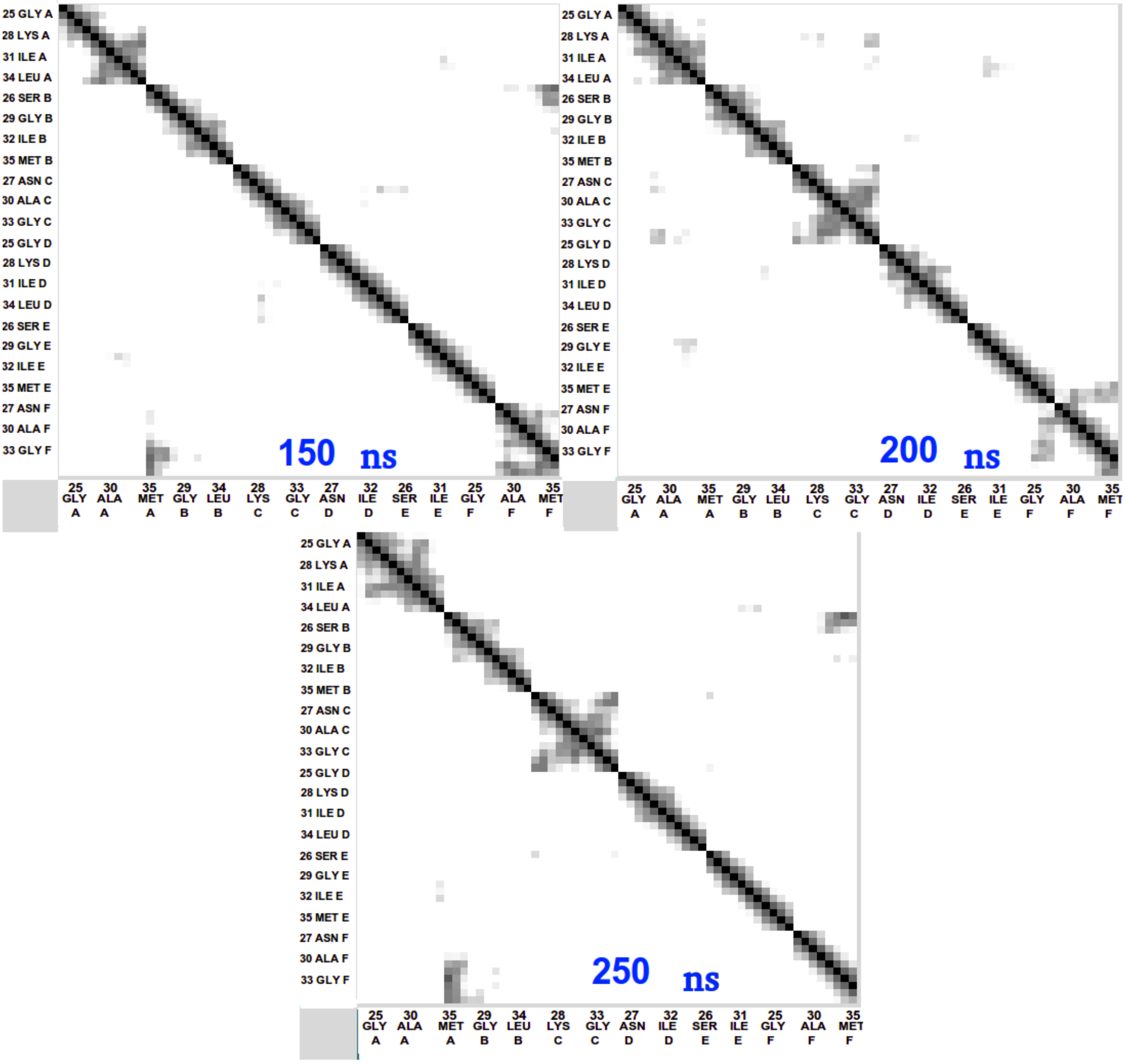

Figure S8: Contact maps for the system with 6 A$\beta(25-35)$ and 6 CBD computed in the ends of 3 time intervals.

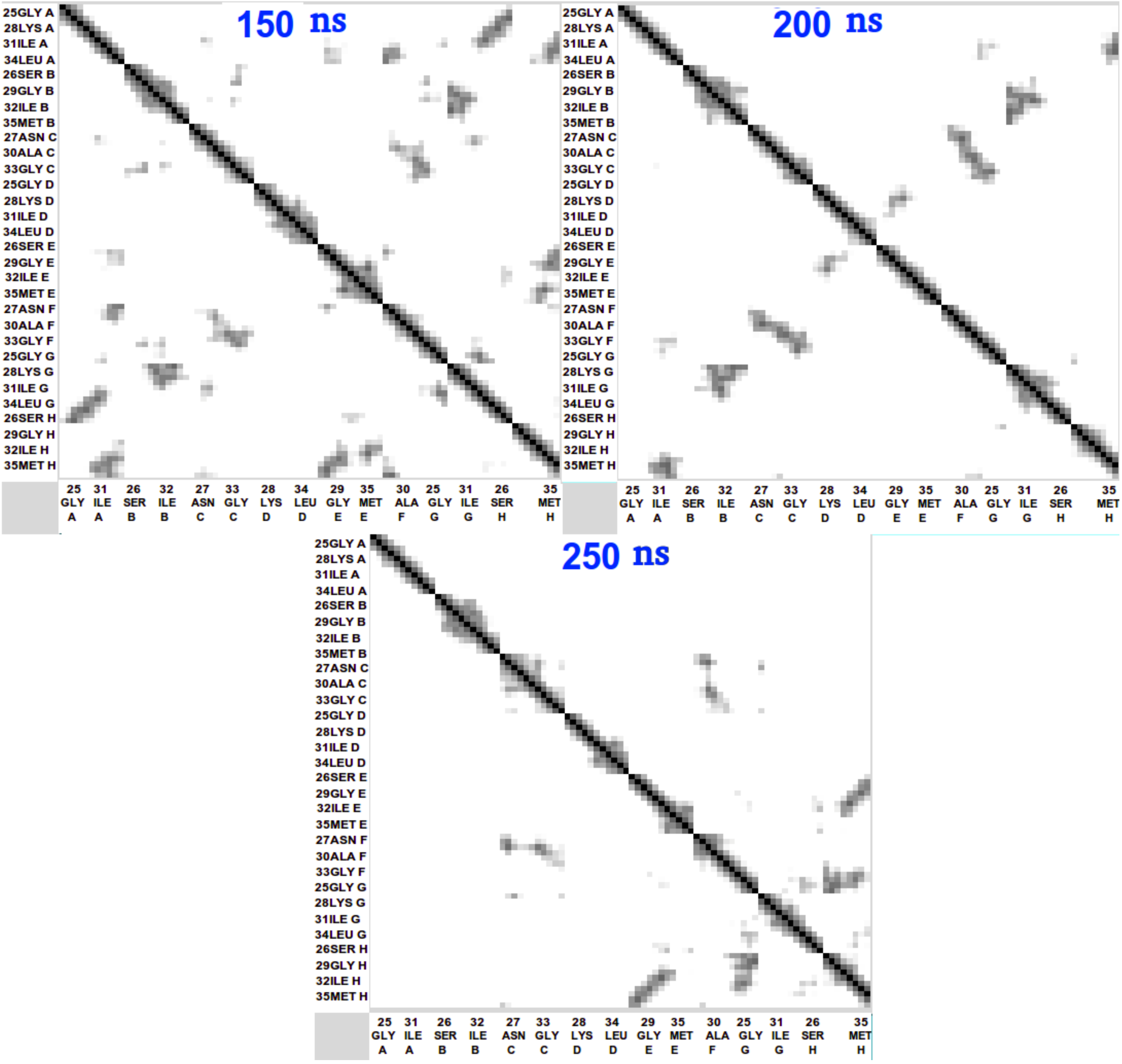

Figure S9: Contact maps for the system with 8 Aβ(25 − 35) computed in the ends of 3 time intervals.

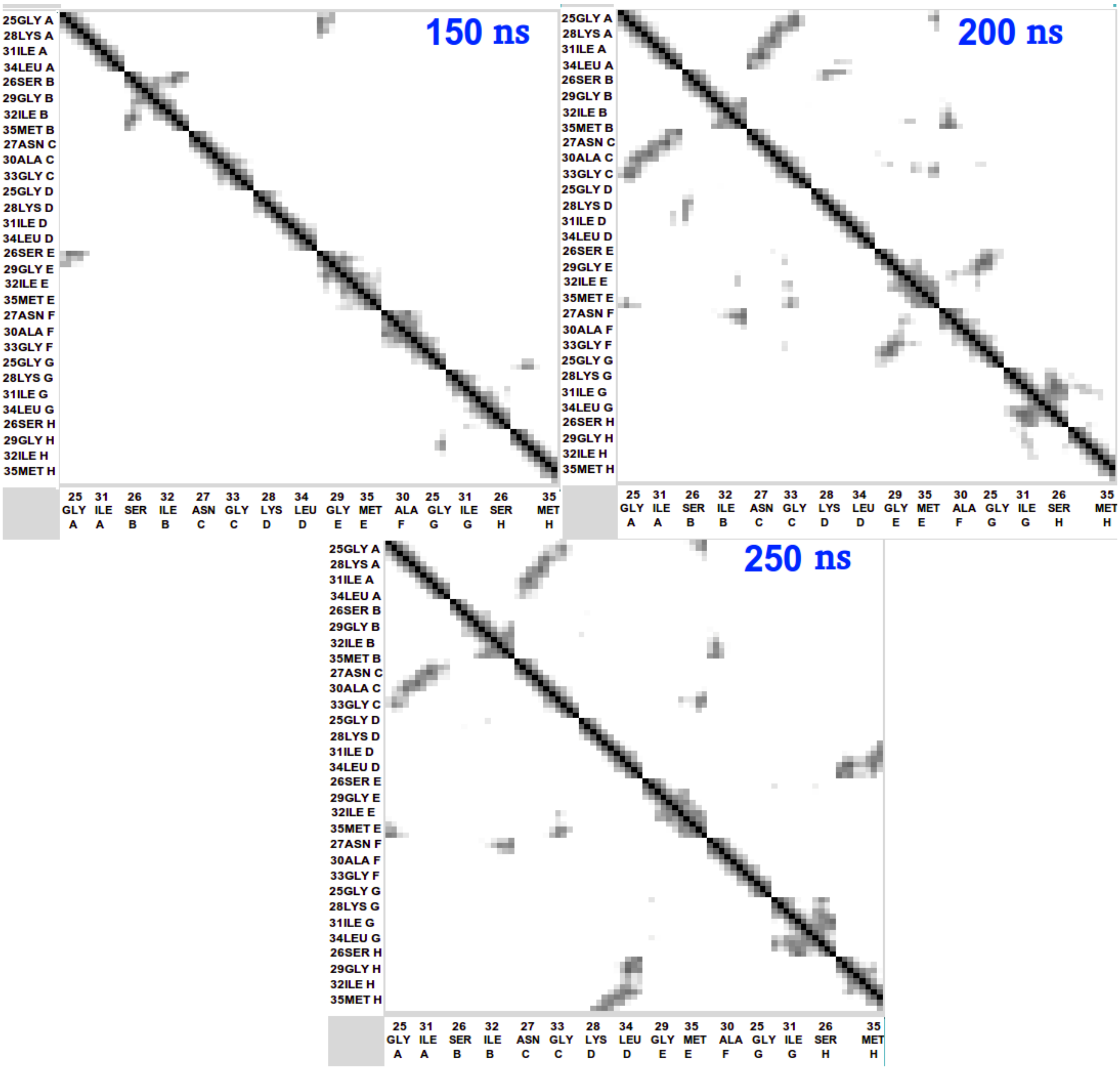

Figure S10: Contact maps for the system with 8 A$\beta(25-35)$ and 8 CBD computed in the ends of 3 time intervals.

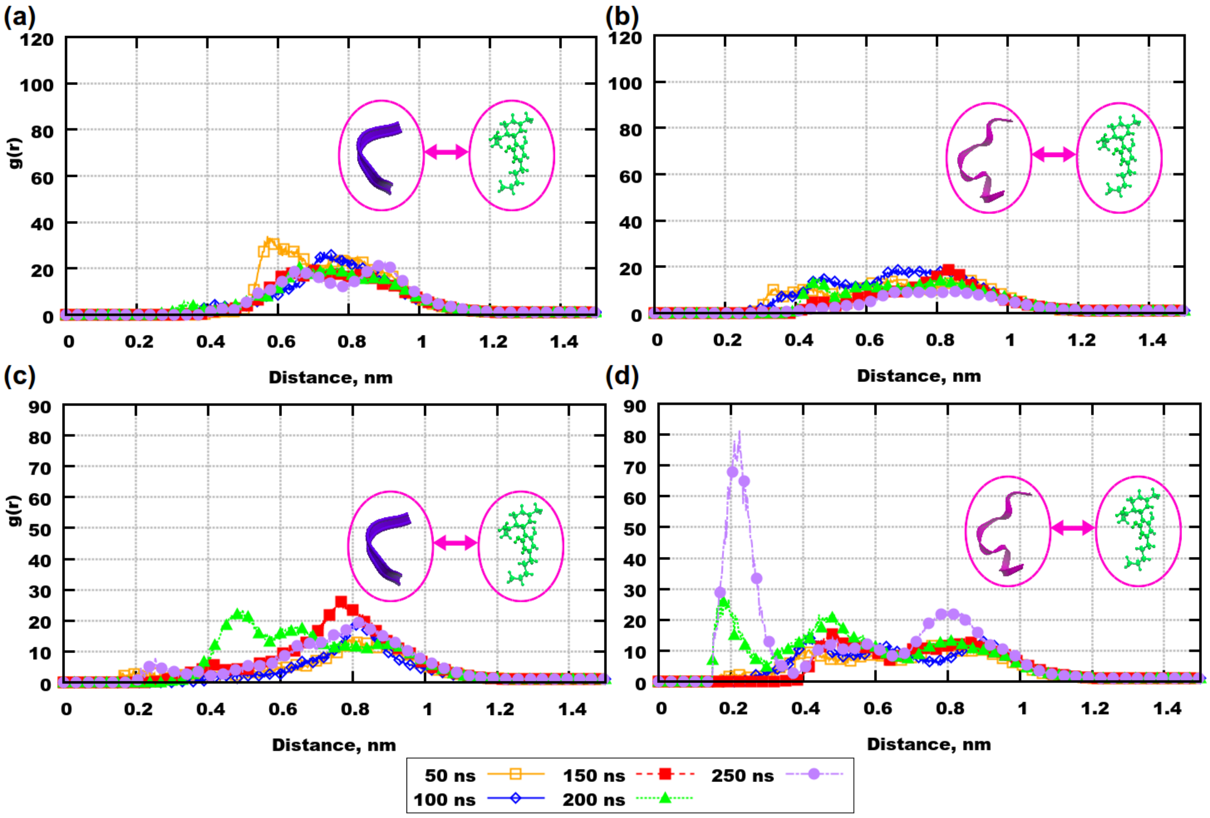

Figure S11: RDFs between centers of mass of CBD and peptides in systems for different time intervals. (a) 6 Aβ(31 − 35) and 6 CBD (b) 6 Aβ(25 − 35) and 6 CBD (c) 8 Aβ(31 − 35) and 8 CBD (d) 8 Aβ(25 − 35) and 8 CBD.

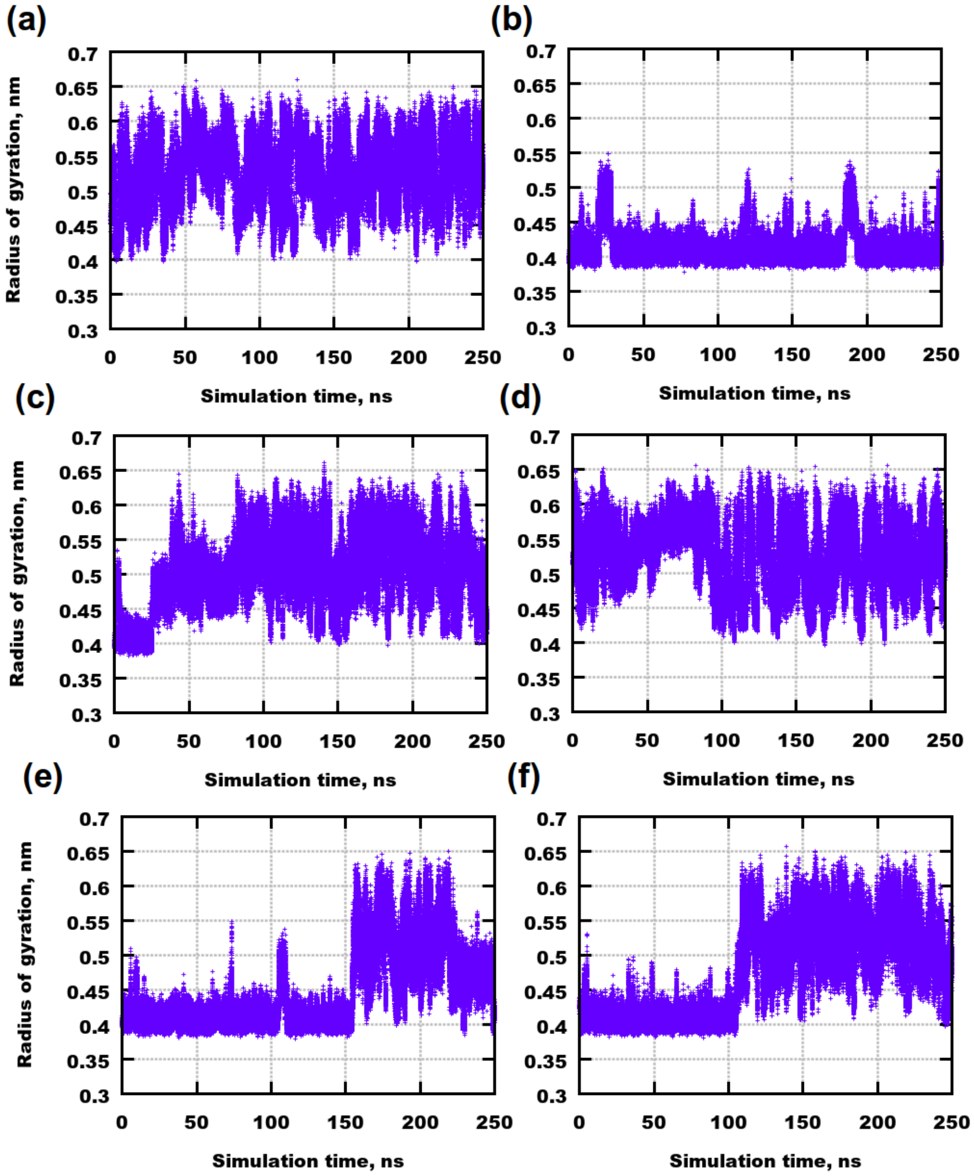

Figure S12: Radius of gyration of peptides in the system with 6 Aβ(31 − 35). Letters (a)-(f) denote separate peptides.

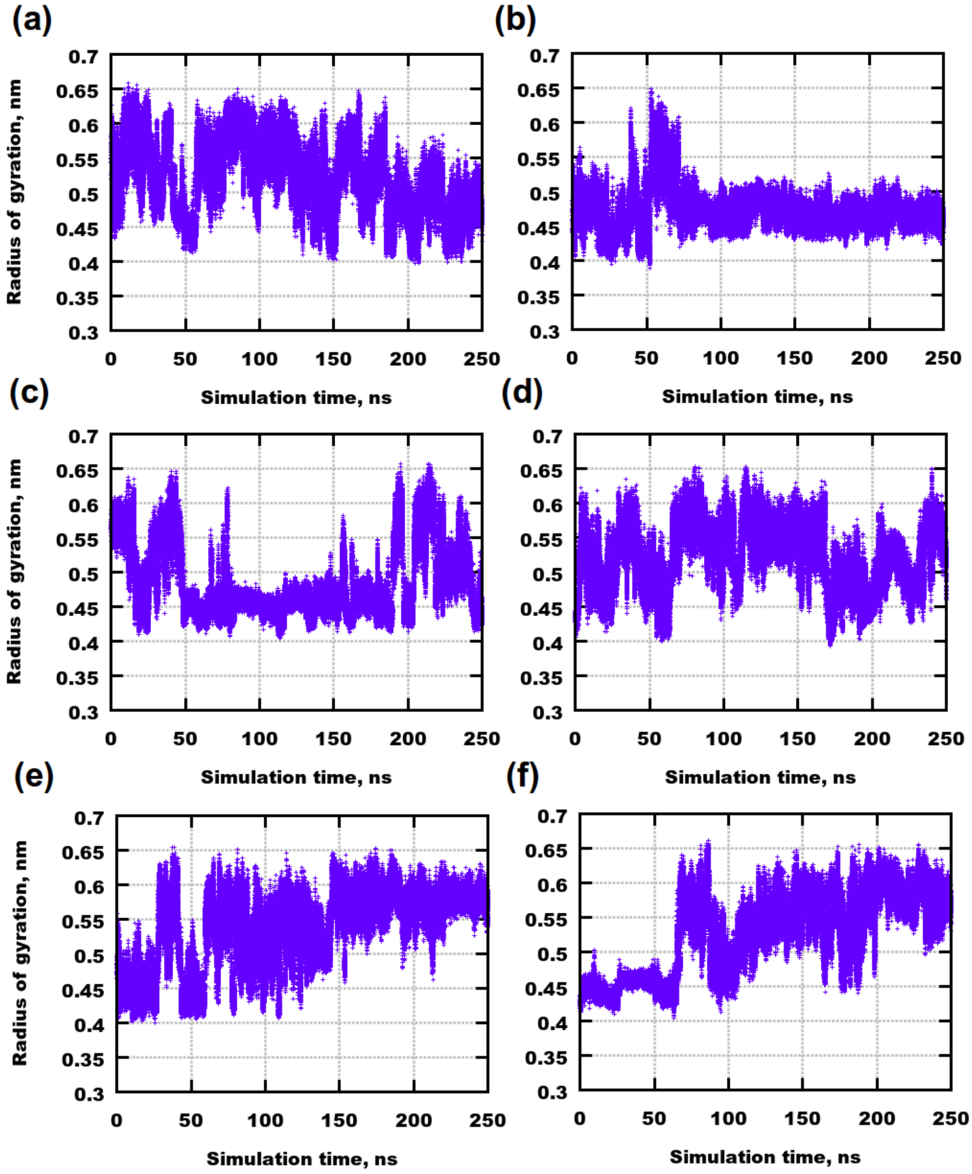

Figure S13: Radius of gyration of peptides in the system with 6 Aβ(31 − 35) an 6 CBD. Letters (a)-(f) denote separate peptides.

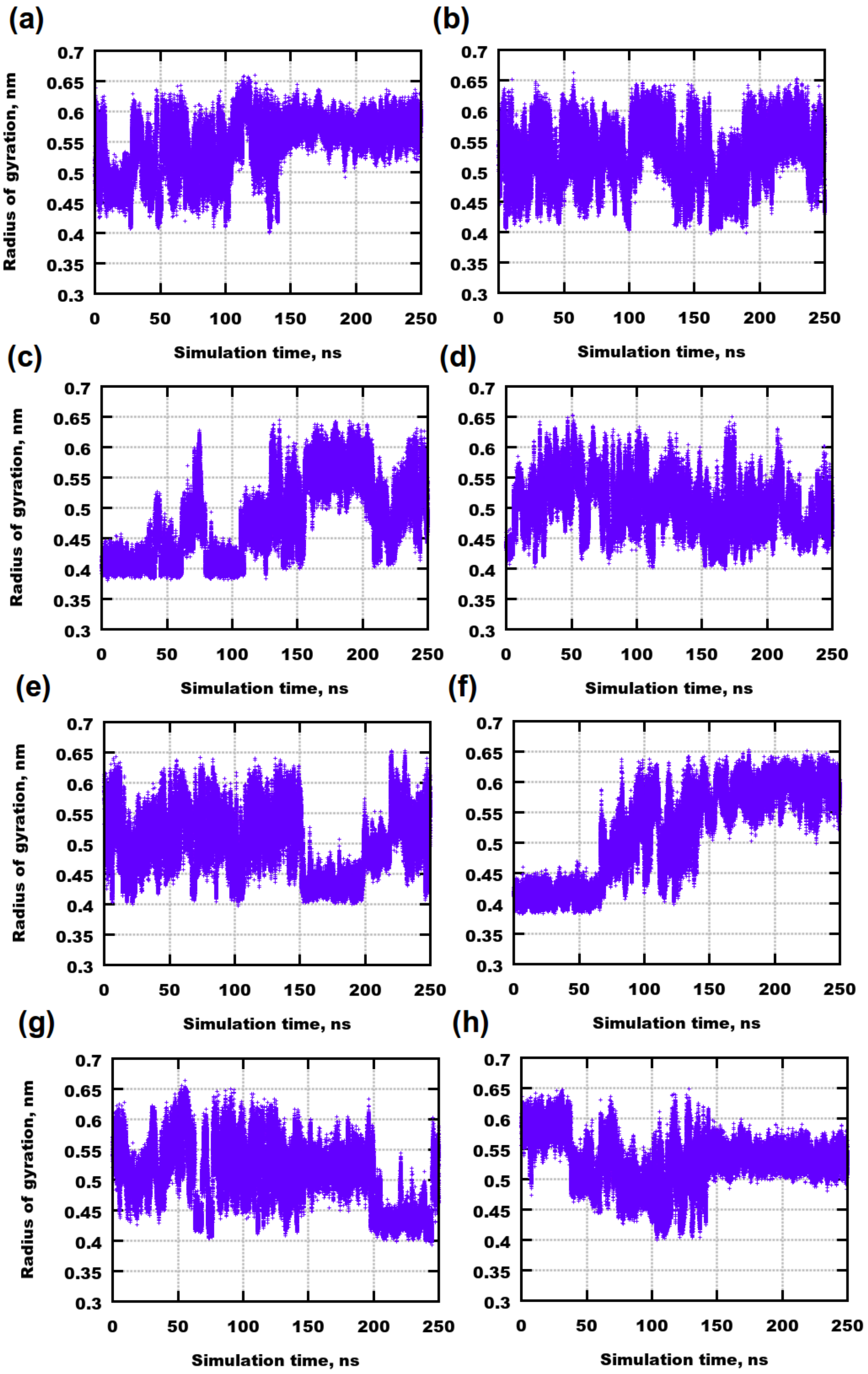

Figure S14: Radius of gyration of peptides in the system with 8 Aβ(31 − 35). Letters (a)-(h) denote separate peptides.

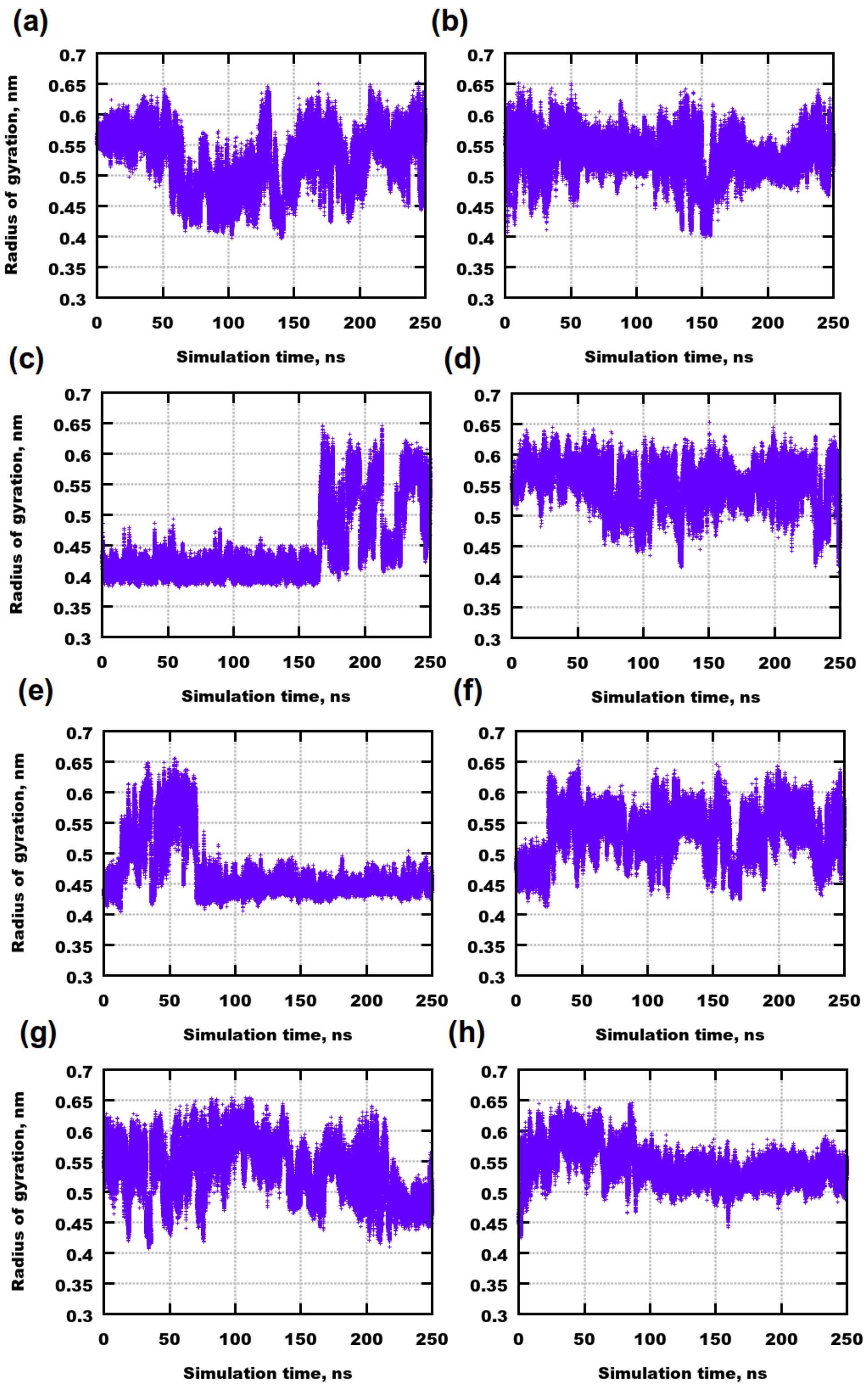

Figure S15: Radius of gyration of peptides in the system with 8 A$\beta(31-35)$ an 8 CBD. Letters (a)-(h) denote separate peptides.

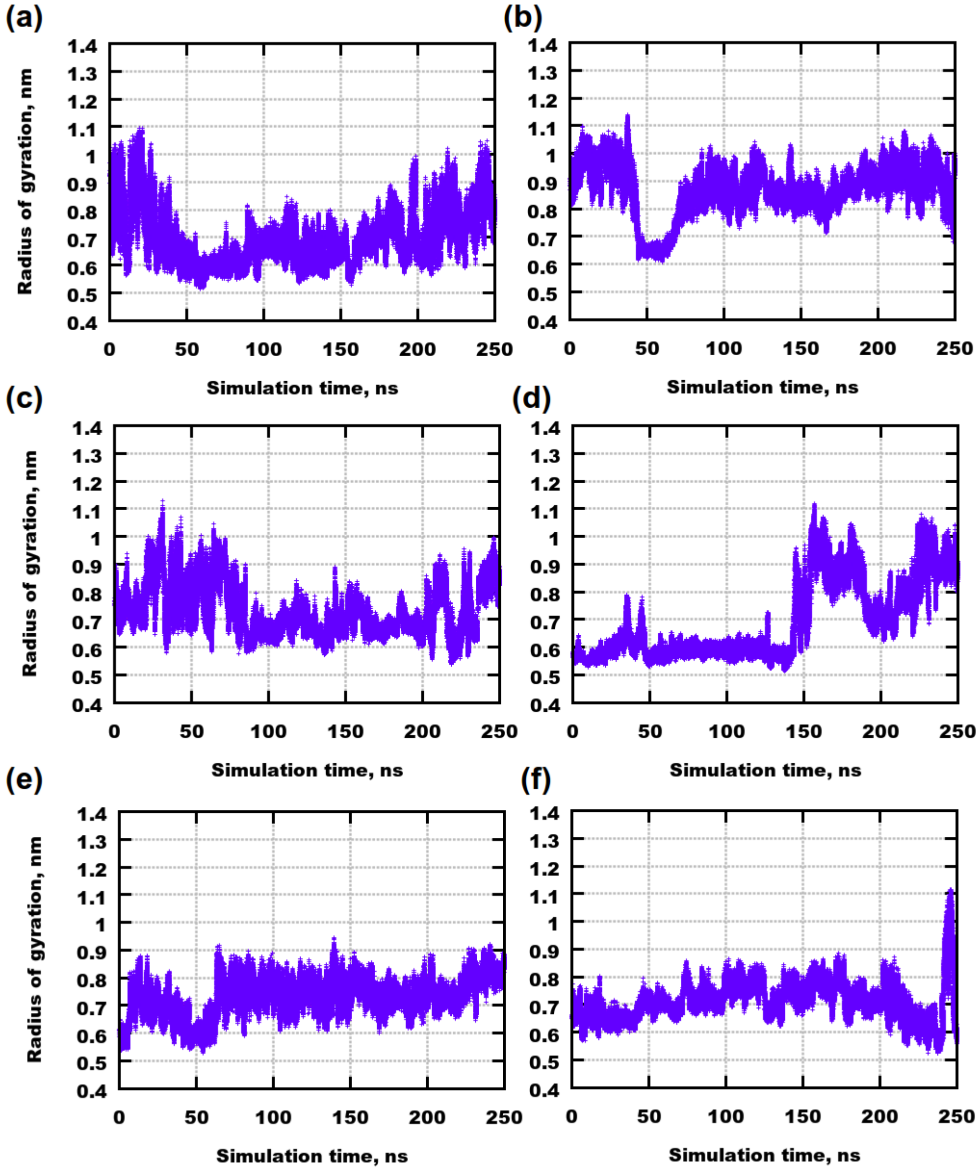

Figure S16: Radius of gyration of peptides in the system with 6 A$\beta(25-35)$. Letters (a)-(f) denote separate peptides.

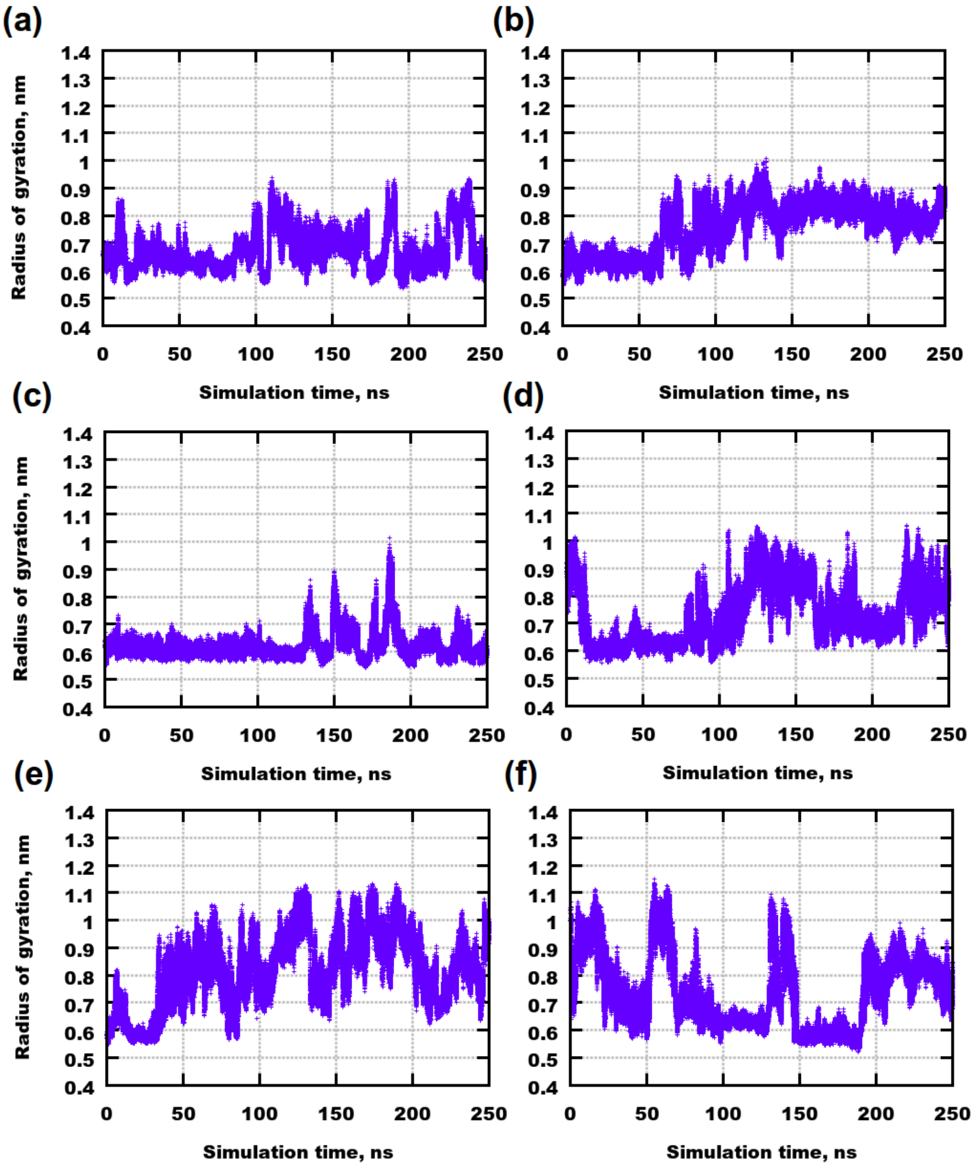

Figure S17: Radius of gyration of peptides in the system with 6 Aβ(25 − 35) an 6 CBD. Letters (a)-(f) denote separate peptides.

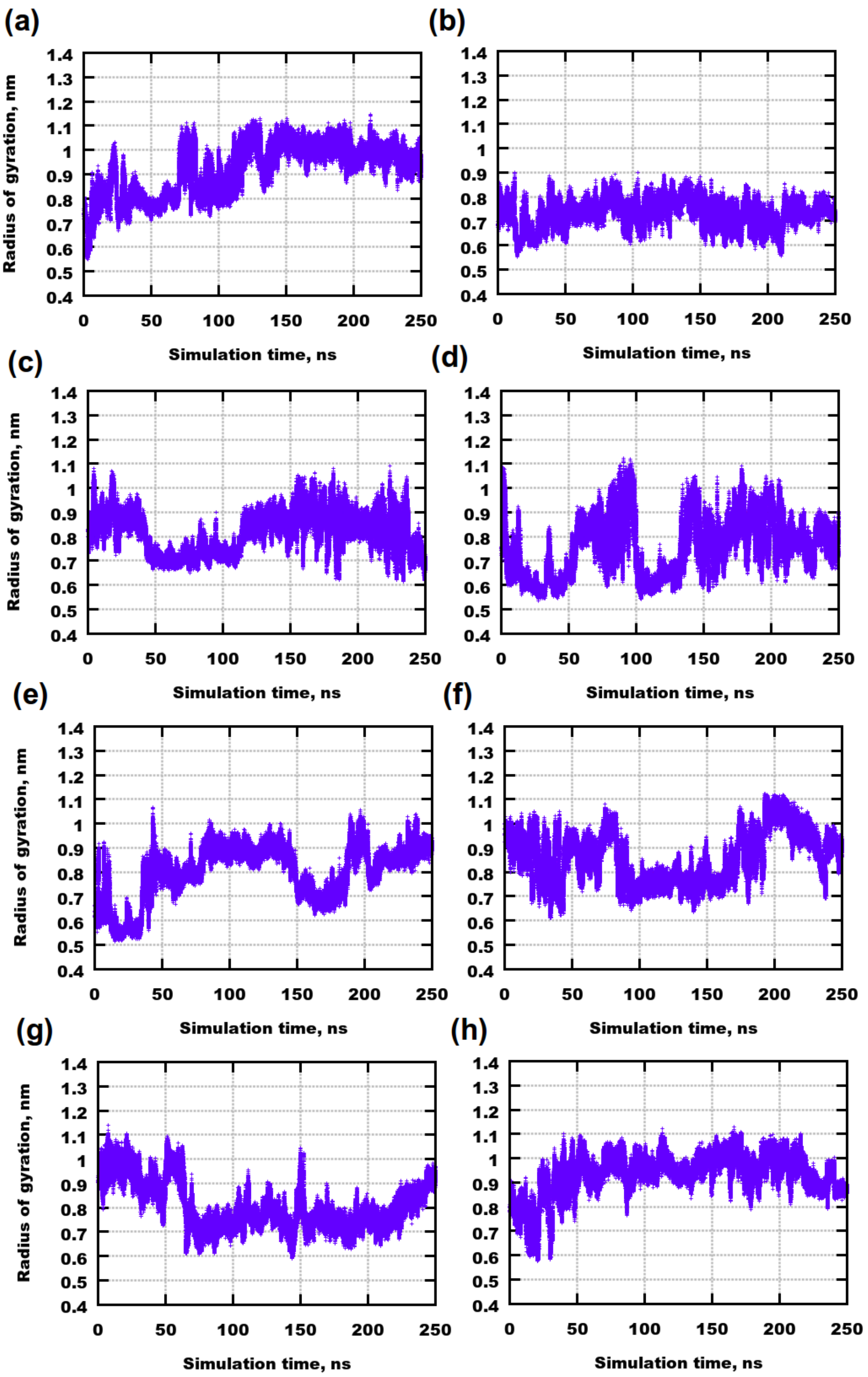

Figure S18: Radius of gyration of peptides in the system with 8 A$\beta(25-35)$. Letters (a)-(h) denote separate peptides.

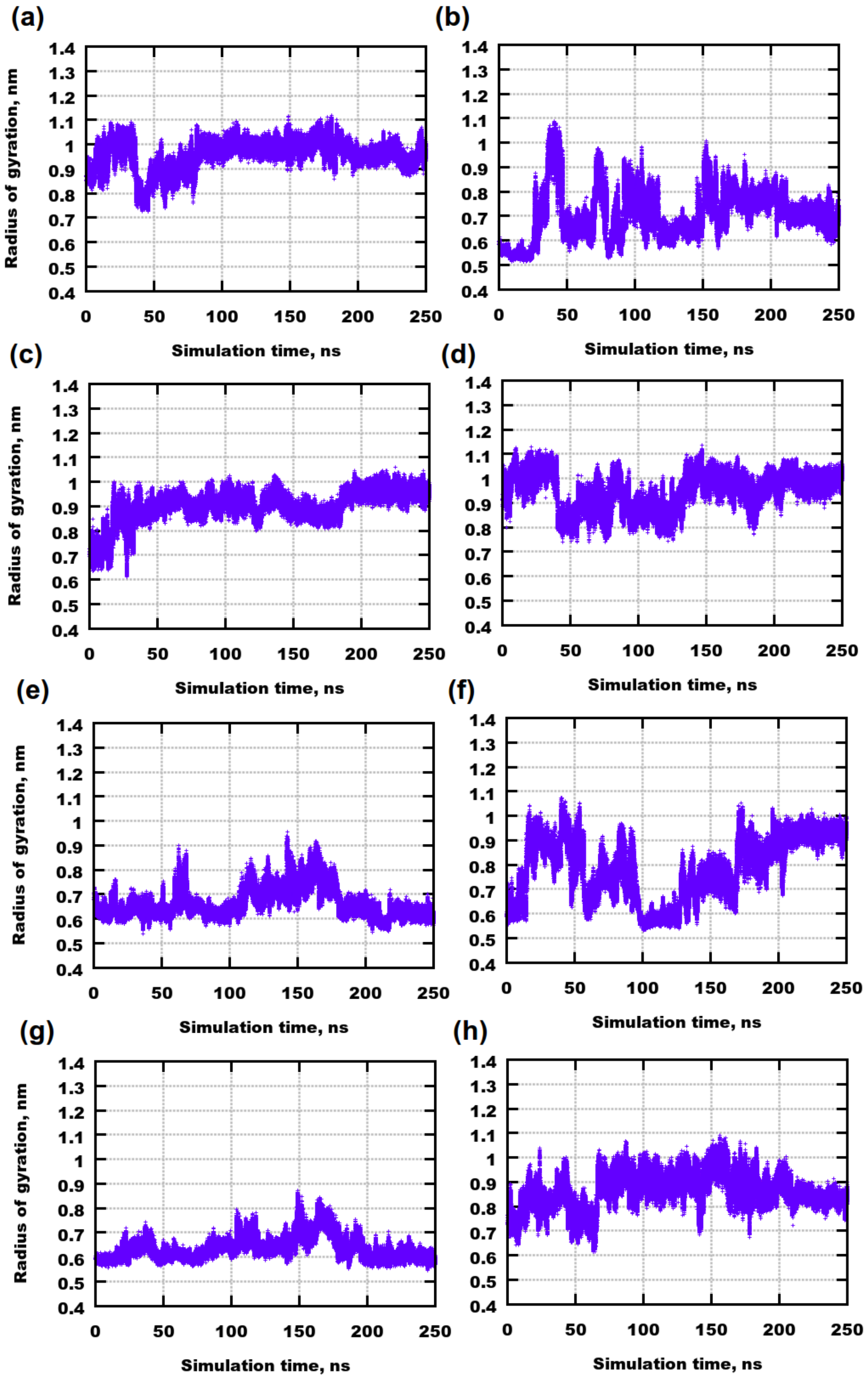

Figure S19: Radius of gyration of peptides in the system with 8 A$\beta(25-35)$ an 8 CBD. Letters (a)-(h) denote separate peptides.

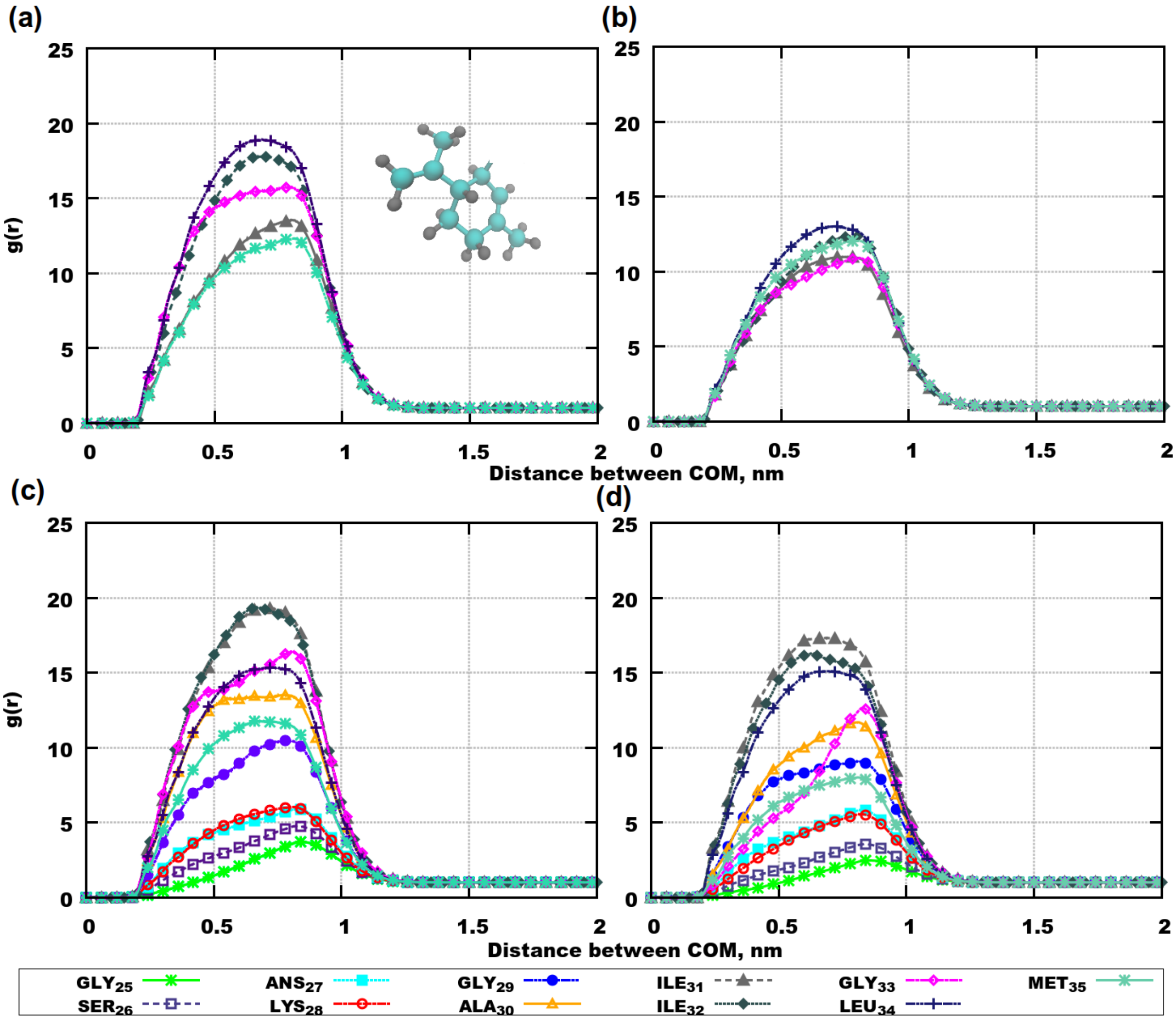

Figure S20: RDFs between the centers of mass of amino acid residues and the selected part of the CBD molecule (the cyclohexene ring with two radicals). (a) 6 A$\beta(31-35)$ and 6 CBD (b) 6 A$\beta(25-35)$ and 6 CBD (c) 8 A$\beta(31-35)$ and 8 CBD (d) 8 A$\beta(25-35)$ and 8 CBD.

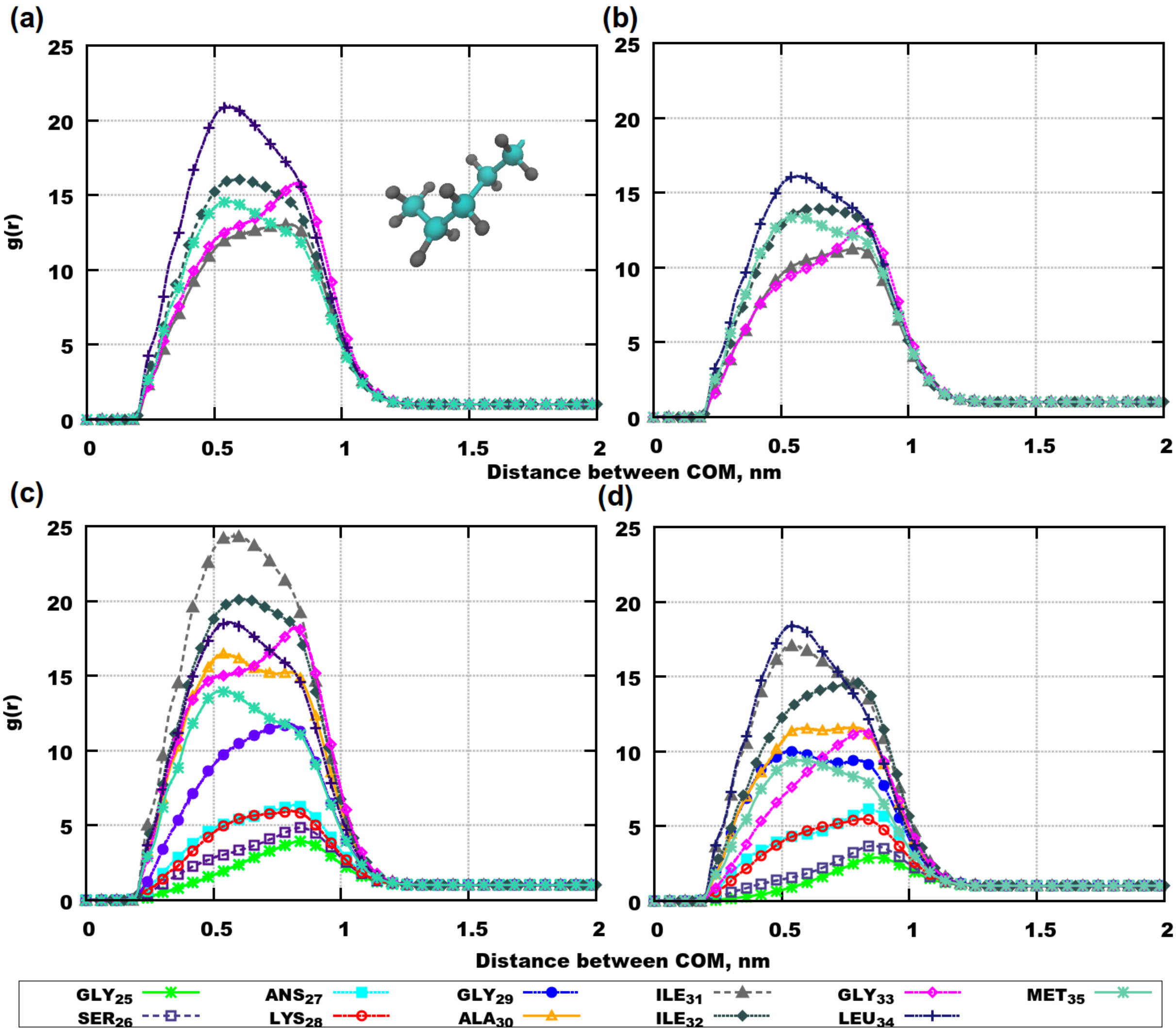

Figure S21: RDFs between the centers of mass of amino acid residues and the selected part of the CBD molecule (the pentyl "tail"). (a) 6 Aβ(31 − 35) and 6 CBD (b) 6 Aβ(25 − 35) and 6 CBD (c) 8 Aβ(31 − 35) and 8 CBD (d) 8 Aβ(25 − 35) and 8 CBD.

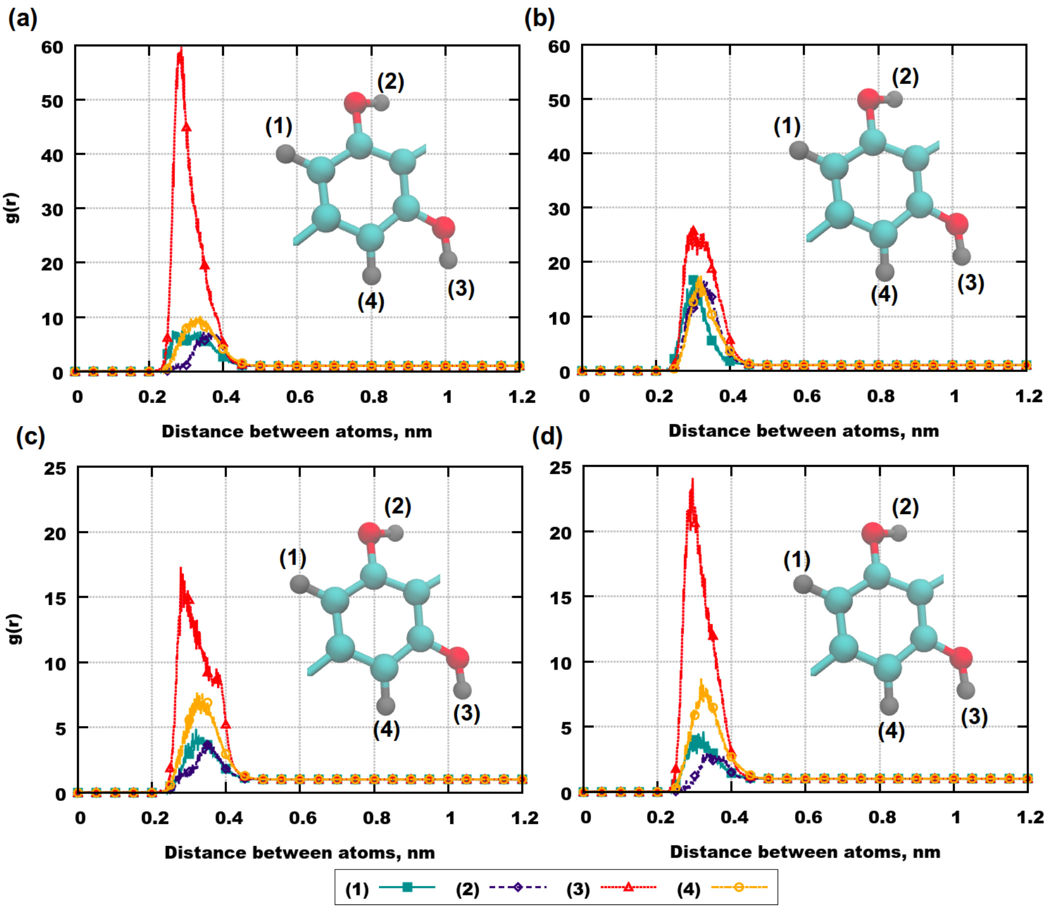

Figure S22: RDFs between selected hydrogen atoms from the dihydroxyphenyl ring of CBD and a nitrogen atom from MET_{35}. (a) 6 A$\beta(31-35)$ and 6 CBD, (b) 6 A$\beta(25-35)$ and 6 CBD, (c) 8 A$\beta(31-35)$ and 8 CBD, (d) 8 A$\beta(25-35)$ and 8 CBD.

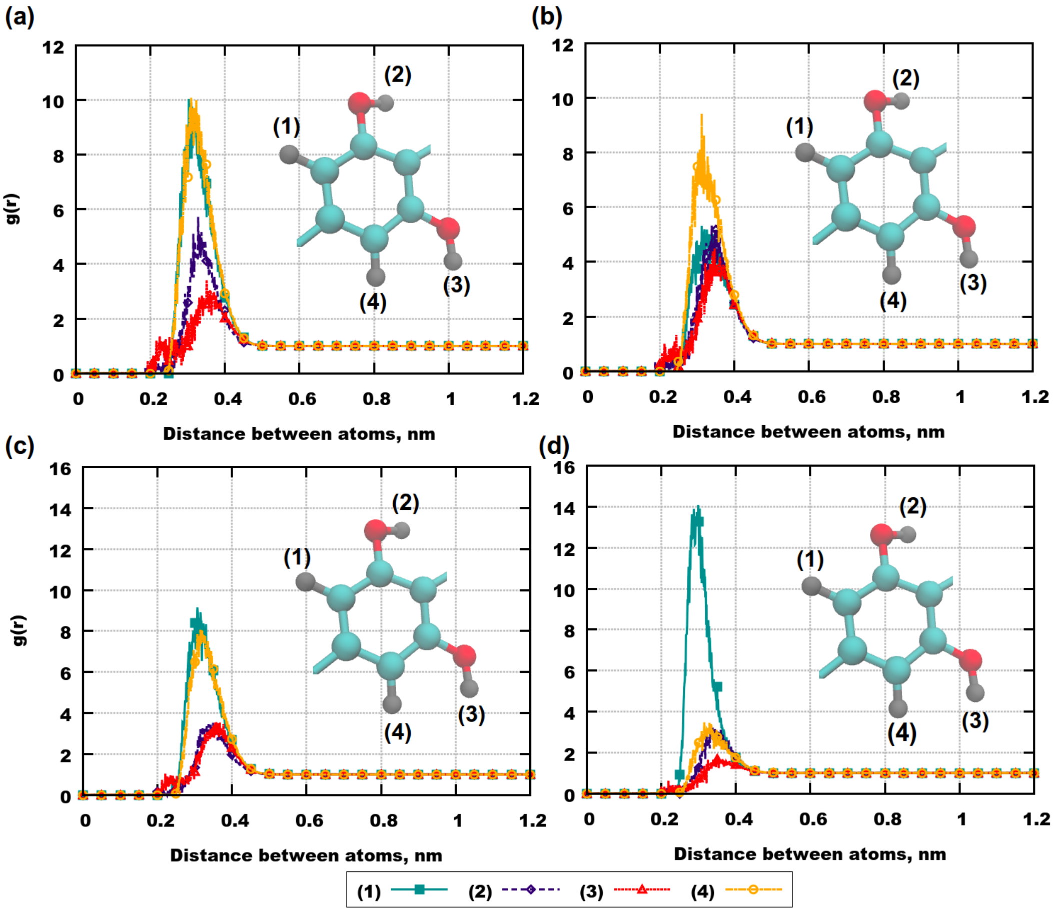

Figure S23: RDFs between selected hydrogen atoms from dihydroxyphenyl ring of CBD and a sulfur atom from MET_{35}. (a) 6 A$\beta(31-35)$ and 6 CBD, (b) 6 A$\beta(25-35)$ and 6 CBD, (c) 8 A$\beta(31-35)$ and 8 CBD, (d) 8 A$\beta(25-35)$ and 8 CBD.

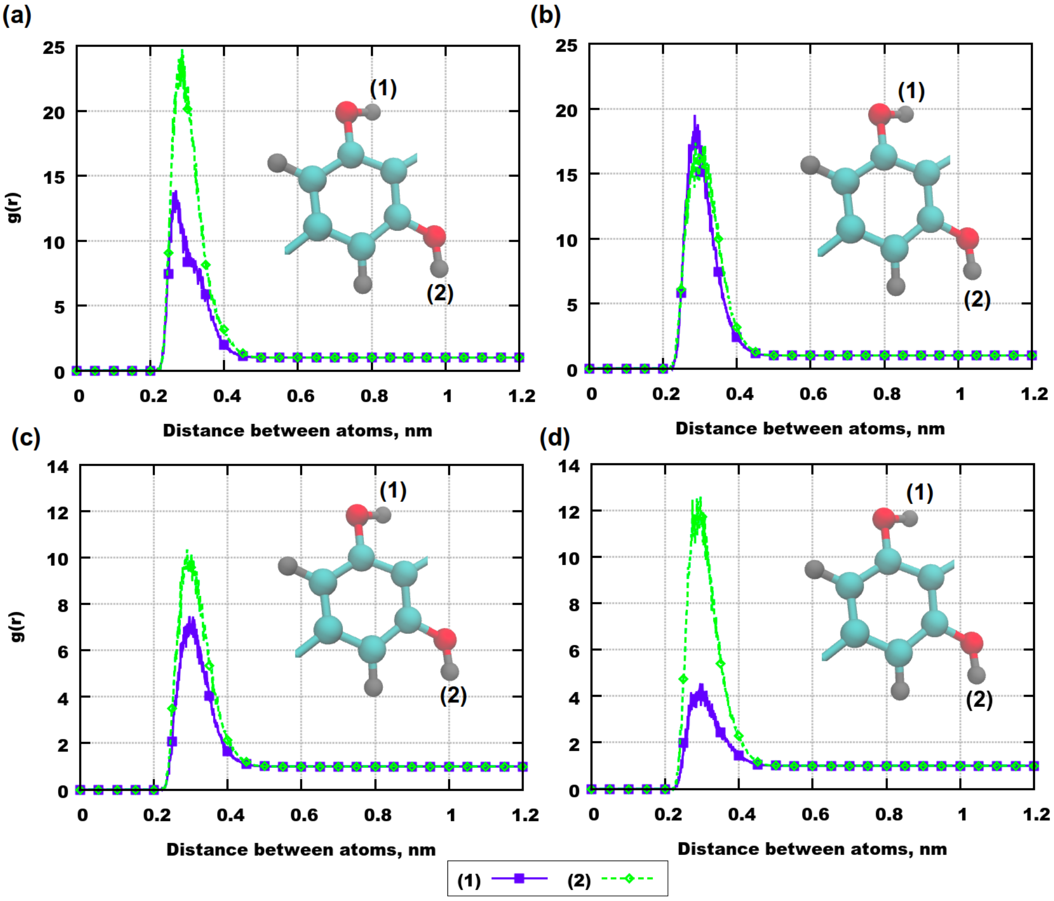

Figure S24: RDFs between selected oxygen atoms from the dihydroxyphenyl ring of CBD and hydrogen atoms from the methyl group in MET_{35}. (a) 6 Aβ(31 − 35) and 6 CBD, (b) 6 Aβ(25 − 35) and 6 CBD, (c) 8 Aβ(31 − 35) and 8 CBD, (d) 8 Aβ(25 − 35) and 8 CBD.

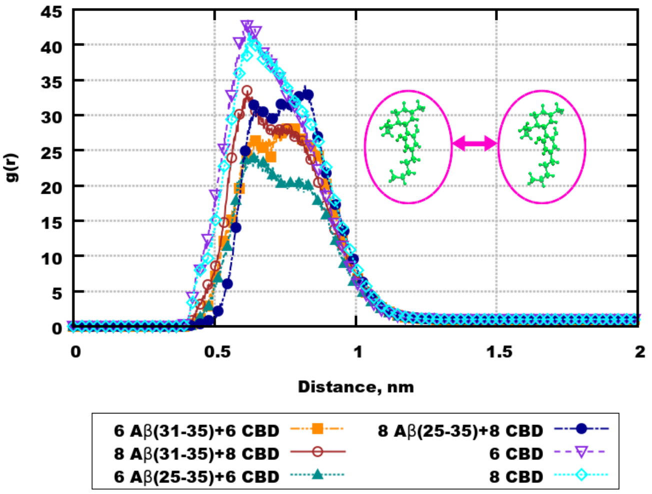

Figure S25: RDFs between centers of mass of CBD molecules in simulated systems during 250 ns.

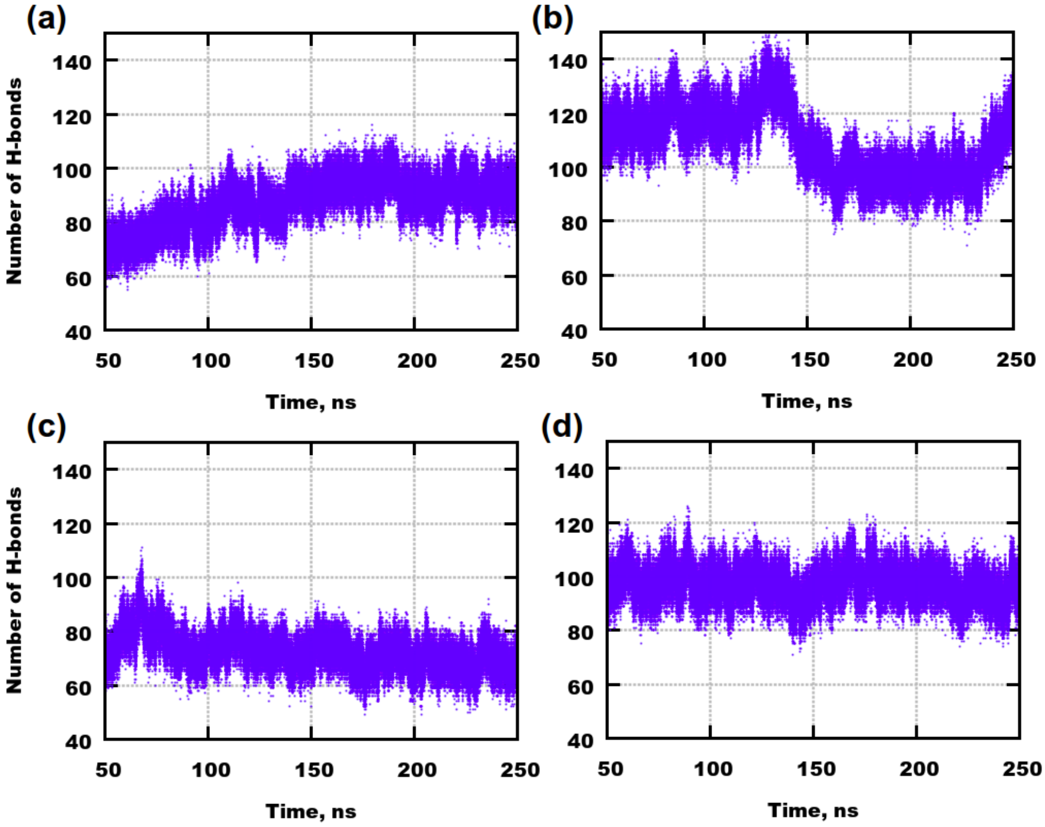

Figure S26: Hydrogen bonds formed between atoms in A$\beta(31-35)$ and atoms in water molecules. Considered distances were between 0 and 0.35 nm. (a) System with 6 A$\beta(31-35)$ in water, (b) system with 8 A$\beta(31-35)$ in water, (c) system with 6 A$\beta(31-35)$ and 6 CBD in water, (d) system with 8 A$\beta(31-35)$ and 8 CBD in water.

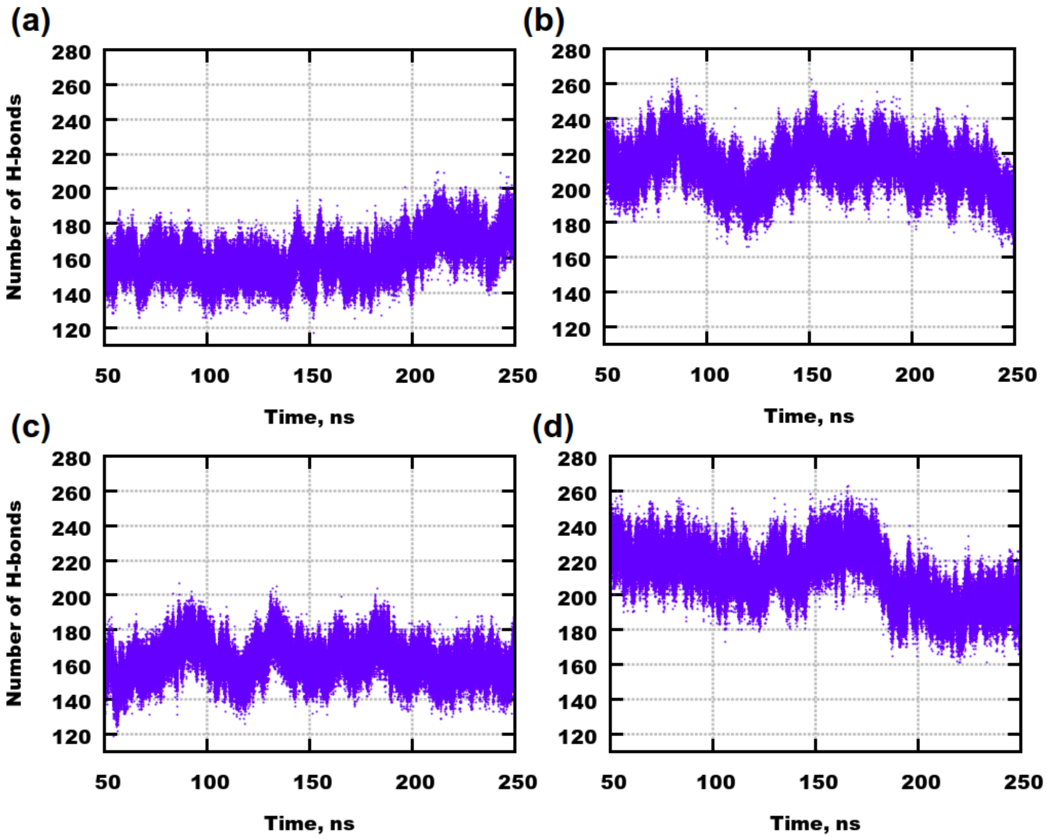

Figure S27: Hydrogen bonds formed between atoms in Aβ(25 − 35) and atoms in water molecules. Considered distances were between 0 and 0.35 nm. (a) System with 6 Aβ(25 − 35) in water, (b) system with 8 Aβ(25 − 35) in water, (c) system with 6 Aβ(25 − 35) and 6 CBD in water, (d) system with 8 Aβ(25 − 35) and 8 CBD in water.

1.3 Secondary structures of peptides

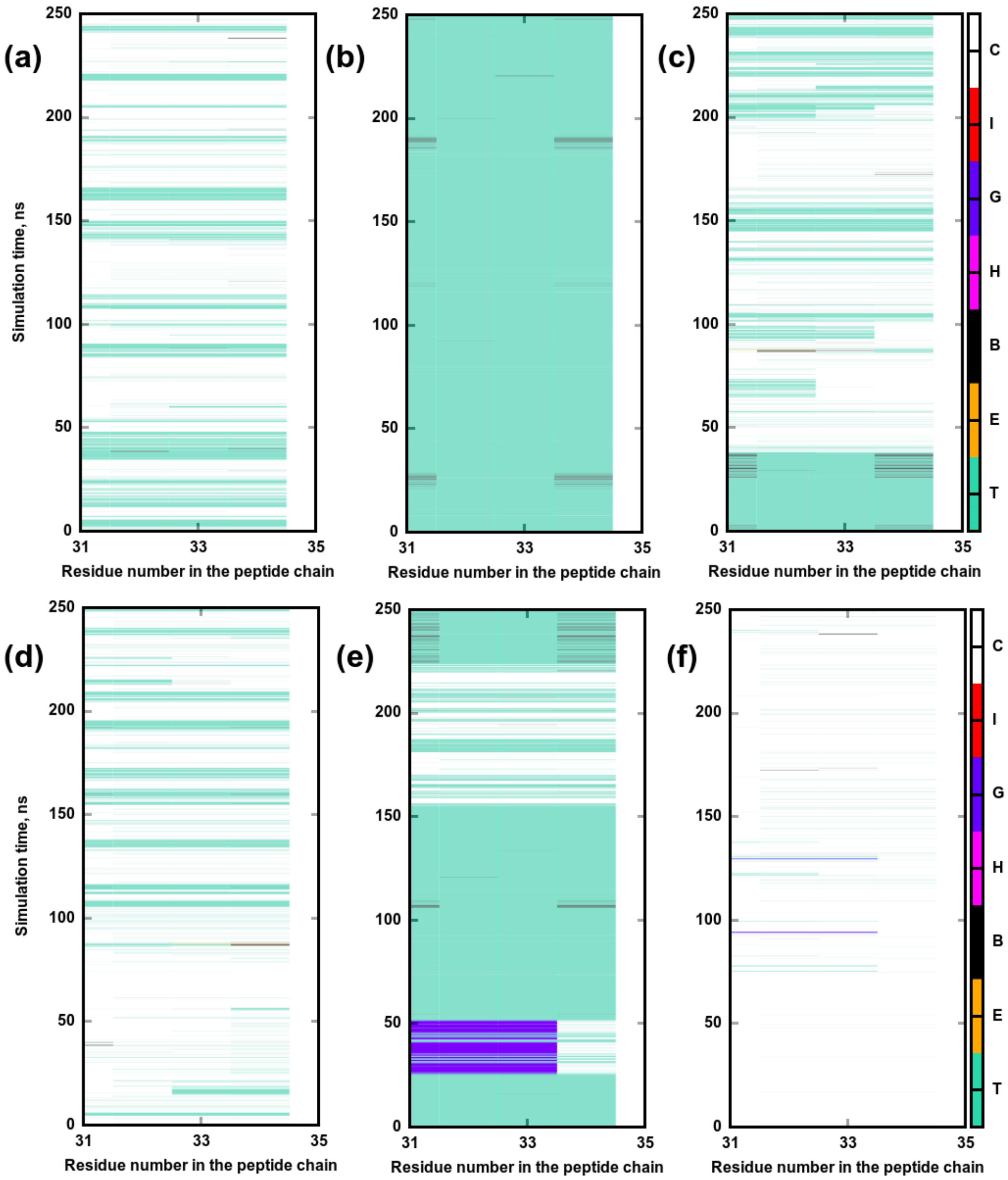

Figure S28: Secondary structures of peptides for systems containing 6 Aβ(31 − 35) without CBD during production runs. Letters (a)-(f) denote separate peptides. T - turn, E - extended conformation, B - isolated β-bridge, H - α-helix, G - 3_{10}-helix, I - π-helix, C - coil.

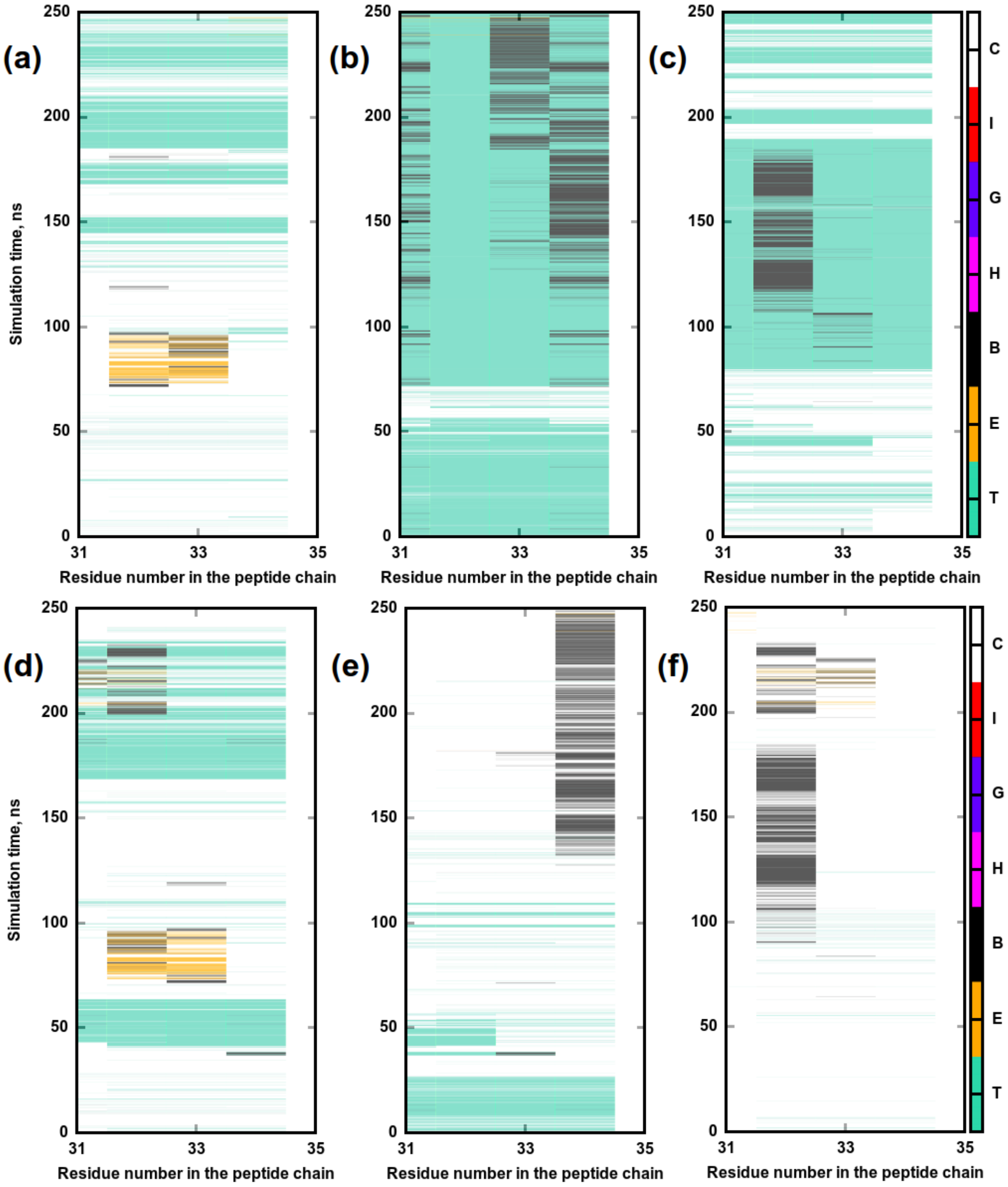

Figure S29: Secondary structures of peptides for systems containing 6 A$\beta(31-35)$ with 6 CBD molecules during production runs. Letters (a)-(f) denote separate peptides. T - turn, E - extended conformation, B - isolated β-bridge, H - α-helix, G - 3_{10}-helix, I - π-helix, C - coil.

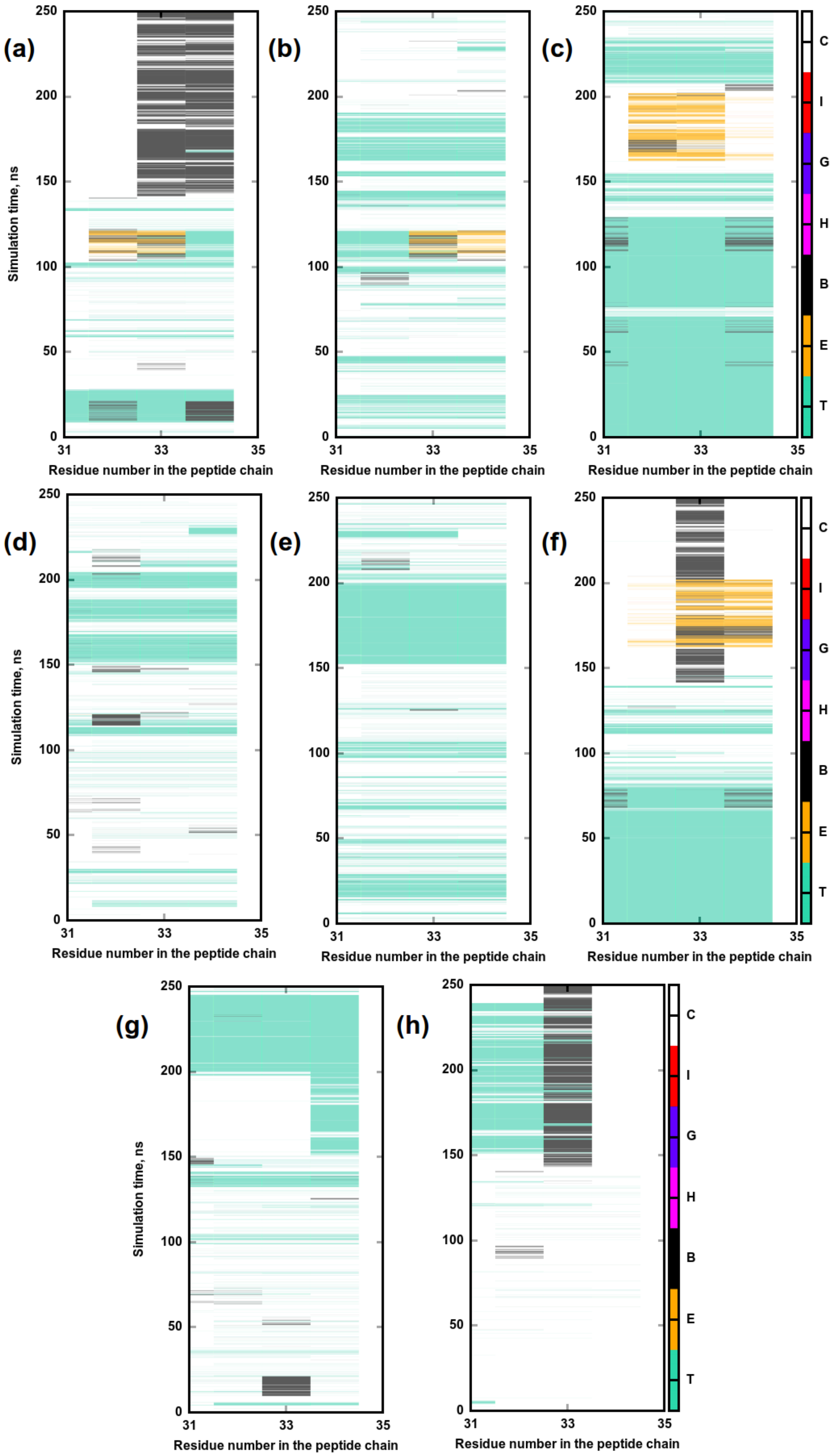

Figure S30: Secondary structures of peptides for systems containing 8 A$\beta(31-35)$ without CBD during production runs. Letters (a)-(h) denote separate peptides. T - turn, E - extended conformation, B - isolated β-bridge, H - α-helix, G - 3_{10}-helix, I - π-helix, C - coil.

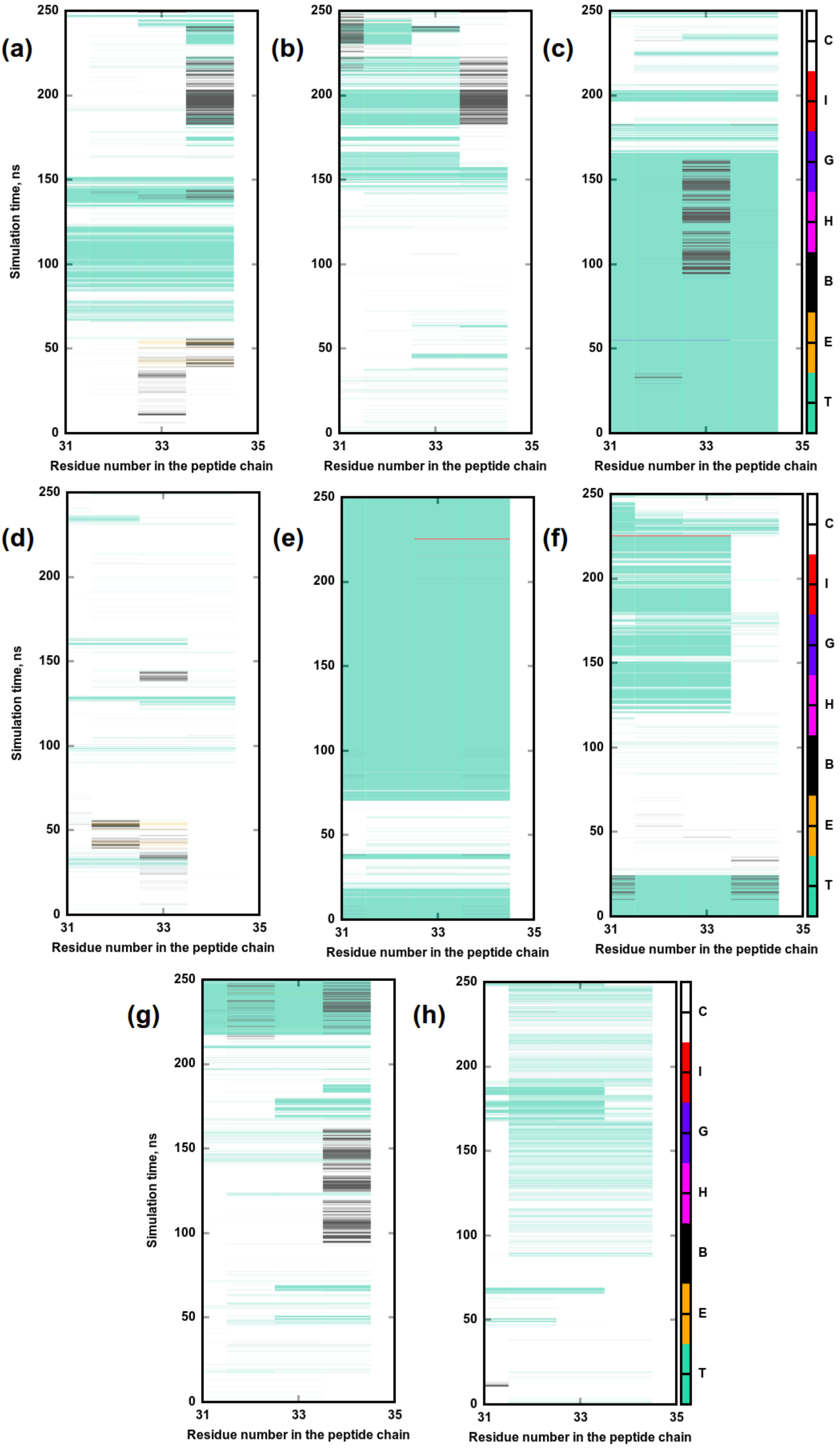

Figure S31: Secondary structures of peptides for systems containing 8 Aβ(31 − 35) with 8 CBD molecules during production runs. Letters (a)-(h) denote separate peptides. T - turn, E - extended conformation, B - isolated β-bridge, H - α-helix, G - 3_{10}-helix, I - π-helix, C - coil.

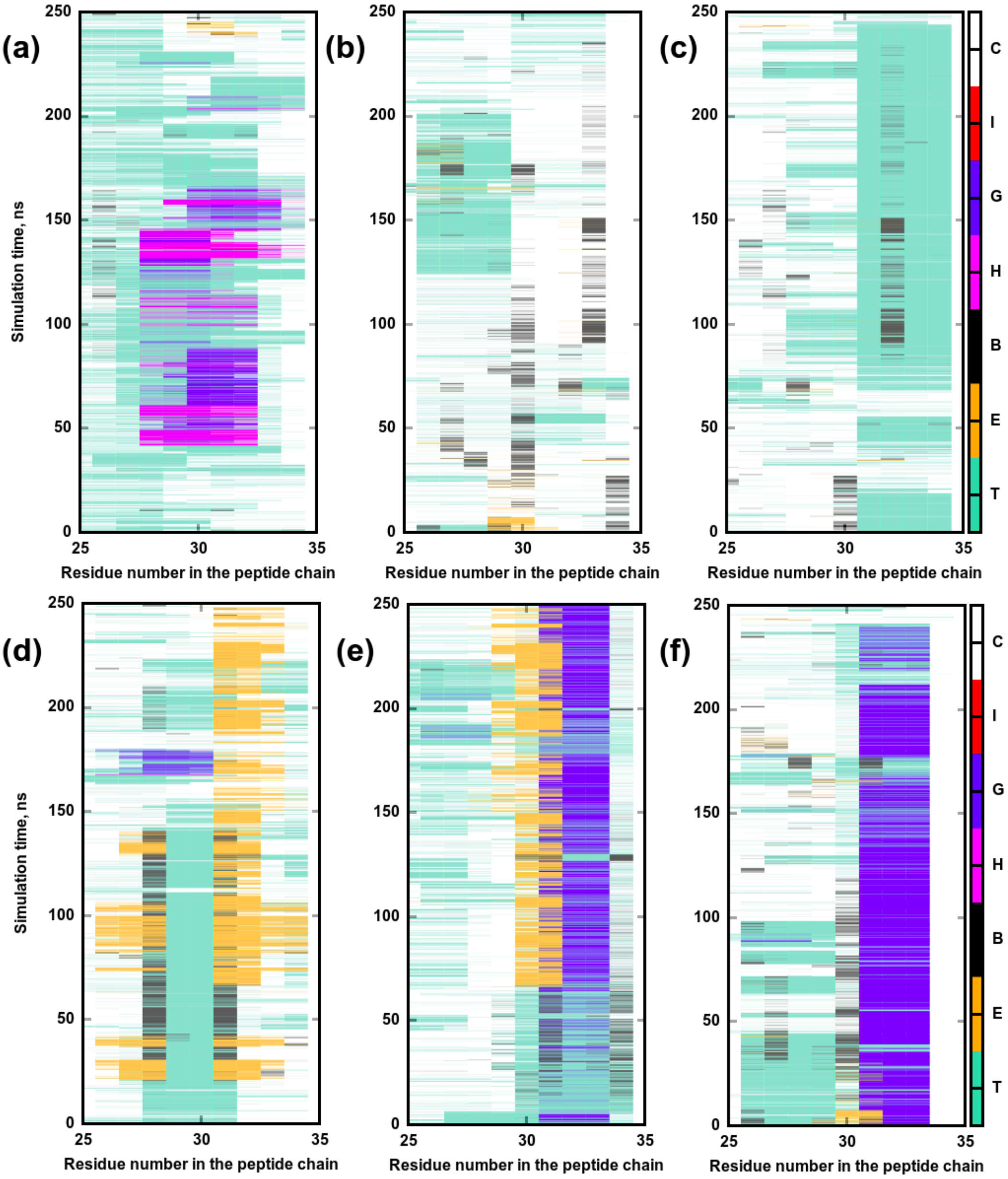

Figure S32: Secondary structures of peptides for systems containing 6 A$\beta(25-35)$ without CBD during production runs. Letters (a)-(f) denote separate peptides. T - turn, E - extended conformation, B - isolated β-bridge, H - α-helix, G - 3_{10}-helix, I - π-helix, C - coil.

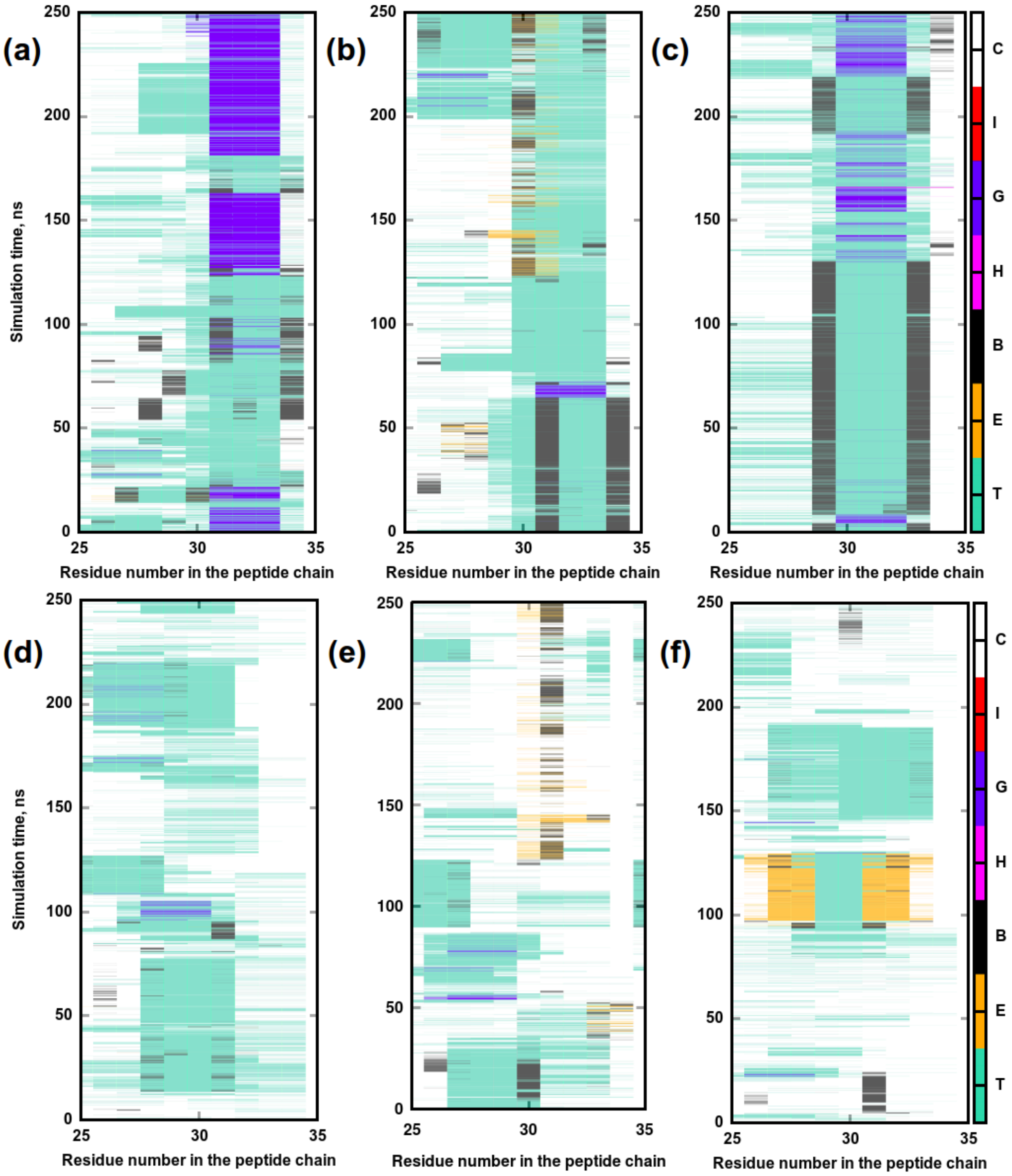

Figure S33: Secondary structures of peptides for systems containing 6 A$\beta(25-35)$ with 6 CBD molecules during production runs. Letters (a)-(f) denote separate peptides. T - turn, E - extended conformation, B - isolated β-bridge, H - α-helix, G - 3_{10}-helix, I - π-helix, C - coil.

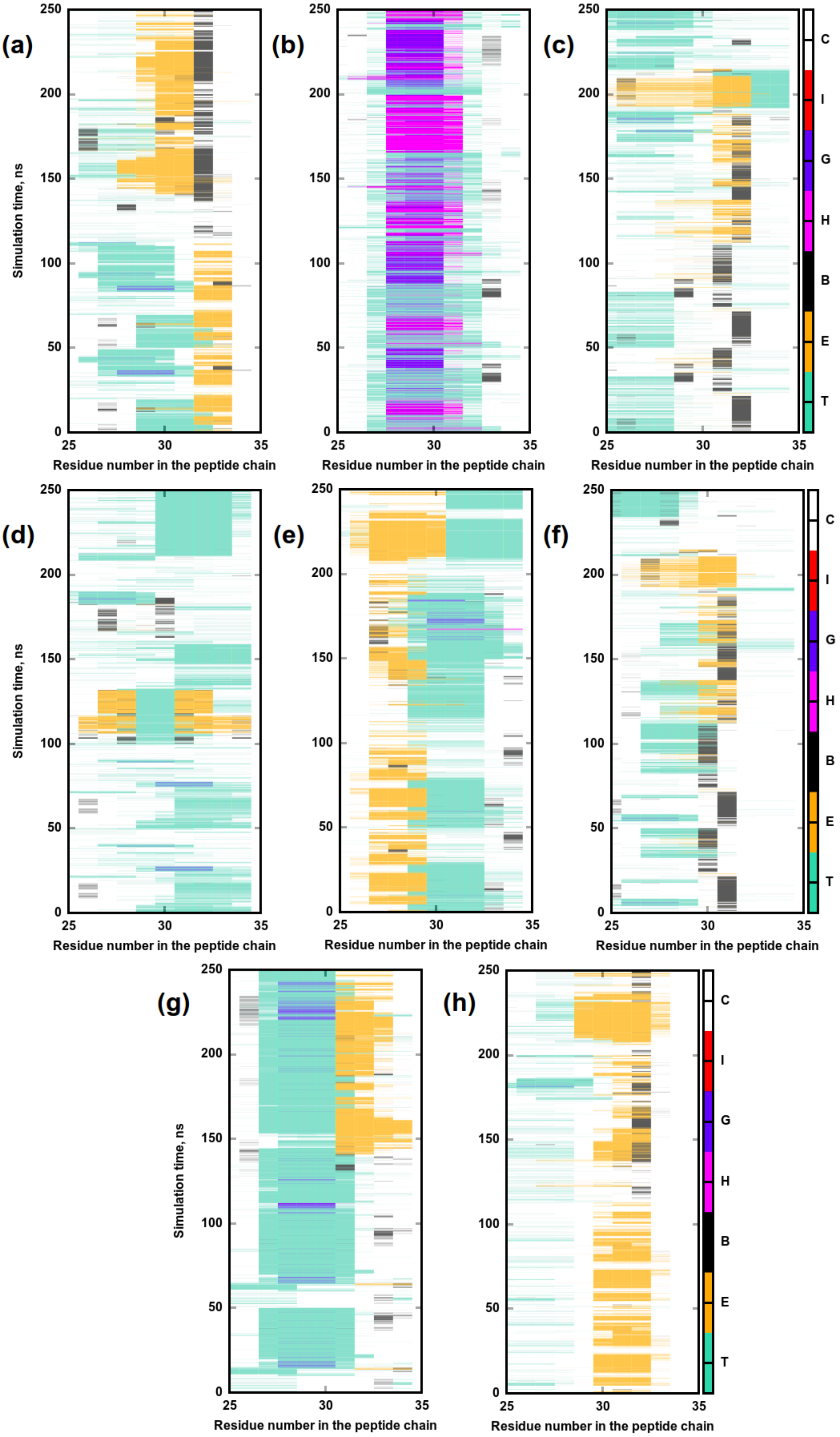

Figure S34: Secondary structures of peptides for systems containing 8 Aβ(25 − 35) without CBD during production runs. Letters (a)-(h) denote separate peptides. T - turn, E - extended conformation, B - isolated β-bridge, H - α-helix, G - 3_{10}-helix, I - π-helix, C - coil.

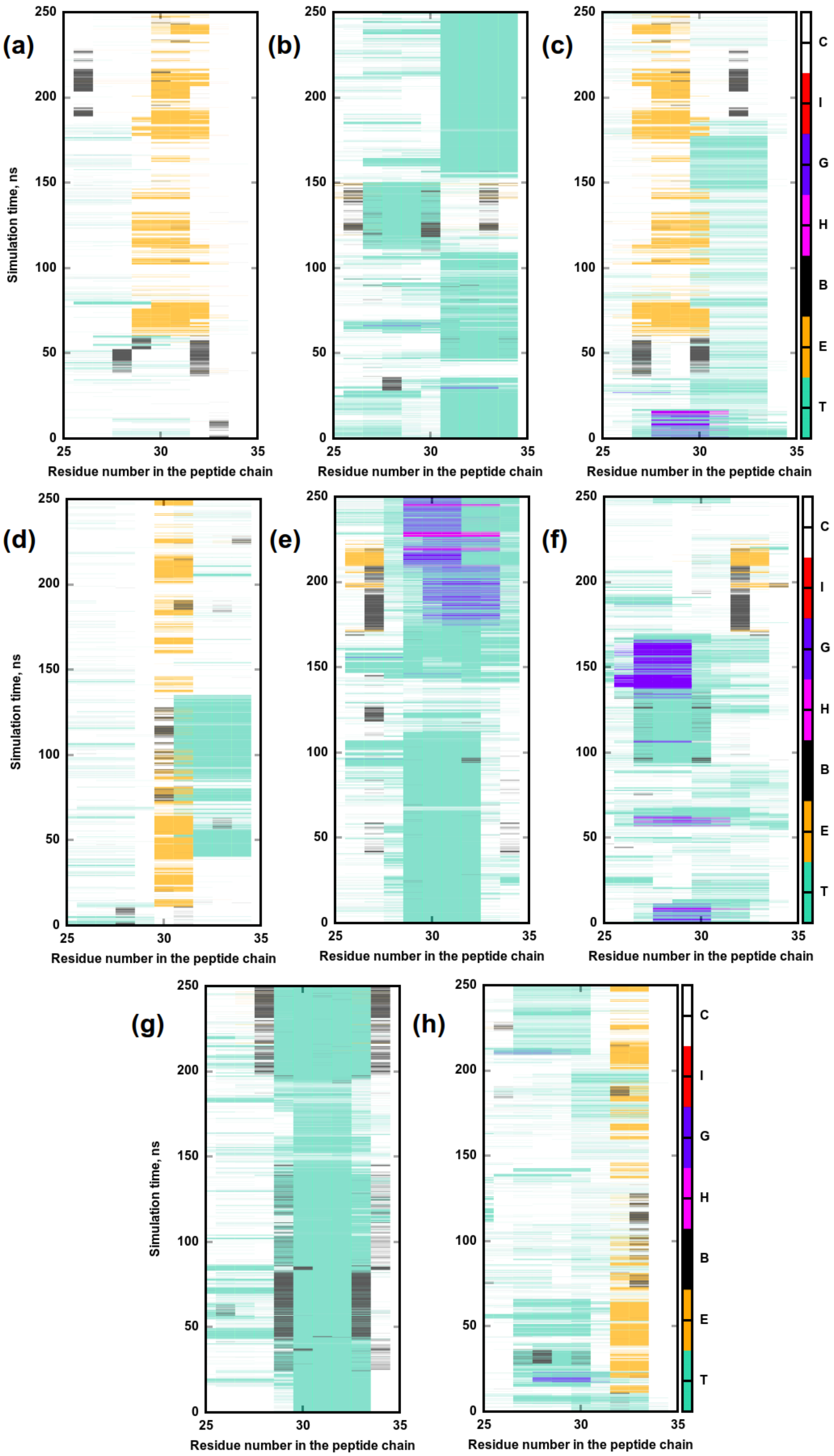

Figure S35: Secondary structures of peptides for systems containing 8 Aβ(25 − 35) with 8 CBD molecules during production runs. Letters (a)-(h) denote separate peptides. T - turn, E - extended conformation, B - isolated β-bridge, H - α-helix, G - 3_{10}-helix, I - π-helix, C - coil.

2 Well-tempered MetaD

2.1 Final dimensions of simulation boxes

Table S2: Dimensions of simulation boxes (in nm) after the equilibration (classical MD) and molar concentrations (c) of components (computed using the equation S1, in mol/m^3).

System	x	y	z	c(Aβ)	c(CBD)	c(Na)
2 A$\beta(31-35)$	6.75	6.75	6.75	10.80	none	none
2 A$\beta(31-35)$ + 1 CBD	6.76	6.76	6.76	10.75	5.38	none
2 A$\beta(31-35)$ + 2 CBD	6.76	6.76	6.76	10.75	10.75	none
2 A$\beta(25-35)$	6.76	6.76	6.76	10.75	none	10.75
2 A$\beta(25-35)$ + 1 CBD	6.77	6.77	6.77	10.70	5.35	10.70
2 A$\beta(25-35)$ + 2 CBD	6.77	6.77	6.77	10.70	10.70	10.70

2.2 Discussion about the quality of sampling and convergence

When presenting results from well-tempered metadynamics it is important to have good sampling and relatively small error. In the case of Aβ peptides of various lengths and conformations such simulations shall be long enough in order to obtain reliable information.

Simulations with 1 CV for 2 peptides were carried out for 7 μs. Figure S36 demonstrates the evolution of the CV (Figure S36 (a)) in time as well as the evolution of the secondary structure of A$\beta(31-35)$ (Figure S36 (b)-(c)). Turn and coil were the most common secondary structures during the whole simulation time. However, there is a remarkable appearance of the isolated β-bridge when two peptides were located close to each other. Quite few appearances of the 3_{10}-helix were detected, but their number was not so significant, comparing to the number of other secondary structures. For A$\beta(25-35)$ the number of different conformations was larger, due to its longer length (Figure S37). When there is a short distance between the peptides there are several well represented secondary structures, such as the extended conformation in combination with turn and coil and the isolated β-bridge with turn and coil. Less probable combinations were with the α-helix and coil and the 3_{10}-helix with turn and coil (Figure S37).

There were more degrees of freedom in simulations with 2 peptides at various amounts of CBD. In the case of two peptides and 1 CBD molecule the distance between the second peptide and CBD was not considered for the calculation of PMF. In simulations with 2 peptides and 2 CBD molecules more distances were missing (see Figure 2 in the main text). Such missing variables may raise questions about the sampling. In order to verify that the missing variables are not affecting the statistics negatively (for example, if one molecule would be clustered to another one during the whole simulation time), the missing distances were computed during the whole simulation time for every calculation. Figures S38-S43 present the sampling for simulations containing various amounts of CBD. Regardless of the differences in composition similar varieties of the secondary structure were observed as in computations with 1 CV (Figures S38-S43). Moreover, at the same combinations of secondary structures peptides were clustering as in the cases with one dimensional simulations.

Then since the quality of sampling was good enough the question about convergence arises. Indeed, one can always improve the statistics by continuing a simulation, but considering deficiencies of modern FF and the lack of computational resources, one can certainly stop the simulation if changes in PMF fulfill the imposed threshold. In this work the threshold for considering the simulation completed was that the average value of the error during the last 3 μs should not exceed 1 kJ/mol per 1 μs. For every CV such profiles were computed every 5 ns for selected points of local minimas. The corresponding convergence plots are shown in Figures S44-S47.

Another way of investigating the convergence is to look at the evolution of the Gaussian height during the simulation time. Figures S48-S50 demonstrate changes of the Gaussian height for every CV. In one dimensional calculations containing only peptides the profile values are equal to zero after approximately 500 ns. However, this can not insure that higher values can not appear afterwards. During the last 2 μs of simulations with various amounts of CBD molecules the Gaussian heights are appearing to be about 0.01-0.2 kJ/mol with an average value close to 0.02 kJ/mol. As in the case of the convergence plots one has to select a threshold value for the Gaussian height to determine when the calculation can be considered as complete. The reason for this is that with more than one CV and with several "hidden" variables, like distances between other molecules and their conformations, the appearance of Gaussian heights larger than zero will continue during the simulation.

2.3 Quality of sampling: figures for collective variables, secondary structures of peptides etc.

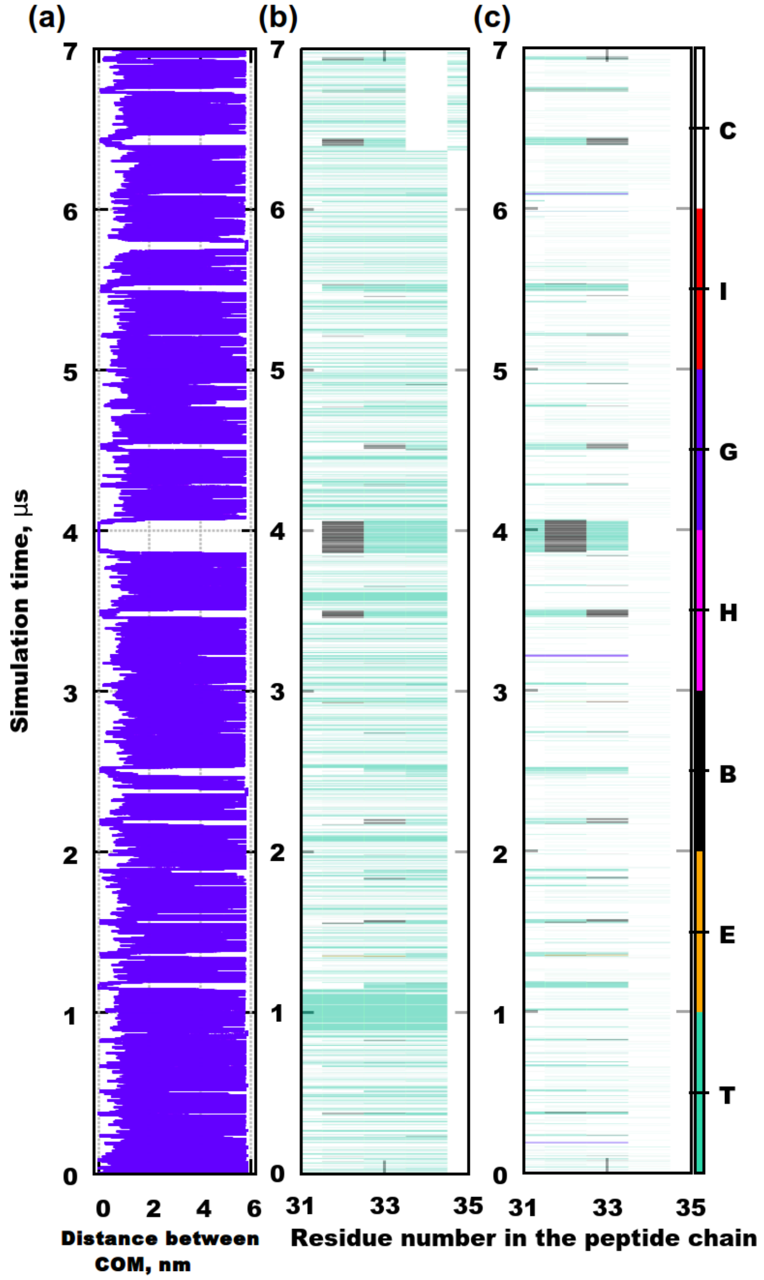

Figure S36: Quality of sampling for the system containing only Aβ(31−35) in water. (a) Evolution of the CV over the simulation time. (b) and (c) show the evolution of secondary structures of peptides 1 and 2 respectively over the simulation time. T - turn, E - extended conformation, B - isolated β-bridge, H - α-helix, G - 3_{10}-helix, I - π-helix, C - coil.

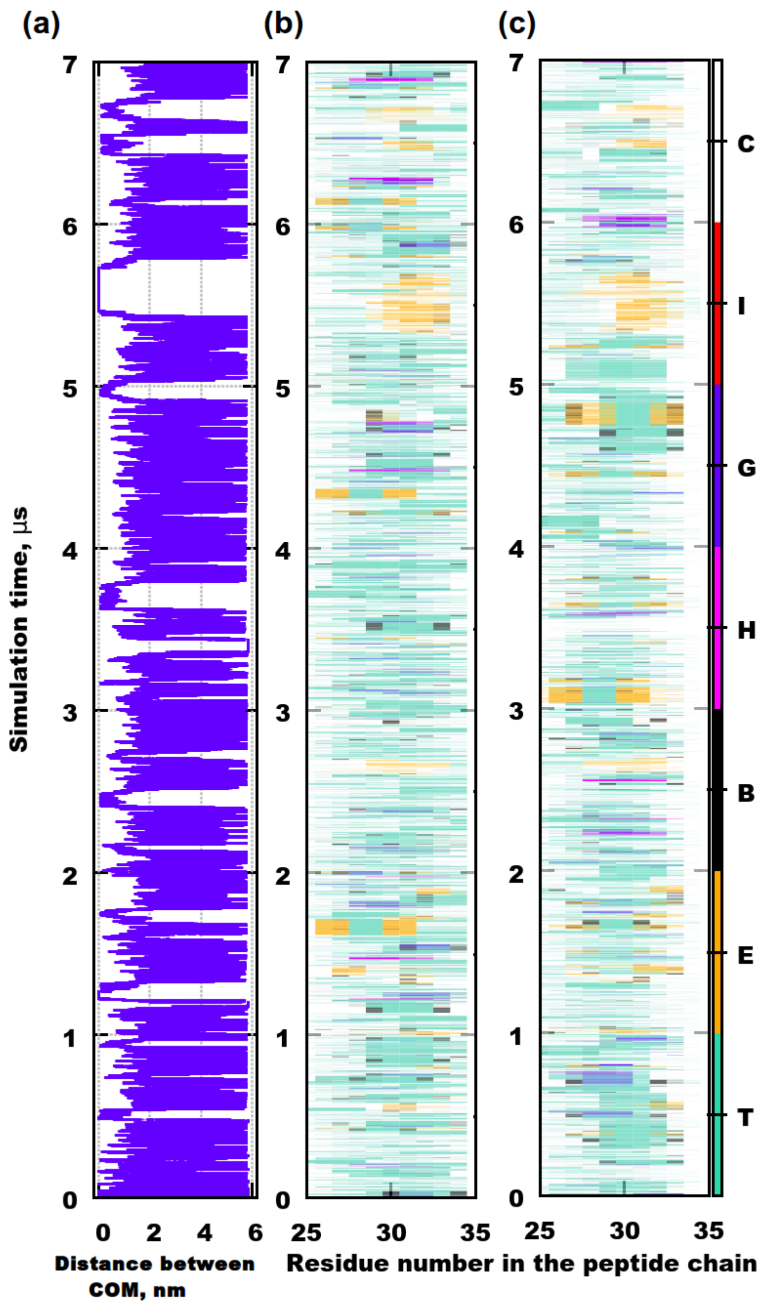

Figure S37: Quality of sampling for the system containing only Aβ(25$-$35) in water. (a) Evolution of the CV over the simulation time. (b) and (c) show the evolution of secondary structures of peptides 1 and 2 respectively over the simulation time. T - turn, E - extended conformation, B - isolated β-bridge, H - α-helix, G - 3_{10}-helix, I - π-helix, C - coil.

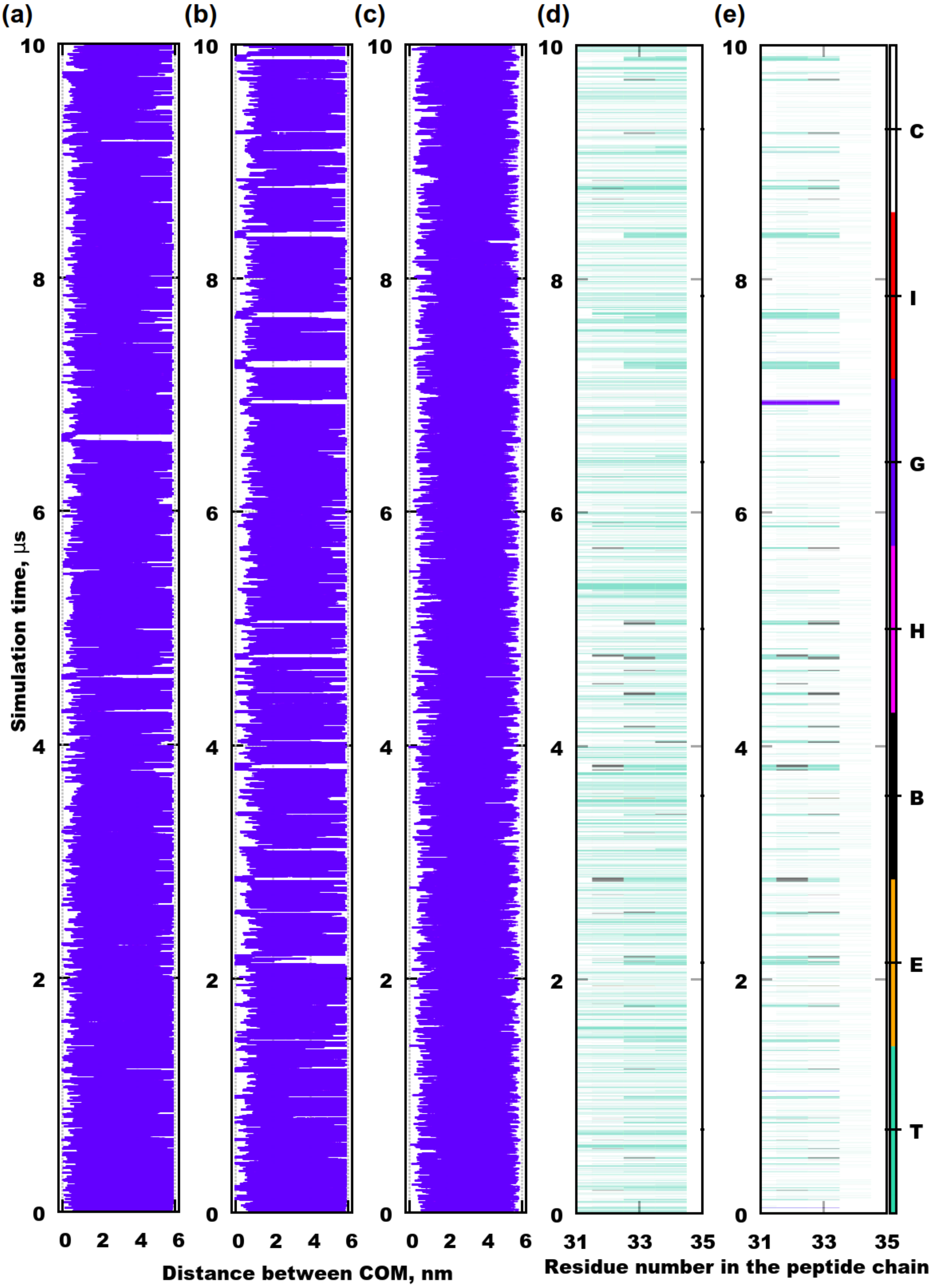

Figure S38: Quality of sampling for the system containing Aβ(31 − 35) and 1 CBD in water. (a) Evolution of the distance between the peptide center of mass over the simulation time (CV1). (b) Evolution of the distance between the center of mass of peptide 1 and CBD molecule (CV2). (c) Evolution of the distance between the center of mass of peptide 2 and CBD molecule (not declared as a CV). (d) and (e) show the evolution of peptide secondary structures during the simulation time. T - turn, E - extended conformation, B - isolated β-bridge, H - α-helix, G - 3_{10}-helix, I - π-helix, C - coil.

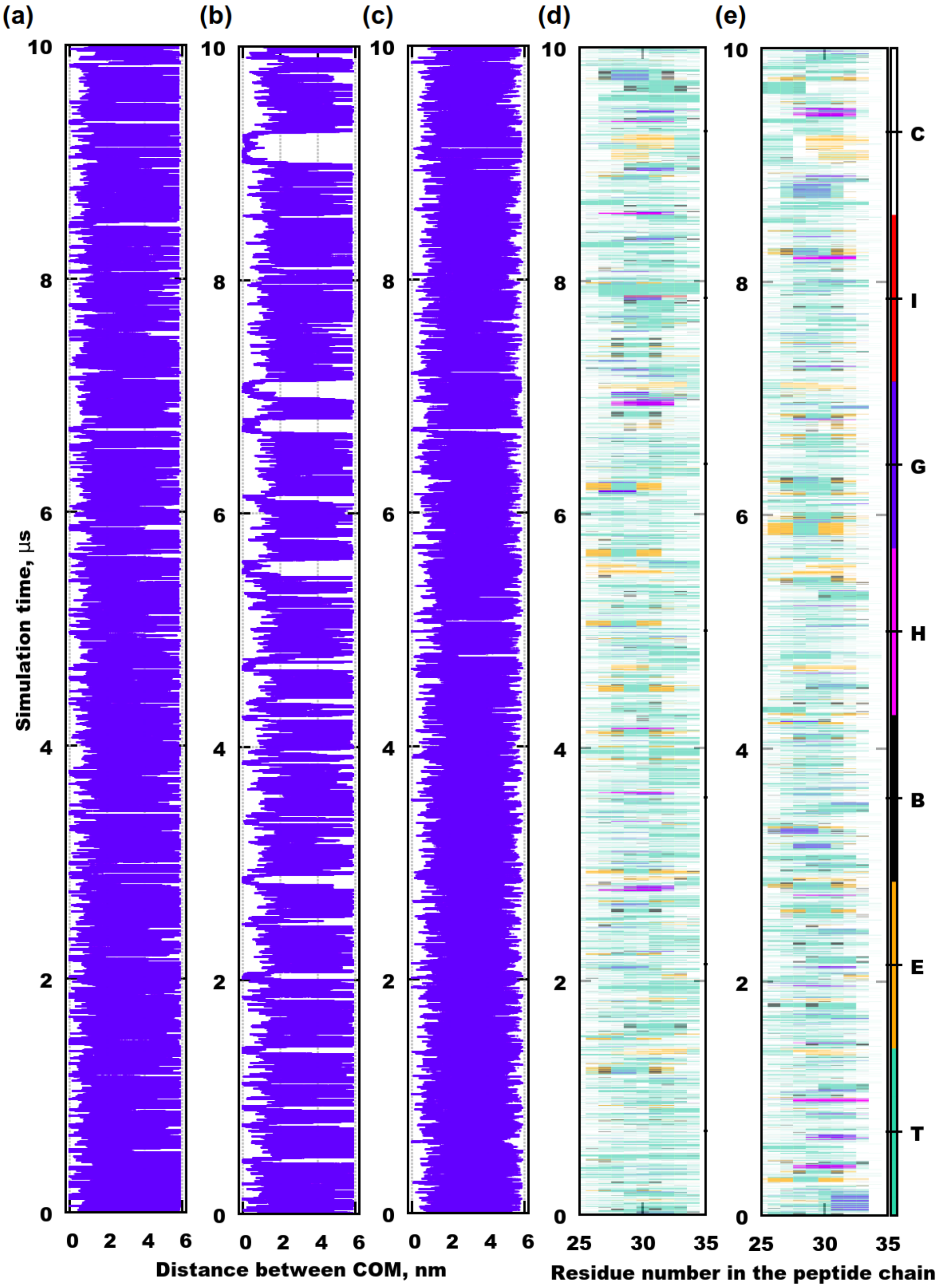

Figure S39: Quality of sampling for the system containing Aβ(25 − 35) and 1 CBD in water. (a) Evolution of the distance between the peptide center of mass over the simulation time (CV1). (b) Evolution of the distance between the center of mass of peptide 1 and CBD molecule (CV2). (c) Evolution of the distance between the center of mass of peptide 2 and CBD molecule (not declared as a CV). (d) and (e) show the evolution of peptide secondary structures during the simulation time. T - turn, E - extended conformation, B - isolated β-bridge, H - α-helix, G - 3_{10}-helix, I - π-helix, C - coil.

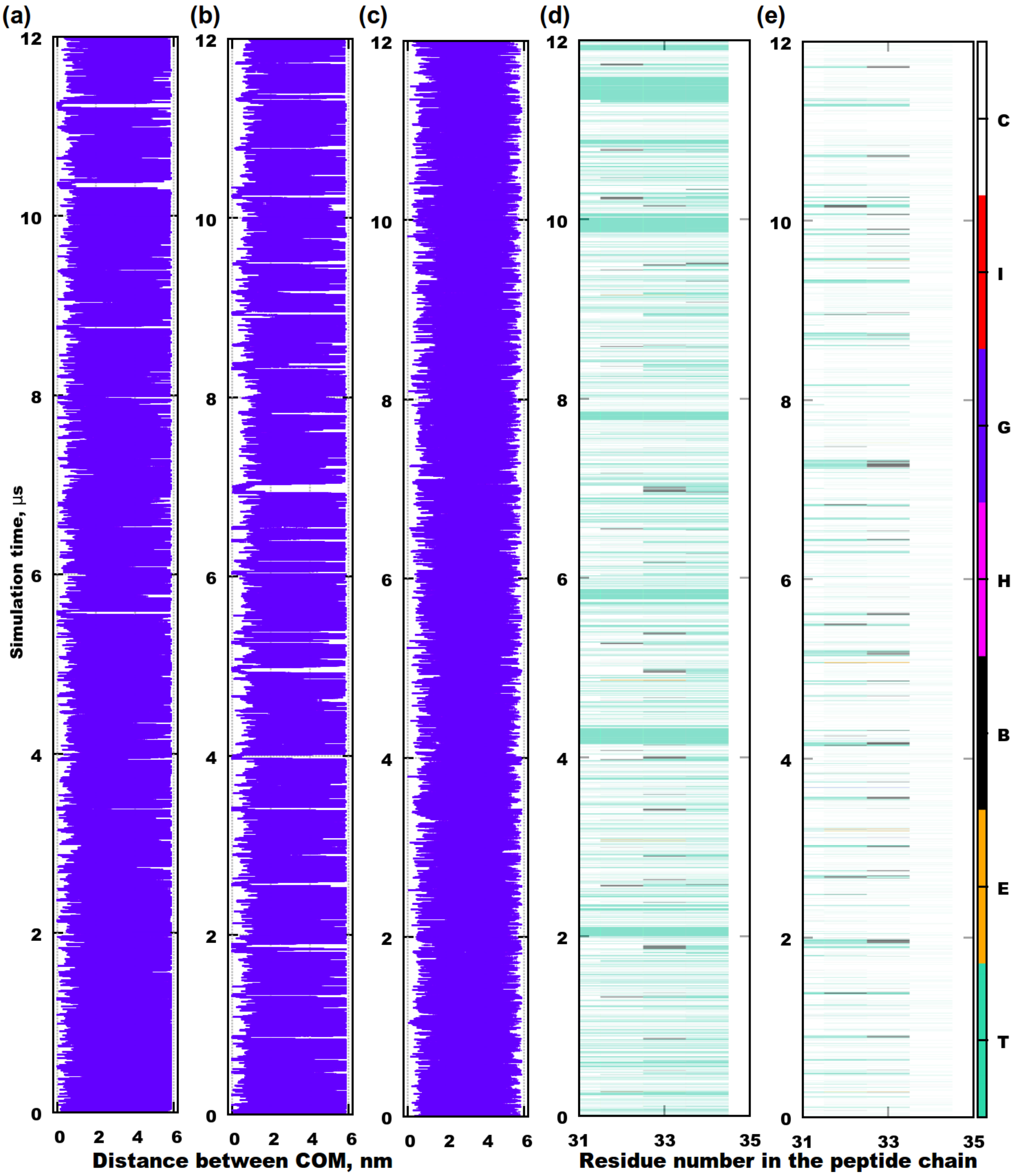

Figure S40: PART 1: Quality of sampling for the system containing A$\beta(31-35)$ and 2 CBD in water. (a) Evolution of the distance between the peptide center of mass over the simulation time (CV1). (b) Evolution of the distance between the center of mass of peptide 1 and CBD molecule (CV2). (c) Evolution of the distance between the center of mass of peptide 2 and CBD molecule (not declared as a CV). (d) and (e) show the evolution of peptide secondary structures during the simulation time. T - turn, E - extended conformation, B - isolated β-bridge, H - α-helix, G - 3_{10}-helix, I - π-helix, C - coil.

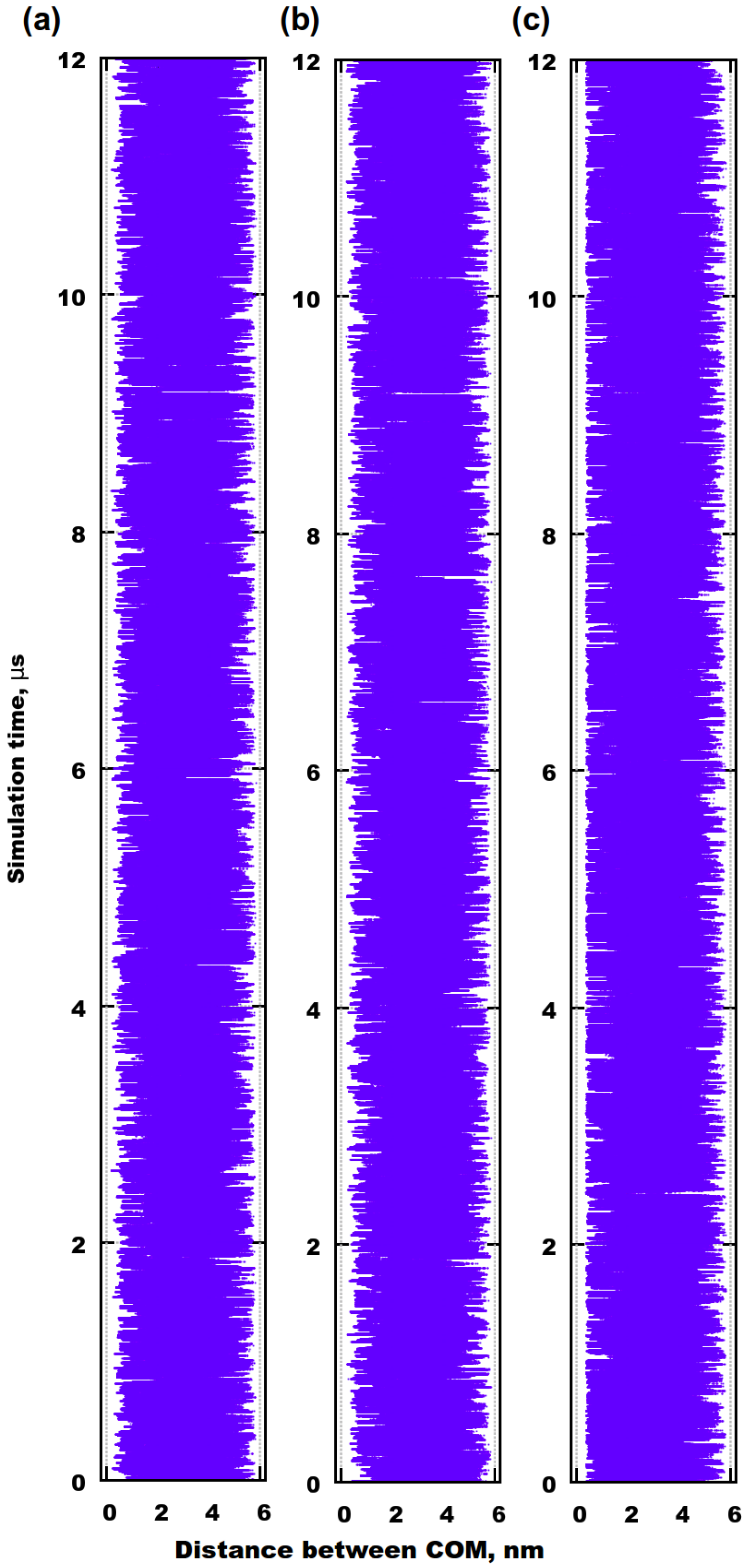

Figure S41: PART 2: Quality of sampling for the system containing Aβ(31 − 35) and 2 CBD in water. (a) Evolution of the distance between the centers of mass of the peptide-1 and CBD-2. (b) Evolution of the distance between the centers of mass of the peptide-2 and CBD-2. (c) Evolution of the distance between the centers of mass of CBD molecules.

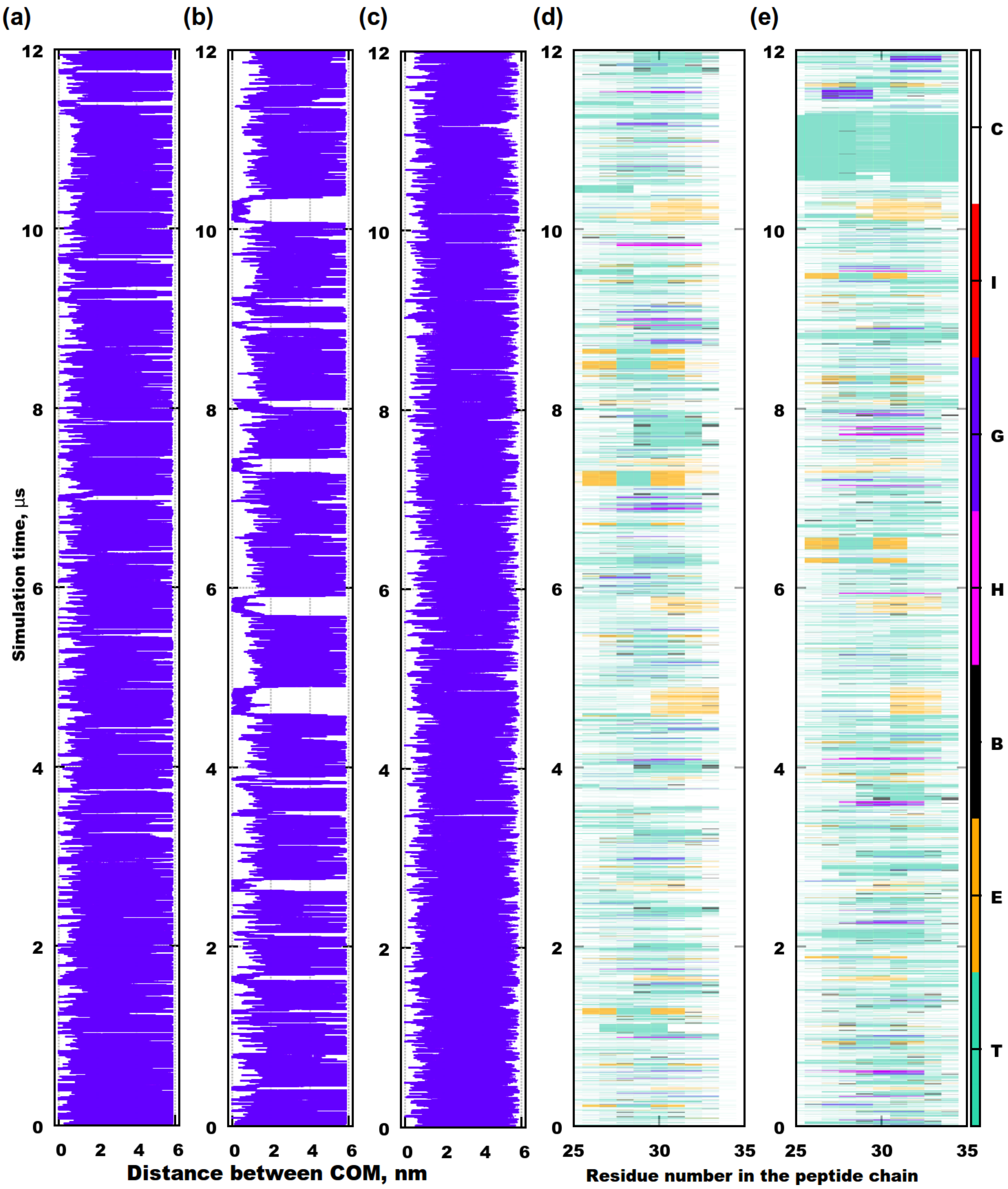

Figure S42: PART 1: Quality of sampling for the system containing Aβ(25 $-$ 35) and 2 CBD in water. (a) Evolution of the distance between the peptide center of mass over the simulation time (CV1). (b) Evolution of the distance between the center of mass of peptide 1 and CBD molecule (CV2). (c) Evolution of the distance between the center of mass of peptide 2 and CBD molecule (not declared as a CV). (d) and (e) show the evolution of peptide secondary structures during the simulation time. T - turn, E - extended conformation, B - isolated β-bridge, H - α-helix, G - 3_{10}-helix, I - π-helix, C - coil.

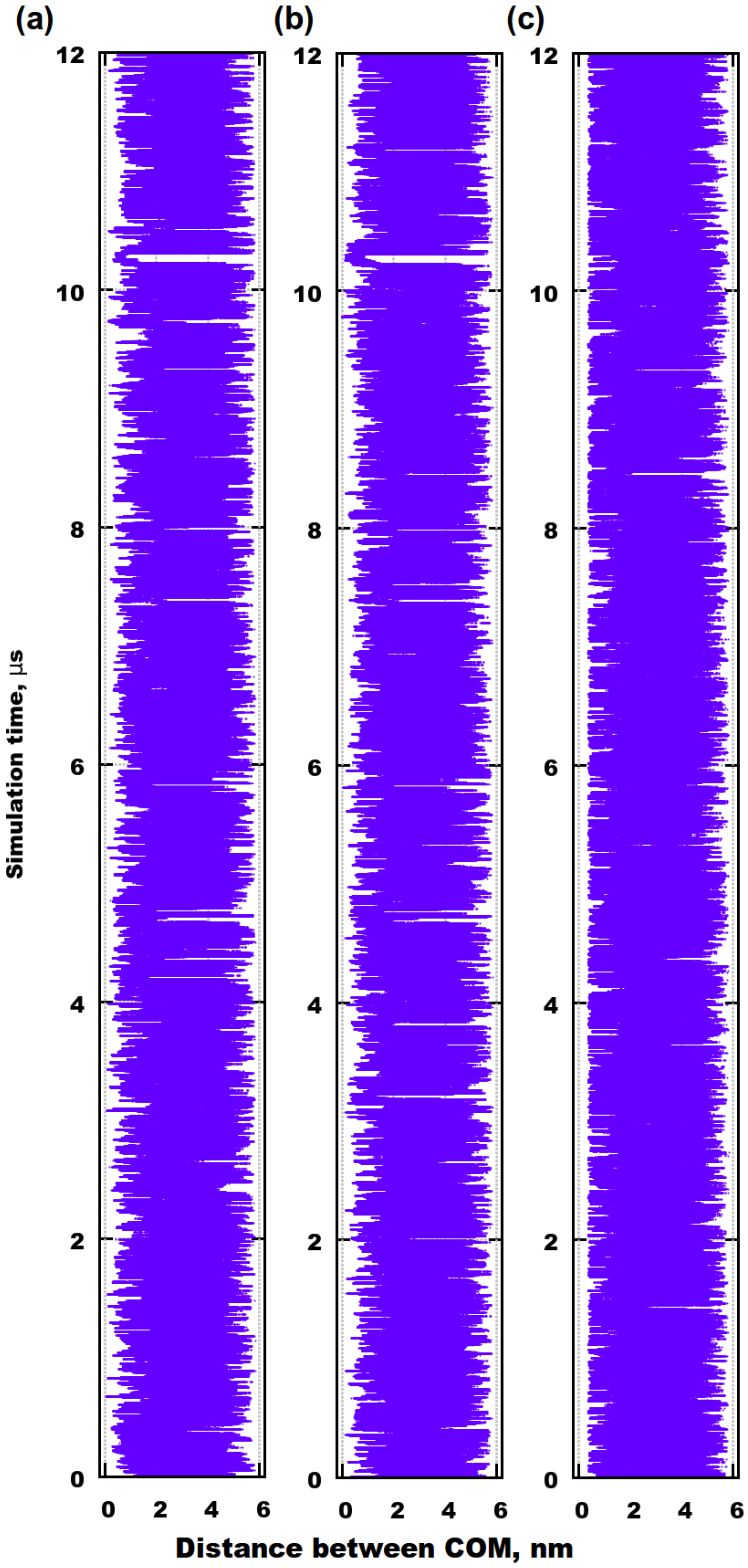

Figure S43: PART 2: Quality of sampling for the system containing Aβ(25 − 35) and 2 CBD in water. (a) Evolution of the distance between the centers of mass of the peptide-1 and CBD-2. (b) Evolution of the distance between the centers of mass of the peptide-2 and CBD-2. (c) Evolution of the distance between the centers of mass of CBD molecules.

2.4 Convergence studies

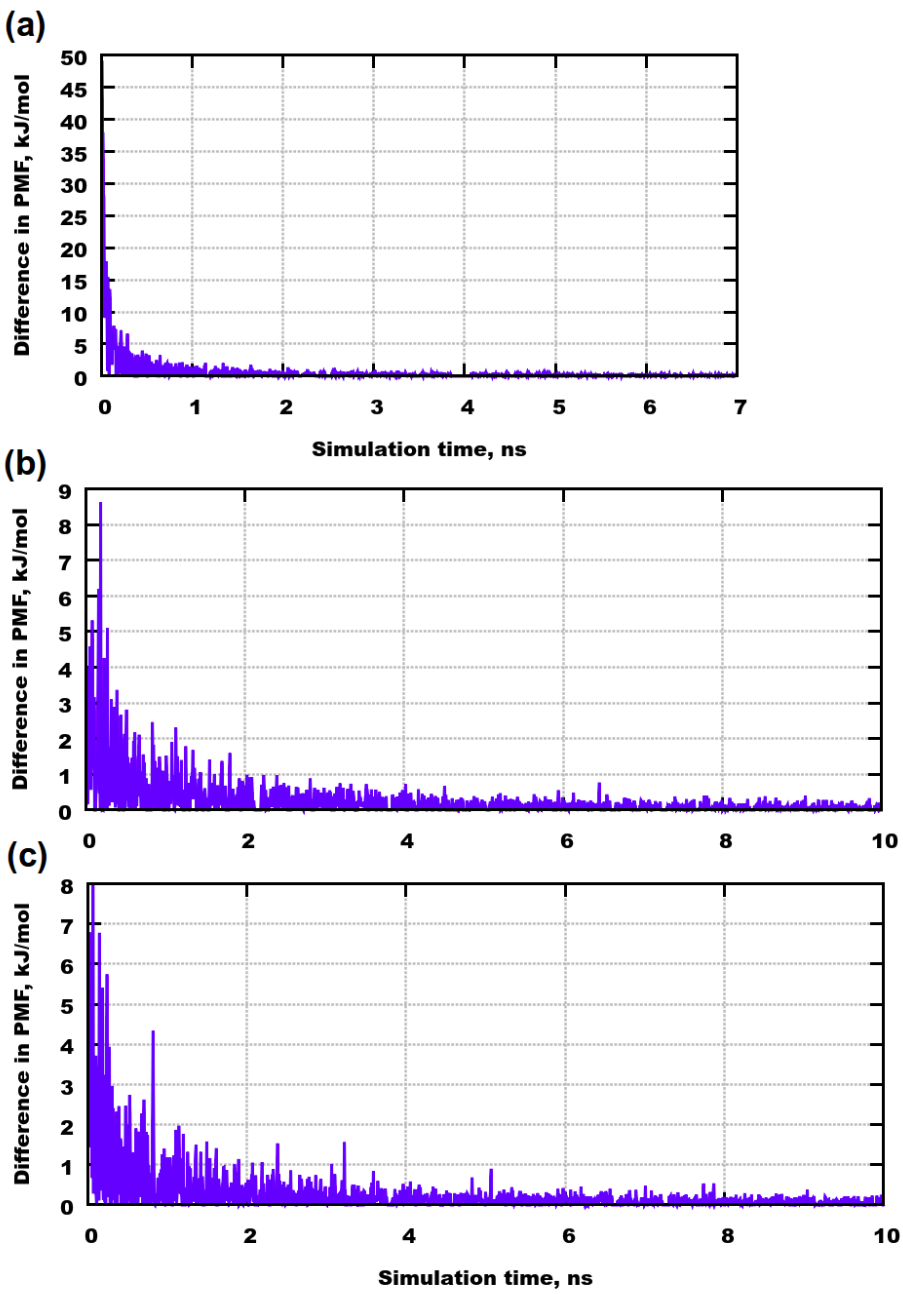

Figure S44: Convergence plots (a) System with a Aβ(31 − 35) (b) System with 2 Aβ(31 − 35) and 1 CBD: CV1 (b) System with 2 Aβ(31 − 35) and 1 CBD: CV2.

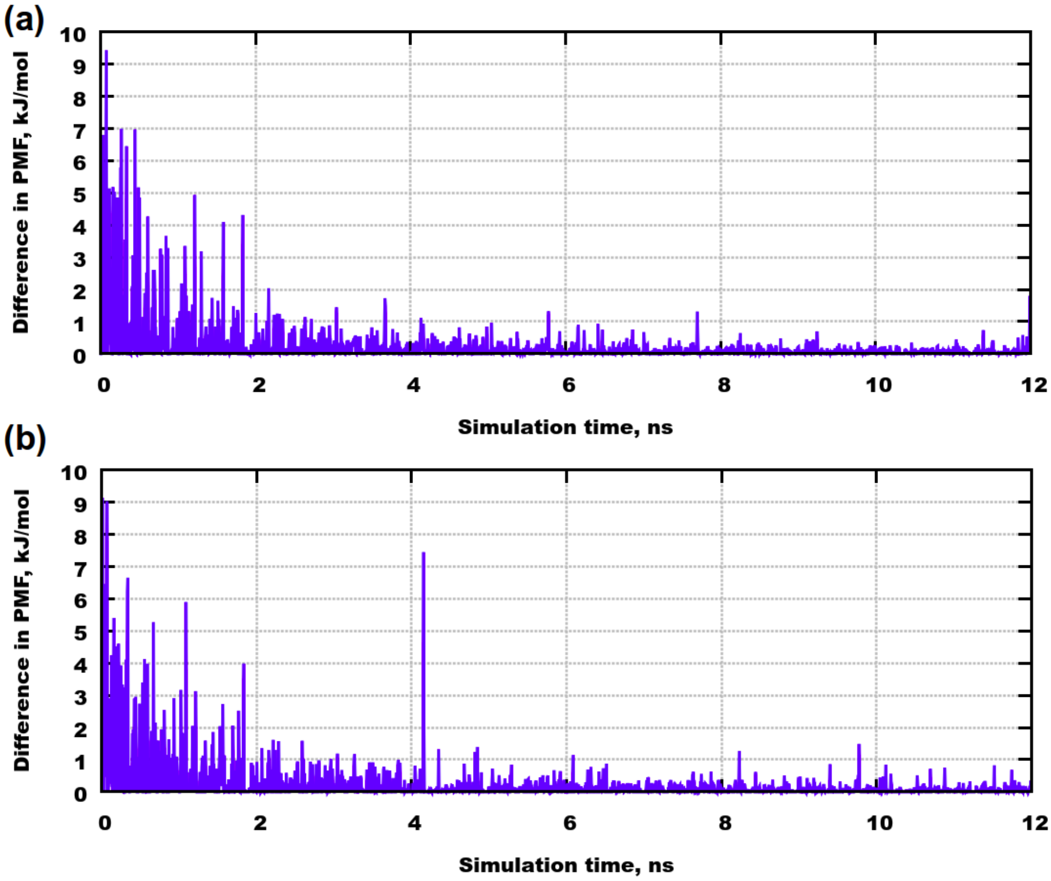

Figure S45: Convergence plots for the system with 2 A$\beta(31-35)$ and 2 CBD (a) CV1 (b) CV2.

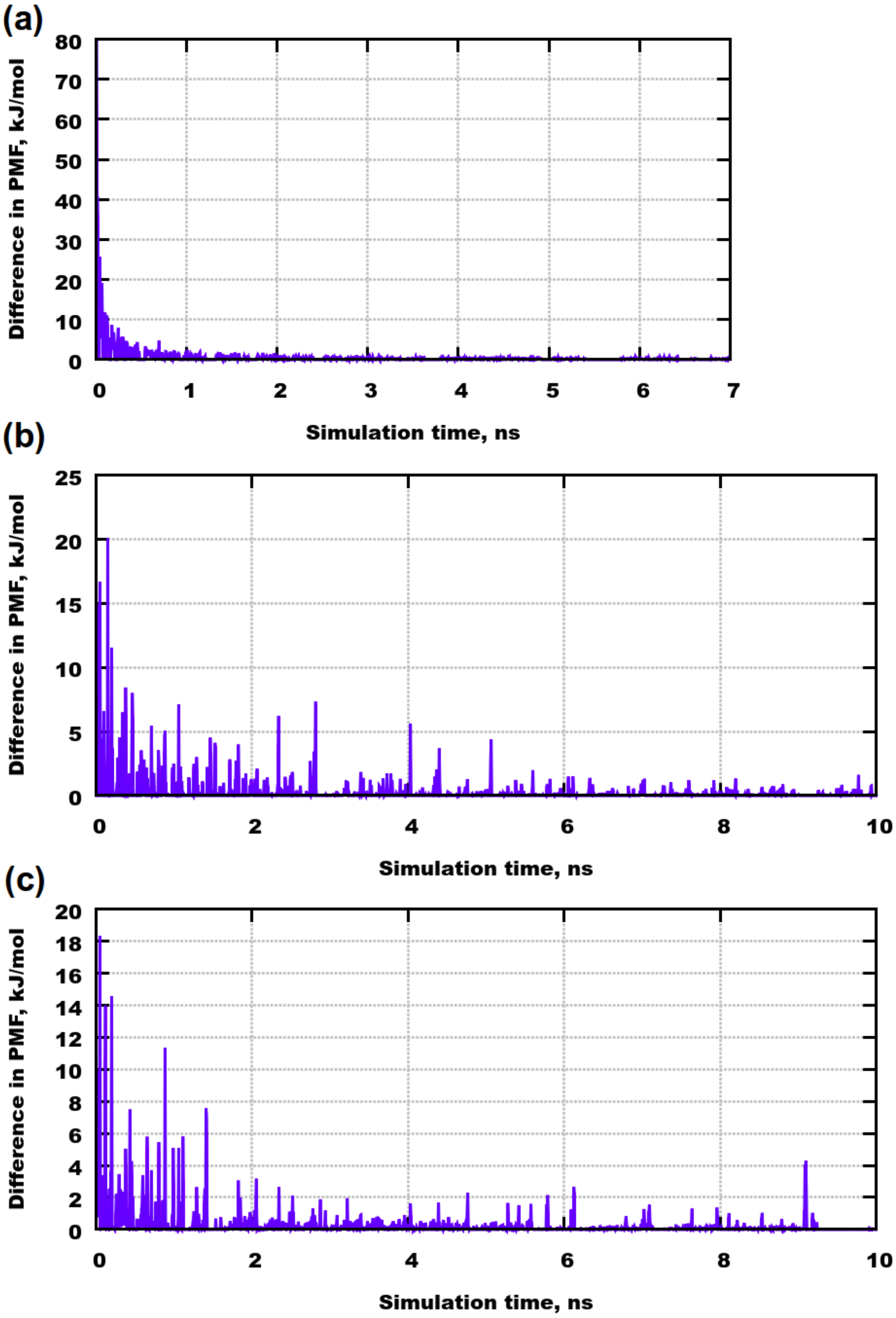

Figure S46: Convergence plots (a) System with a Aβ(25 − 35) (b) System with 2 Aβ(25 − 35) and 1 CBD: CV1 (b) System with 2 Aβ(25 − 35) and 1 CBD: CV2.

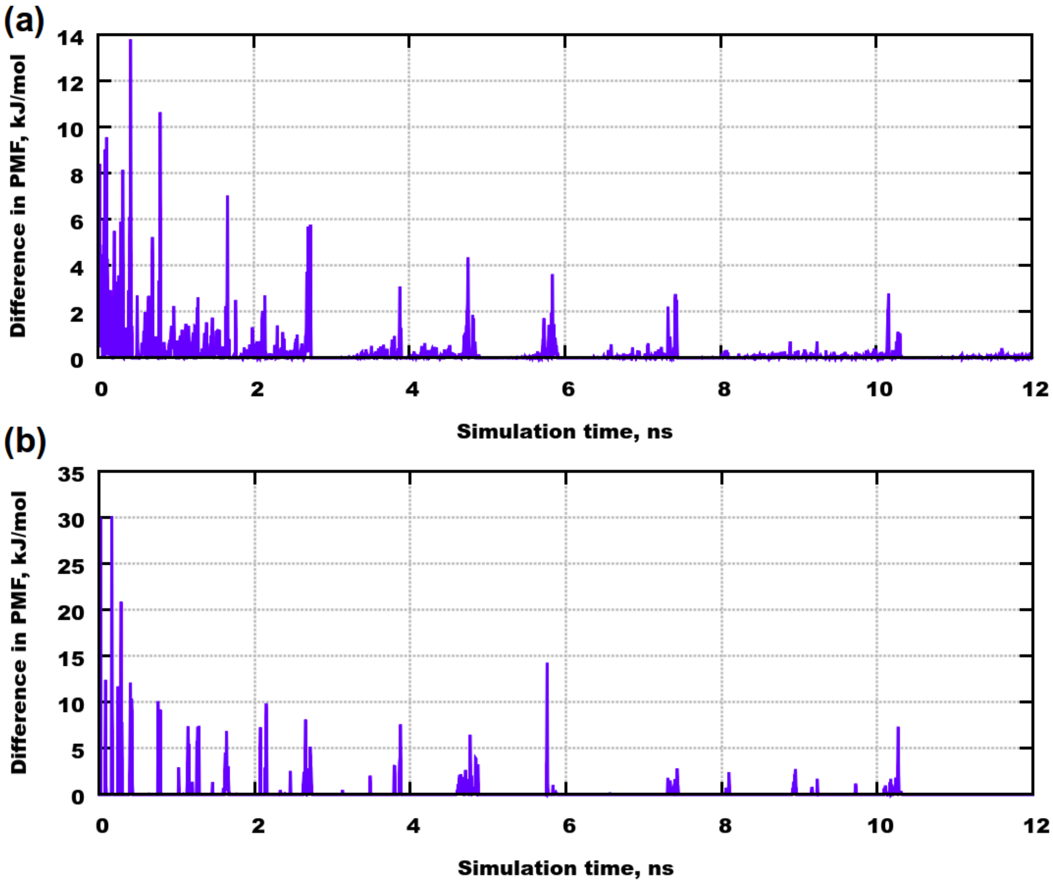

Figure S47: Convergence plots for the system with 2 Aβ(25 − 35) and 2 CBD (a) CV1 (b) CV2.

2.5 Printouts of HILLS-files

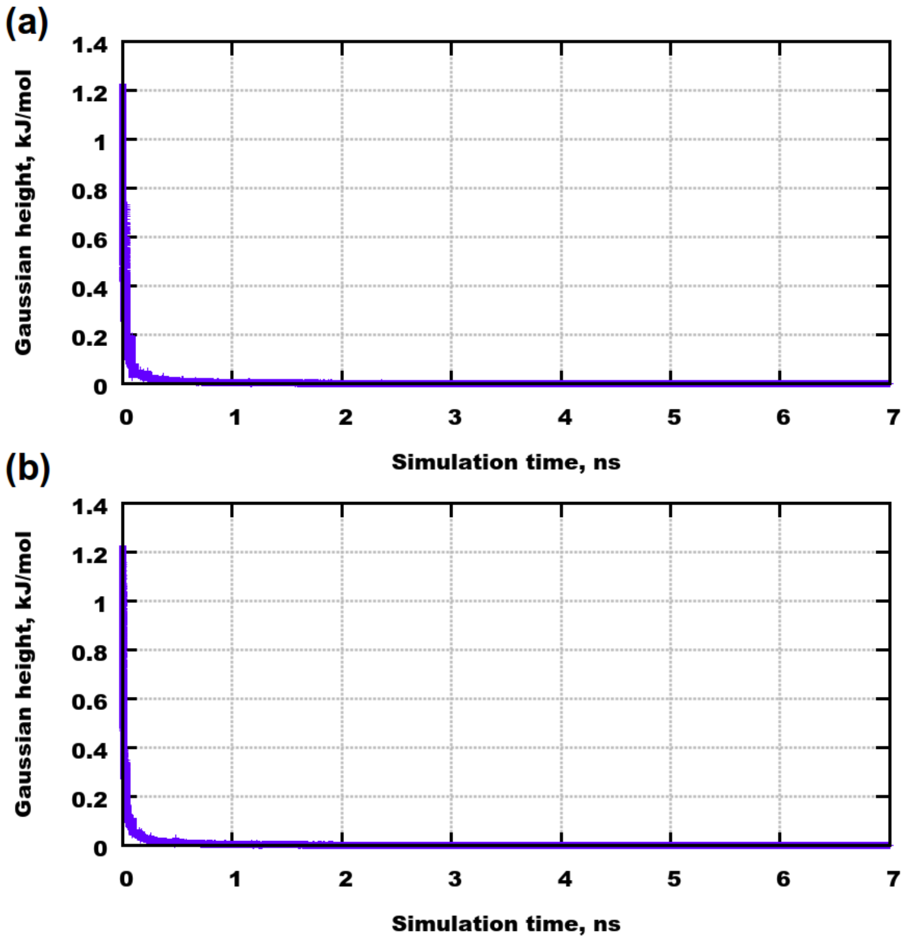

Figure S48: Printout for HILLS-files (a) Aβ(31 − 35) (b) Aβ(25 − 35).

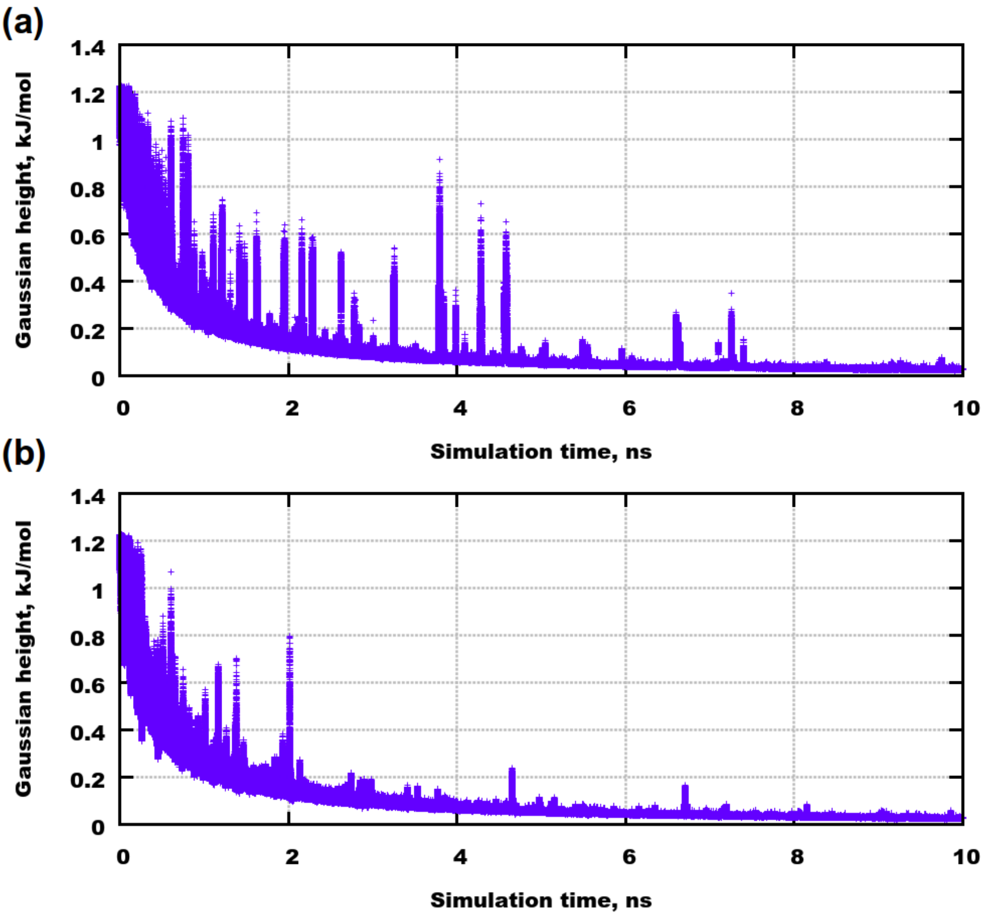

Figure S49: Printout for HILLS-files (1 CBD) (a) A$\beta(31-35)$ (b) A$\beta(25-35)$.

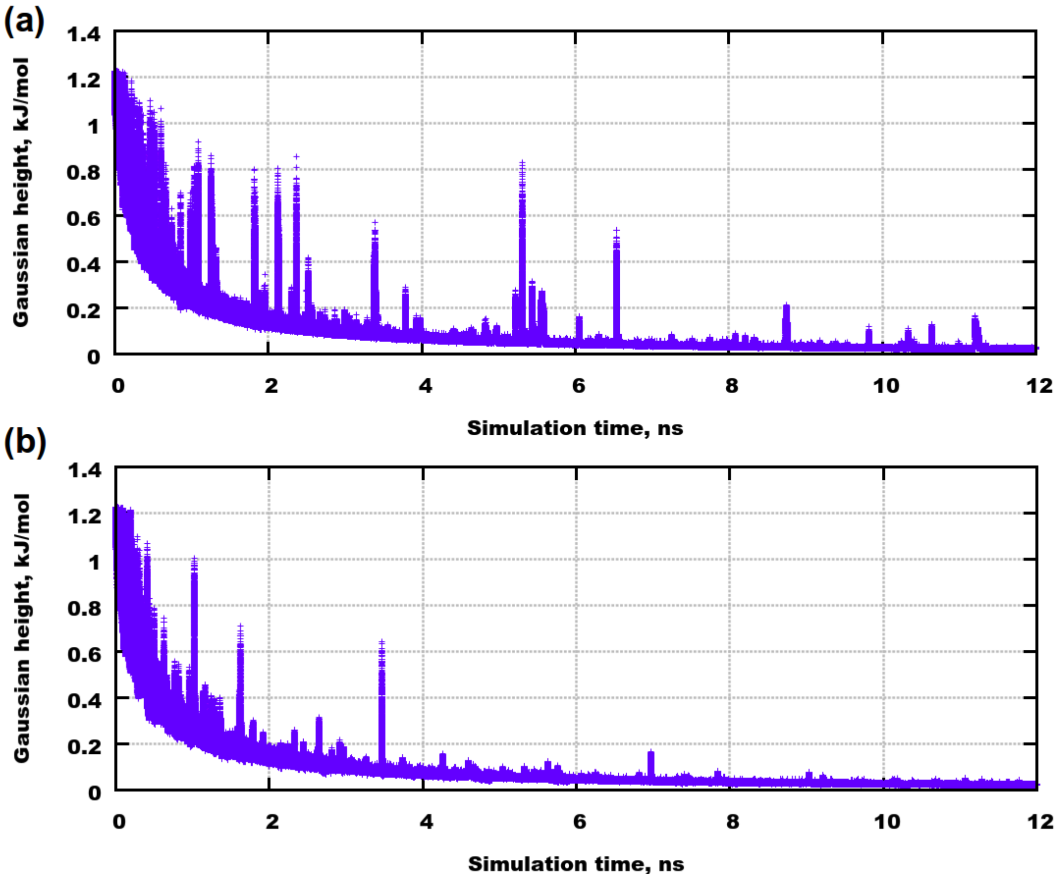

Figure S50: Printout for HILLS-files (2 CBD) (a) A$\beta(31-35)$ (b) A$\beta(25-35)$.
